# Traceless Solid-Phase Synthesis of [6,7,8 + 5,6,7]-Fused Molecular Frameworks

**DOI:** 10.3390/molecules23051090

**Published:** 2018-05-04

**Authors:** Vanesa Giménez-Navarro, Viktor Krchňák

**Affiliations:** 1 Department of Organic Chemistry, Faculty of Science, Palacky University, 17. Listopadu 12, 771 46 Olomouc, Czech Republic; viktor.krchnak@upol.cz; 2 Department of Chemistry and Biochemistry, University of Notre Dame, 251 Nieuwland Science Center, University of Notre Dame, Notre Dame, IN 46556, USA

**Keywords:** bicyclic compounds, heterocycles, iminiums, scaffold, solid-phase synthesis

## Abstract

We report two synthetic strategies for traceless solid-phase synthesis of molecular scaffolds comprising 6- to 8-membered rings fused with 5- to 7-membered rings. Traceless synthesis facilitated preparation of target molecules without any trace of polymer-supported linkers. The cyclization proceeded via acid-mediated tandem *N*-acylium ion formation followed by the nucleophilic addition of *O*- and *C*-nucleophiles. The presented synthetic strategy enabled, through the use of simple building blocks without any conformational preferences, the evaluation of the predisposition of different combinations of ring sizes to form fused ring molecular scaffolds. Compounds with any combination of [6,7 + 5,6,7] ring sizes were accessible with excellent crude purity. The 8-membered cyclic iminium was successfully fused only with the 5-membered cycle and larger fused ring systems were not formed, probably due to their instability.

## 1. Introduction

The enormous structural diversity of natural products comprising fused ring systems and, in particular, their wide range of biological activities, make them attractive synthetic targets. More than 60% of the drugs on the market have structures derived from natural origins [1,2]. Therefore, the search for pharmacologically relevant molecules that mimic natural products has become an integral part of drug discovery. Specific structural features of these molecular scaffolds are the presence of sp^3^ hybridized carbons and stereogenic centers [3]. Although these characteristics are typical of natural products, they are often missing in traditional compound collections for High Throughput Screening (HTS) [4].

Generally, the chemical space occupied by natural products overlays with biological space. Therefore, the exploitation of core structures derived from bioactive natural products has led to the design and implementation of biology-oriented synthesis (BIOS) [5,6] instead of synthesis focused only on the structural dissimilarity of compounds, i.e., diversity-oriented synthesis (DOS) [6,7].

Natural products comprising fused and bridged carbo- and heterocycles represent particularly intriguing molecular scaffolds with 3D architecture. Among the various chemical routes used to synthesize diverse nitrogenous heterocycles, tandem iminium ion cyclization-nucleophilic addition represents one of the most versatile strategies [8,9,10,11]. Typically, a cyclic iminium is formed from an amide nitrogen and an aldehyde attached to acyclic intermediate. Then, a cyclic *N*-acyliminium ion is transformed into fused or bridged structures by intramolecular nucleophilic addition [12,13]. Specifically, oxygen has previously served as an internal nucleophile in the preparation of five- [14,15], six- [16,17,18,19], seven- [13,20] and eight-membered lactam rings [13].

**Figure 1:**
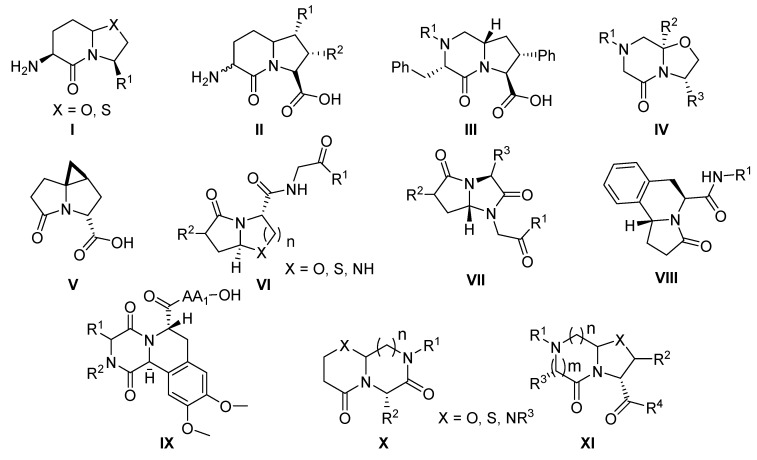
Reported synthesized bicyclic and tricyclic lactams.

Incorporation of fused lactam rings was also frequently applied for the synthesis of peptidomimetics [8,21]. The constrained structures were designed to mimic β-turns, the most frequent secondary structure found in proteins. Amino acid motifs constrained within a bicyclic lactam architecture have been applied to the design of modified peptides and peptidomimetics to discover new therapeutics and better understand the interactions of ligands with target enzymes and receptors. Compounds **I** [19,22] and **II** [23] (Figure 1) were designed as β-turn mimetics, and scaffold **III** [24] was synthesized as a conformationally constrained analogue for the Phe^7^-Phe^8^ region of substance P. The solution-phase synthesis of constrained dipeptide surrogates comprising [6 + 5]-fused lactams (structure **IV**) from 3-aza-1,5-diketoacids and amino alcohols was developed using a one-pot strategy [17]. Tricyclic constrained scaffold **V** was reported as a carbapenem mimetic [25]. Nielsen et al. synthesized a range of bicyclic dipeptide mimetics forming 5-hydroxylactam/*N*-acyliminium ion intermediates [15]. Depending on the amino acid side-chain, the cyclization occurs as side-chain cyclization **VI** if a nucleophilic moiety (X = O, S, NH, n = 1–3) is presented in the side-chain and as backbone cyclization **VII** in absence of heteroatomic moieties in the side-chain. Their studies on *N*-acyliminium Pictet-Spengler reactions [26] were extended to synthesize pyrroloisoquinolines **VIII** [27] or with an indole moiety and heterocycles such as furans and thiophenes to yield tri- and tetracyclic scaffolds [28]. Additionally *N*-carbamyliminium Pictet-Spengler reaction was used to form tetrahydro-β-carbolines and tetrahydroisoquinolines [29]. Synthesis of fused natural product-like diketopiperazines comprising different ring combination yielded molecular scaffolds **IX** [30].

Recently, our group reported a synthetic strategy enabling the incorporation of bicyclic scaffolds as peptide constraints during traditional peptide synthesis. The synthesis of bridged 6-oxa-3,8-diazabicyclo[3.2.1]octan-2-ones [31], fused tetrahydropyrazino[2,1-*b*][1,3]oxazine-4,7(6*H*,8*H*)-diones **X** (X = O, n = 1) [32], hexahydro-4*H*-pyrazino[1,2-*a*]pyrimidine-4,7(6*H*)-diones **X** (X = NR^3^, n = 1) [32] and hexahydropyrimido[1,2-*d*][1,4]diazepine-4,7(1*H*,6*H*)-diones **X** (X = NR^3^, n = 2) [33] were performed via westbound direction (towards the amino terminus) *N*-acyliminium cyclization–nucleophilic addition with Ser, Thr and 2,4-diaminobutyric acid as the sources of oxygen, sulfur and nitrogen nucleophiles, respectively. The solid-phase synthesis of tetrahydro-2*H*-oxazolo[3,2-a]pyrazine-5(3*H*)-ones **IV** (R^2^ = H) [34] was carried out via eastbound direction (towards the carboxyl terminus) cyclization, and the scope of this synthetic strategy was expanded recently to yield [7,8,9 + 5]-fused rings from Ser, Cys and C-nucleophiles (structure **XI**) [13].

In this report, we describe two synthetic strategies for traceless solid-phase synthesis of bicyclic scaffolds. Traceless synthesis provided access to compounds without any trace of a linker, a critical feature for preparation of compounds for structure-activity relationship (SAR). The synthesis was carried out in a modular fashion that allowed us to test any combination of potential ring sizes, with the first ring formed by a cyclic *N*-acyliminium species, and the second one fused by addition of oxygen and carbon nucleophiles, and to obtain [6,7,8 + 5,6,7]-fused ring systems.

## 2. Results

**Figure 2:**
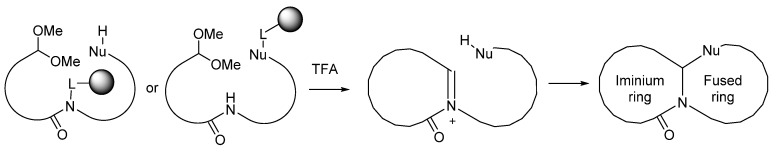
Two ways for immobilization of acyclic intermediates.

To prepare compounds on solid phase in a traceless manner we selected two potential anchoring functional groups that enabled immobilization of the first building blocks and polymer-supported synthesis of the acyclic precursor. The selected functional groups were not involved in any transformation during solid-phase synthesis, however, they participated in ring-closing reactions (C-N and C-O/C-C bond formations) in the final cleavage/cyclization step. The attachment to the resin was achieved via specific linkers that served as polymer-bound protecting group. Both strategies using either amide nitrogen or nucleophile for attachment to solid support were used (Figure 2, L stands for an acid-labile linker). Acid-mediated cleavage released compounds from the resin, demasked the aldehyde and triggered cyclic *N*-acyliminium formation followed by nucleophilic addition.

To perform the synthesis, we used simple and commercially available building blocks: amino alcohols (Fmoc-glycinol, Fmoc-β-alaninol and 4-(Fmoc-amino)butanol), α-bromocarboxylic acids (bromoacetic acid and (*S*)- and (*R*)-bromopropionic acids), protected amino aldehydes (containing one, two and three-carbon spacers) and sulfonyl chlorides or aryl fluorides. Because each ring is formed from different building blocks, this strategy allowed us to use an identical synthetic strategy to prepare any combination of different ring sizes in a truly combinatorial fashion. The size of the first ring, the cyclic *N*-acyliminium, can be controlled by different amino acids (α, β, γ) and by the length of the carbon spacer bearing the protected aldehyde. In this study, we focused on varying the length of the aldehyde spacer.

**Scheme 1:**
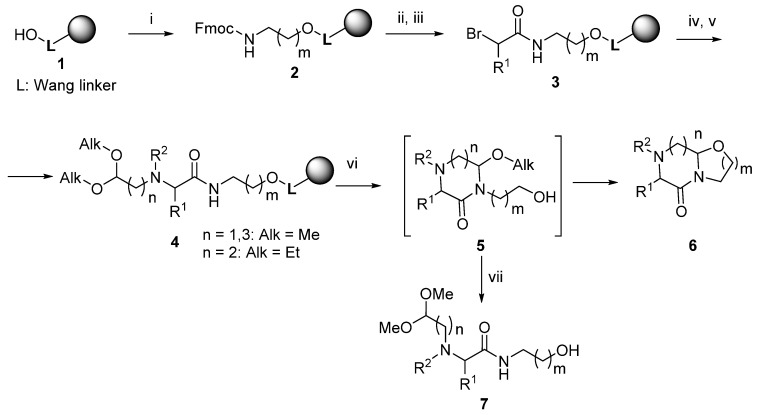
Polymer-supported synthesis of fused bicyclic compounds **6**. *Reagents and conditions*: (i) trichloroacetonitrile, anhydrous DCM, 0 °C, 30 min, DBU, DCM, rt, 1 h; resin washed and Fmoc-amino alcohol, BF_3_·Et_2_O, anhydrous THF added, rt, 30 min; (ii) 50% piperidine/DMF, rt, 15 min; (iii) α-bromocarboxylic acid, DIC, DCM, 5 min, then DIU filtered, DIEA added and the solution transferred to the reaction vessel with resin, rt, 1 h; (iv) amino-aldehyde dialkyl acetal, DIEA, DMF, rt, 2 h; (v) Ns-Cl or Tos-Cl, DIEA, DCM, rt, 2 h or 4-fluoro-3-nitrobenzotrifluoride, DIEA, DMSO, rt, overnight; (vi) TFA/DCM 1:1, rt, 1 h; (vii) MeOH, rt, overnight.

The synthesis of resin-bound acyclic precursors was accomplished using well-documented transformations. Briefly, the synthesis began with attachment via the oxygen atom of Fmoc-protected amino alcohols to Wang resin **1** by using trichloroacetimidate activation [35] to yield resin **2** (Scheme 1). The Fmoc group was cleaved by piperidine, and the polymer-supported amines were acylated with α-bromocarboxylic acids to afford resin **3**. Nucleophilic substitution of bromine with different protected amino aldehydes provided resin-bound secondary amines. To obtain resin **4**, the final derivatization was performed using 4-nitrobenzenesulfonyl chloride (Ns-Cl), 4-methylbenzene-sulfonyl chloride (Tos-Cl) or 4-fluoro-3-nitrobenzotrifluoride. TFA exposure of the polymer-supported acyclic precursors triggered their release from the resin, the removal of the aldehyde protecting group, and the formation of the six-, seven- and eight-membered *N*-acyliminium ions via hemiaminals **5**, followed by internal nucleophilic attack to provide target molecular scaffolds **6**. We typically used 50% TFA in DCM in the final step. However, the LC/MS analysis of crude compound **6**{*3*,*2*,*1*,*3*} revealed partial decomposition, i.e., the purity and yield of the crude products were only 38% and 16%, respectively, from the cleavage cocktail. We therefore used 10% TFA in DCM for cleavage followed by extraction of DCM/TFA with aqueous sodium bicarbonate. When the organic layer was separated and DCM evaporated under a stream of nitrogen, the purity substantially increased to 88%. Individual compounds **6** possess four points of diversification: *m* and *n* refer to the ring sizes and R^1^ and R^2^ are the substituents at carbon C^α^ and amine nitrogen, respectively (Table 1).

**Figure 3:**
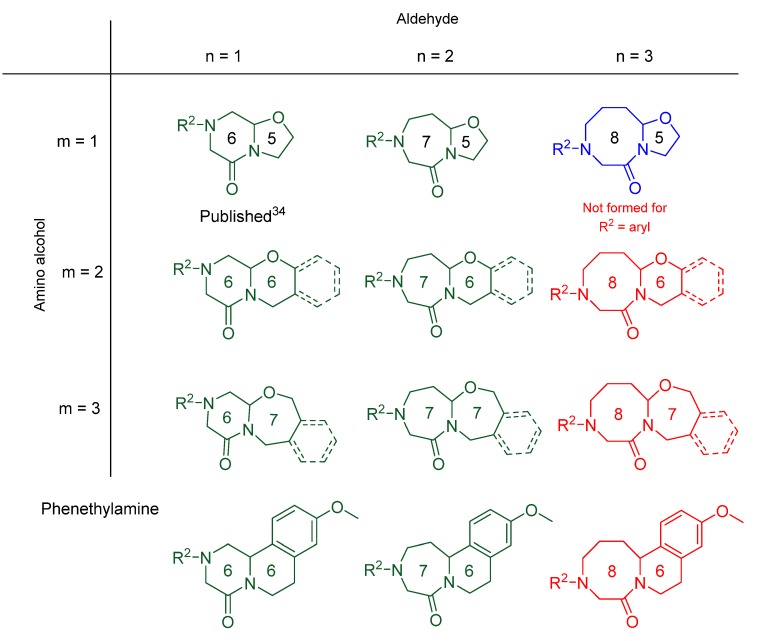
Color-coded structures of fused scaffolds. Green: good to excellent purity of product; blue: only specific combination was formed; red: no cyclic product detected.

Analysis of the results indicated that all combination of cyclic *N*-acyliminium 6- and 7-membered rings (prepared using protected aldehydes with one and two-carbon spacers, n = 1, 2) fused with 5-, 6- and 7-membered rings and provided the target molecular scaffolds with good to excellent purities and yields (Figure 3).

The protected aldehyde with a three-carbon spacer used for the synthesis of [8 + 5,6,7]-fused rings successfully cyclized only for [8 + 5] scaffolds derivatized as sulfonamides (**6**{*1*,*3*,*1*,*1*} and **6**{*1*,*3*,*1*,*2*}) not for those derivatized as aryl compounds. Attempts to prepare [8 + 6,7] fused rings were unsuccessful; LC/MS indicated the presence of a compound corresponding to cyclic hemiaminal **5**. To evaluate different cyclization procedures, we released hemiaminals **5**{*1*,*3*,*1*,*3*}, **5**{*2*,*3*,*1*,*3*} and **5**{3,*3*,*1*,*3*} from resins **4** using TFA/DCM 1:1. After the evaporation of TFA/DCM under a stream of nitrogen, the compounds were dissolved in methanol and left overnight at rt to yield **7**{*1*,*3*,*1*,*3*}, **7**{*2*,*3*,*1*,*3*} and **7**{*3*,*3*,*1*,*3*} in more than 90% crude purity. Compounds **7**{*1*,*3*,*1*,*3*} and **7**{*2*,*3*,*1*,*3*} were isolated, purified by reversed-phase HPLC and characterized by LCMS and NMR (Supplementary Materials). Then, we screened several Lewis acids, including stannous chloride dihydrate (SnCl_2_·2H_2_O) [36,37] and scandium triflate (Sc(OTf)_3_) [38] as well as different reaction conditions (temperature, concentration of Sc(OTf)_3_, reaction time from 1 to 10 days) and solvents (DCM, MeCN, dioxane) for the cyclization of **7**{*1*,*3*,*1*,*3*}, **7**{*2*,*3*,*1*,*3*} and **7**{*2*,*3*,*1*,*3*} in solution. Disappointingly, no cyclic product, not even a trace amount, was detected.

**Figure 4:**
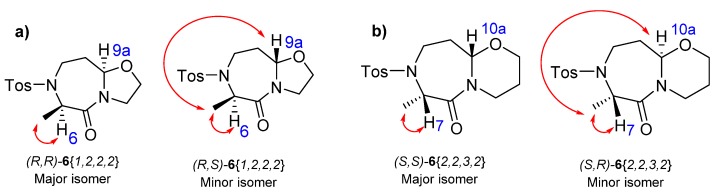
Configurational assignment of compounds (**a**) (*R*,*R*)- and (*R*,*S*)-**6**{*1*,*2*,*2*,*2*} and (**b**) (*S*,*S*)- and (*S*,*R*)-**6**{*2*,*2*,*2*,*2*}.

To address the stereoselectivity of this modular solid-phase synthesis, [7 + 5]-membered fused compound **6**{*1*,*2*,*2*,*2*} was synthesized using (*S*)-α-bromopropionic acid. The fused bicyclic compound featuring two asymmetric carbons was obtained as a mixture of diastereoisomers with a 5:1 ratio. Both diastereoisomers were isolated by reversed-phase HPLC, and their structures were confirmed from their 1D and 2D NMR spectra. The assignment of the configuration of the new stereogenic center in both diastereomers of fused bicycle **6**{*1*,*2*,*2*,*2*} was based on the diagnostic NOE effect observed in the NOESY 1D and ROESY 2D spectra of the compounds in deuterated DMSO. Whereas the NOE correlation between 6-H and CH_3_ was exhibited in both diastereoisomers, the NOE correlation between CH_3_ and 9a-H was exhibited only in the minor isomer and thus was decisive in confirming its configuration as (*R*,*R*)-**6**{*1*,*2*,*2*,*2*} and that of the major isomer as (*R*,*S*)-**6**{*1*,*2*,*2*,*2*} (Figure 4). Using the same experimental procedure and 1D and 2D NMR experiments, we identified the structures of the major and minor isomers of the enantiomeric compound afforded using (*R*)-α-bromopropionic acid as (*S*,*S*)-**6**{*1*,*2*,*3*,*2*} and (*S*,*R*)-**6**{*1*,*2*,*3*,*2*}, respectively. Both diastereoisomers were isolated and fully characterized (Table 2).

The reactions with (*R*)-α-bromopropionic acid provided [7 + 6]-fused ring model compounds **6***{2*,*2*,*3*,*2}* as a 55:45 mixture of diastereomers (Table 2). Both isomers were isolated by reversed-phase HPLC, and their structures were confirmed by 1D and 2D NMR spectroscopy. The NOESY experiments were performed in CDCl_3_ because of the overlap of the resonances of protons H6 and H10a in DMSO-*d_6_*. The results indicated that the major isomer prepared using (*R*)-α-bromopropionic acid was (*S*,*S*)-**6***{2*,*2*,*2*,*2}* and the minor isomer was (*S*,*R*)-**6***{2*,*2*,*2*,*2}* (Table 2).

NMR spectra of the compounds in CDCl_3_ provided interesting information regarding the stability of the diastereomers. We observed that the purified isomer (*S*,*S*)-**6***{2*,*2*,*3*,*2}* was not stable in CDCl_3_ solution and was partially converted into the (*S*,*R*)-**6***{2*,*2*,*3*,*2}* isomer at room temperature. The same effect was also observed for isomer (*S*,*R*)-**6***{2*,*2*,*3*,*2}*, which was converted into (*S*,*S*)-**6**{*2*,*2*,*3*,*2*}. After 8 days in CDCl_3_ solution, the two isomers (*S*,*S*)-**6***{2*,*2*,*3*,*2}* and (*S*,*R*)-**6***{2*,*2*,*3*,*2}* formed an equilibrium mixture of diastereomers with the same ratio (55:45), likely due to the opening of the acid-mediated fused ring and the formation of the cyclic iminium followed by ring closure. The same ratio of diastereomers was observed in the crude product after its TFA-mediated cleavage from the resin.

**Figure 5:**
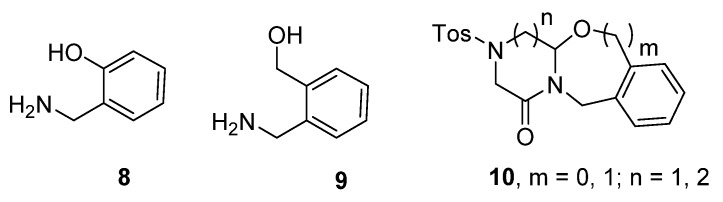
Compounds **10** prepared using the constrained amino alcohols **8** and **9** according to the Scheme 1.

The experimental results indicated that 8-membered cyclic *N*-acyliminiums formed fused systems with 5-membered rings but not with 6 and 7-membered rings. To address the effect of introducing a conformational constraint into the acyclic component (3-aminopropanol and 4-aminobutanol) on cyclization, we prepared resin-bound intermediates using 2-(aminomethyl)phenol (**8**) and (2-(aminomethyl)phenyl)methanol (**9**) (Figure 5). The beneficial effect of conformational constraints on cyclization was reported in unrelated cyclizations [39,40]. *N*-Fmoc derivatives of amino alcohols **8** and **9**, amino aldehydes with three different carbon spacers, bromoacetic acid and Tos-Cl were used for the synthesis of model compounds **10** according to Scheme 1. Cleaving compounds **10**{*1*,*1*} and **10**{*1*,*2*} was accomplished using a mild cleavage cocktail of 10% TFA in DCM. For compounds **10**{*2*,*1*} and **10**{*2*,*2*}, the typical cleavage cocktail (50% TFA in DCM) was employed. 

The results indicated that good to excellent purities and yields of **10** were successfully obtained via traceless solid-phase synthesis of combinations of cyclic *N*-acyliminium 6- and 7-membered rings (prepared from protected aldehydes with one and two-carbon spacers, n = 1, 2) with 6- and 7-membered rings (Table 3). Neither of the constrained amino alcohol promoted cyclization to form [8 + 6]- or [8 + 7]-fused rings.

**Scheme 2:**
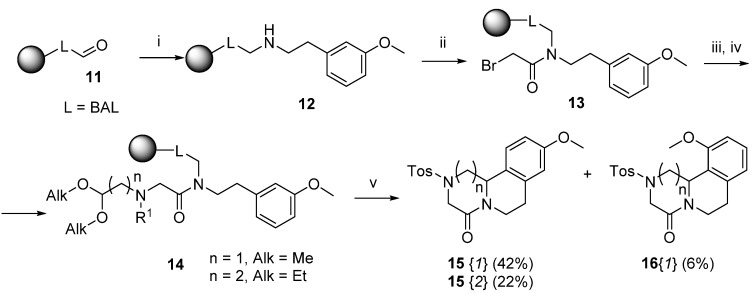
Polymer-supported synthesis of fused bicyclic compounds **15** and **16**. *Reagents and conditions*: (i) 0.5 M 2-(3-methoxyphenyl)ethan-1-amine in 10% AcOH/DMF, rt, overnight, then NaBH(AcO)_3_, rt, 5 h, then a second portion of NaBH(AcO)_3_, rt, 3 h; (ii) bromoacetic acid, DIC, DCM, rt, 1 h; (iii) amino-aldehyde dialkyl acetal, DIEA, DMF, rt, 2 h; (iv) Tos-Cl, DIEA, DCM, rt, 2 h; (v) TFA/DCM (1:1), rt, 1 h.

To demonstrate the second traceless strategy of the synthetic route for molecular scaffolds, we synthesized model compounds containing aromatic C-nucleophiles to effect the fused ring closure. In the previous synthesis, we used oxygen nucleophiles from amino alcohols for immobilization, a scenario that is not applicable for C-nucleophiles. To change our synthetic strategy, we attached the acyclic precursors to the resin via amide nitrogen. This strategy also allowed for the traceless synthesis of the target compounds. As shown in Scheme 2, synthesis was carried out on aminomethyl polystyrene resin **11** equipped with an acid-labile BAL (4-(4-formyl-3-methoxyphenoxy)butyric acid) linker [41]. 

After immobilization of the primary amines via reductive amination using 2-(3-methoxyphenyl)ethan-1-amine, secondary amines resin **12** was acylated with bromopropionic acid to give resin **13**. Then, the reaction with the amino-aldehyde dialkyl acetal, followed by *N*-derivatization with Tos-Cl, were carried out to yield intermediates **14**. Finally, TFA exposure triggered the release of the polymer-supported acyclic precursors from the resin, unmasking the protecting groups and forming the six- and seven-membered *N*-acyliminium ions. Internal nucleophilic attack then provided target molecules **15***{1}* and isomeric **16***{1}* or, in the case of the protected aldehyde with the n = 2 carbon spacer, only compound **15***{2}* (Table 4). These compounds were isolated by reversed-phase HPLC, and NMR spectra confirmed their structures. Disappointingly, formation of the [8 + 6] combination was not detected.

## 3. Materials and Methods

### 3.1. General

The solid-phase syntheses were carried out in plastic reaction vessels (syringes equipped with porous discs) using a manually operated synthesizer [42]. The volume of the wash solvent was 10 mL per 1 g of resin. The resin slurry was washed by shaking it with fresh solvent for at least 1 min before the solvent was changed. All reactions were carried out at ambient temperature unless stated otherwise. Commercially available Wang (100–200 mesh, 1.0 mmol/g) and aminomethyl resins (100-200 mesh, 1.26 mmol/g) were used. Commercial chemicals and solvents were ACS reagent grade. The yields of the crude products were calculated with respect to the loading of the first building block.

### 3.2. Reaction of Wang Resin ***1*** with Trichloroacetonitrile and Fmoc-amino Alcohol (Resin ***2***)

Wang resin **1** (1 mmol/g loading, 1 g) was suspended in 10 mL of anhydrous DCM, then 1.5 mL of trichloroacetonitrile was added, and the resin was left in a freezer for 30 min. Next, a solution of 100 µL of DBU in 2 mL of anhydrous DCM was added, and the slurry was shaken for 1 h. The resin was washed with anhydrous DCM (3×) and anhydrous THF (3×). The solution of Fmoc-amino alcohol (3 mmol) in 10 mL of anhydrous THF was added to the resin, followed by the dropwise addition of 63 µL of a solution of BF_3_·Et_2_O, and the slurry was shaken for 30 min. Resin **2** was washed THF (3×), MeOH (3×), and DCM (5×). A sample of the resin was washed with MeOH (3×) and dried, and 10 mg was cleaved with 50% TFA for 30 min, and the product quantified (Fmoc absorbance at 300 nm). Typical loading was between 0.35–0.50 mmol/g.

### 3.3. Acylation with α-Bromocarboxylic Acids (Resins ***3*** And ***13***)

Resin **2** (1 g) was washed with DCM and DMF, Fmoc was deprotected with 50% piperidine in DMF for 15 min, and the resin was washed with DMF (3×) and DCM (5×). Reaction of the resin with a solution of α-bromocarboxylic acid (5 mmol) in 10 mL of DCM was carried out in a syringe with a frit, and DIC (2.5 mmol, 386 µL) was added. After 5 min, DIU was filtered, and the solution was transferred to the syringe with resin **1** and shaken for 1 h. When bromoacetic acid was used, DIEA (2.5 mmol, 436 µL) was also added. Then, the resin was washed with DCM (3×).

### 3.4. Reaction with Amino-aldehyde Dialkyl Acetal and N-Derivatization (Resin ***4***)

Resin **3** (1 g) was washed with DMF (3×), a solution of amino-aldehyde dialkyl acetal (10 mmol) and DIEA (10 mmol, 1.74 mL) in 10 mL of DMF was added, and the slurry was shaken for 2 h. An analytical sample was taken for analysis, reacted with 0.5 M Fmoc-OSu in DCM for 1 h and cleaved with 50% TFA for 30 min.

Resin from the previous step was split into four 250 mg portions, which were washed with reaction solvent: DCM for sulfonamides (Ns-Cl, Tos-Cl) (1 mmol) and DMSO for 4-fluoro-3-nitrobenzotrifluoride (1 mmol). Then, 3 mL of the reaction solvent and DIEA (1.8 mmol, 174 µL) was added, and the slurry was shaken for 2 h (in the case of sulfonamides) or overnight (in the case of 4-trifluoromethyl-2-nitroaryl). Resin **4** was washed with the reaction solvent (3×) and then DCM (3×). A sample for analysis was reacted with 0.5 M Fmoc-OSu in DCM for 1 h and cleaved with 50% TFA for 30 min.

### 3.5. Acid-Mediated Cleavage, Cyclization and Isolation (Compounds ***6*** and ***7***) 

Resin **4** was treated with 50% TFA in DCM for 1 h. The TFA solution was collected, the resin was washed with 50% TFA in DCM (3×), and the extracts were combined and evaporated under a stream of nitrogen. When TFA exposure yielded cyclic hemiaminals (**5**), the oily residues were dissolved in MeOH and left at rt overnight to yield **7**. Compounds **6** and **7** were dissolved in acetonitrile or methanol and purified by semi-preparative reversed-phase HPLC.

### 3.6. Reductive Amination of the BAL Resin (Resin ***12***)

A 20 mL syringe was charged with BAL resin **11** (500 mg) and washed with anhydrous DMF (3×). Then, 0.5 M 2-(3-methoxyphenyl)ethan-1-amine and 10% AcOH in anhydrous DMF (5 mL) was added into the syringe and shaken overnight. Next, sodium triacetoxyborohydride NaBH(AcO)_3_ (2.5 mmol, 528 mg) was added to the syringe. Because of the evolution of hydrogen gas, the reaction vessel was vented to relieve pressure by piercing it on the top with a needle on the top, placing it in a horizontal position and shaking it for 5 h. A second portion of NaBH(AcO)_3_ (2.5 mmol, 528 mg) was added and shaken for 3 h. After the reaction, the resin was washed with 5% AcOH in DMF (3×) and DMF (3×), neutralized with 5% piperidine in DMF (10 mL) and washed with DMF (5×) and DCM (5×) to yield resin **12**. 

### 3.7. Reaction with Amino-aldehyde Dialkyl Acetal and N-Derivatization (Resin ***14***) Acid-mediated Cleavage, Cyclization and Isolation (Compounds ***15*** and ***16***)

The protocol was the same as that described for the synthesis of compound **6**.

## 4. Conclusions

We developed two synthetic strategies for traceless polymer-supported synthesis of natural product-like molecular scaffolds comprising two fused rings. The linear precursors were cyclized via acid-mediated tandem *N*-acylium ion formation followed by nucleophilic addition of O- and C-nucleophiles. Synthetic scheme facilitated the preparation of any combination of acyclic ring precursors for the synthesis of different ring sizes and assessed ring-forming limitations with respect to ring sizes. [6,7 + 5,6,7]-Fused cyclic combinations were obtained with excellent crude purity and yield. The 8-membered cyclic iminium was successfully fused only with the 5-membered cycle, whereas larger fused rings were not formed. The syntheses proceeded under mild conditions and used commercially available building blocks. Further work exploring the potential applicability of this strategy to larger ring sizes by using building blocks with the predisposition to adopt conformations favorable for large ring closure is in progress.

## Figures and Tables

**Table 1:** Synthesized fused heterocyclic compounds **6**.

Entry *	Compound	m	n	R^1^	R^2^	Rings	Purity [%] ^a^	Yield [%] ^b^
1	**6**{*1*,*2*,*1*,*1*}	1	2	H	Ns	7 + 5	94	70
2	**6**{*1*,*2*,*1*,*2*}	1	2	H	Tos	7 + 5	93	70
3	**6**{*1*,*2*,*1*,*3*}	1	2	H	2-NO_2_-4-CF_3_-C_6_H_3_	7 + 5	68	64
4	**6**{*1*,*3*,*1*,*1*}	1	3	H	Ns	8 + 5	86	66
5	**6**{*1*,*3*,*1*,*2*}	1	3	H	Tos	8 + 5	53	21
6	**6**{*2*,*1*,*1*,*3*}	2	1	H	2-NO_2_-4-CF_3_-C_6_H_3_	6 + 6	97	62
7	**6**{*2*,*2*,*1*,*1*}	2	2	H	Ns	7 + 6	98	83
8	**6**{*2*,*2*,*1*,*2*}	2	2	H	Tos	7 + 6	97	90
9	**6**{*2*,*2*,*1*,*3*}	2	2	H	2-NO_2_-4-CF_3_-C_6_H_3_	7 + 6	95	80
10	**6**{*3*,*1*,*1*,*3*}	3	1	H	2-NO_2_-4-CF_3_-C_6_H_3_	6 + 7	86	84
11	**6**{*3*,*2*,*1*,*2*}	3	2	H	Tos	7 + 7	72	54
12	**6**{*3*,*2*,*1*,*3*}	3	2	H	2-NO_2_-4-CF_3_-C_6_H_3_	7 + 7	88	44

* Entry 6 has previously been reported [34]. ^a^ The overall purity of crude final compounds **6** after a five-step synthesis. ^b^ Yield of the HPLC-purified compounds.

**Table 2:** Synthesized chiral fused heterocycles **6**.

Entry	Compound	m	n	R^1^	Rings	Purity [%] ^a^	Yield [%] ^b^
1	(*R*,*R*)-**6**{*1*,*2*,*2*,*2*}	1	2	(*R*)-Me	7 + 5	85	49
2	(*R*,*S*)-**6**{*1*,*2*,*2*,*2*}	1	2	(*R*)-Me	7 + 5	15	6
3	(*S*,*S*)-**6**{*1*,*2*,*3*,*2*}	1	2	(*S*)-Me	7 + 5	85	44
4	(*S*,*R*)-**6**{*1*,*2*,*3*,*2*}	1	2	(*S*)-Me	7 + 5	15	8
5	(*S*,*S*)-**6**{*2*,*2*,*3*,*2*}	2	2	(*S*)-Me	7 + 6	55	32
6	(*S*,*R*)-**6**{*2*,*2*,*3*,*2*}	2	2	(*S*)-Me	7 + 6	45	17

^a^ The overall purity of crude final compounds (*R* and *S*)-**6** after a five-step synthesis; ^b^ Yield of the HPLC-purified compounds.

**Table 3:** The synthesized fused heterocyclic compounds **10**.

Entry	Compound	m	n	Purity [%] ^a^	Yield [%] ^b^
1	**10**{*1*,*1*}	0	1	76	41
2	**10**{*1*,*2*}	0	2	81	43
3	**10**{*2*,*1*}	1	1	97	90
4	**10**{*2*,*2*}	1	2	96	81

^a^ The overall purity of the crude final compounds **10** after a five-step synthesis process; ^b^ Yield of the HPLC-purified compounds.

**Table 4:** Synthesis of fused heterocyclic compounds **19** and **20**.

**Entry**	**Compound**	**n**	**Purity [%] ^a^**	**Yield [%] ^b^**
1	**15**{*1*}	1	83	42
2	**16**{*1*}	1	17	6
3	**15**{*2*}	2	77	22

^a^ The overall purity of the crude products after a five-step synthesis. ^b^ Yield of the HPLC-purified compounds.

## Supplementary Materials

The following are available online. ^1^H, ^13^C NMR spectral data and figures of all compounds.
