# Supplementary material for: Traceless Solid-Phase Synthesis of [6,7,8 + 5,6,7]-Fused Molecular Frameworks
Source: Molecules. 2018 May 4;23(5):1090. doi: 10.3390/molecules23051090 (PMC6102595; doi:10.3390/molecules23051090)

Supporting Information

Traceless Solid-Phase Synthesis of [6,7,8 + 5,6,7]-Fused Molecular Frameworks

Vanesa Giménez-Navarro[a] and Viktor Krchňák[ab]*

[a]Department of Organic Chemistry, Institute of Molecular and Translational Medicine, Faculty of Science, Palacky University, 17. Listopadu 12, 771 46 Olomouc, Czech Republic

[b]Department of Chemistry and Biochemistry, University of Notre Dame, 251 Nieuwland Science Center, Notre Dame, Indiana 46556, United States.

*Corresponding author. E-mail: vkrchnak@nd.edu.

Table of Contents

1. General Information ………………………………………………………………….. S2

2. Analytical Data of Individual Compounds…………………………………………...… S4

3. ^{1}H and ^{13}C NMR spectra ..…………………………………………………………..…… S17

1. General Information

Material and Methods

Solvents were used without further purification. The commercially available Wang resin (100-200 mesh, 1% DVB, 1.0 mmol/g) was used. Synthesis was carried out on Domino Blocks (www.torviq.com) in disposable polypropylene reaction vessels.

The volume of wash solvent was 10 mL per 1 g of resin. For washing, resin slurry was shaken with the fresh solvent for at least 1 minute before changing the solvent. After adding a reagent solution, the resin slurry was manually vigorously shaken to break any potential resin clumps. Resin-bound intermediates were dried by a stream of nitrogen for prolonged storage and/or quantitative analysis.

For the LC/MS analysis a sample of resin (~5 mg) was treated by 50% TFA in DCM, the cleavage cocktail was evaporated by a stream of nitrogen, and cleaved compounds extracted into 1 mL of MeOH. The LC/MS analyses were carried out using two instruments. The first one is a Waters AcQuity UPLC H class instrument, that comprised a XSelect® HSS T3, 3.0 x 50 mm C18 reverse phase XP column, 2.5 µm particles. Mobile phases: 10 mM ammonium acetate in HPLC grade water (A) and HPLC grade acetonitrile (B). A gradient was formed from 20% to 80% of B in 6 minutes, flow rate of 0.6 mL/min. The MS electrospray source operated at capillary voltage 0.8 kV for a QDA detector and a desolvation temperature of 600 ^{o}C. The second is a Thermo Electron corporation and Scientific instrument, which comprised the XSelect® HSS T3, 3.0 x 50 mm C18 reverse phase XP column, 2.5 µm particles. Mobile phases: 10 mM ammonium acetate in HPLC grade water (A) and HPLC grade acetonitrile (B). A gradient was formed from 20% to 80% of B in 3 minutes; kept for 1 minute, flow rate of 0.8 mL/min. The column was re-equilibrated with 20% solution B for 1 minute. The APCI source operated at discharge current of 5 µA, vaporizer temperature of 400°C and capillary temperature of 250°C.

Purification was carried out on C18 reverse phase column 19 x 100 mm, 5 µm, gradient was formed from 10 mM aqueous ammonium acetate and acetonitrile, flow rate 15 mL/min.

^{1}H and ^{13}C NMR spectra were recorded at 400 MHz at ambient temperature (21 ^{o}C) in DMSO-d_6 solutions and referenced to the resonance signal of DMSO (^{1}H δ = 2.50 ppm, ^{13}C δ = 39.50 ppm). Both ^{1}H and ^{13}C chemical shifts, δ (in ppm) are relative to TMS; the interaction constants (*J*) are given in Hz.

2. Analytical data of individual compounds

7-((4-Nitrophenyl)sulfonyl)hexahydrooxazolo[3,2-*d*][1,4]diazepin-5(6*H*)-one 6{*1,2,1,1*}

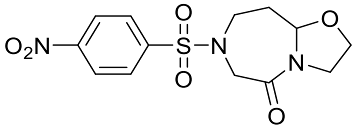

Yield 15.6 mg (70%) of amorphous solid. ^{1}H NMR (400 MHz, DMSO-d_6) δ = 8.49 - 8.35 (m, 2 H), 8.15 - 8.00 (m, 2 H), 5.01 (d, *J* = 9.6 Hz, 1 H), 4.03 (s, 2 H), 4.02 (dd, *J* = 15.7, 2.9 Hz, 1 H), 3.75 - 3.66 (m, 1 H), 3.60 - 3.51 (m, 1 H), 3.50 - 3.45 (m, 1 H), 3.45 - 3.40 (m, 1 H), 3.30 - 3.22 (m, 1 H), 2.19 - 2.11 (m, 1 H), 1.82 - 1.69 (m, 1 H). ^{13}C NMR (101 MHz, DMSO-d_6) δ = 165.2, 150.0, 143.6, 128.6, 124.7, 87.3, 64.5, 51.6, 45.3, 44.5, 32.5. HRMS (ESI-TOF): *m/z* calcd for $C_{13}H_{16}N_3O_6S$ $[M+H]^+$ 342.0754, found 342.0753.

7-Tosylhexahydrooxazolo[3,2-*d*][1,4]diazepin-5(6*H*)-one 6{*1,2,1,2*}

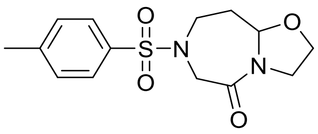

Yield 11.4 mg (70%) of amorphous solid. ^{1}H NMR (400 MHz, DMSO-d_6) δ = 7.70 - 7.64 (m, 2 H), 7.46 - 7.39 (m, 2 H), 4.95 (d, *J* = 9.1 Hz, 1 H), 4.02 (ddd, *J* = 8.7, 7.1, 2.9 Hz, 1 H), 3.95 (d, *J* = 16.5 Hz, 1 H), 3.90 (d, *J* = 16.5 Hz, 1 H), 3.69 (td, *J* = 8.9, 6.7 Hz, 1 H), 3.48 - 3.40 (m, 2 H), 3.37 - 3.33 (m, 1 H), 3.30 - 3.23 (m, 1 H), 2.40 (s, 3 H), 2.15 - 2.08 (m, 1 H), 1.79 - 1.65 (m, 1 H). ^{13}C NMR (101 MHz, DMSO-d_6) δ = 166.0, 144.2, 135.7, 130.5, 127.5, 87.8, 65.0, 52.2, 45.7, 45.1, 32.9, 21.5. HRMS (ESI-TOF): *m/z* calcd for $C_{14}H_{19}N_2O_4S$ $[M+H]^+$ 311.1060, found 311.1063.

7-(2-Nitro-4-(trifluoromethyl)phenyl)hexahydrooxazolo[3,2-*d*][1,4]diazepin-5(6*H*)-one 6{*1,2,1,3*}

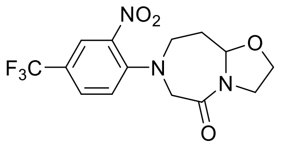

Yield 11.8 mg (64%) of amorphous solid. ^{1}H NMR (400 MHz, DMSO-d_6) δ = 8.13 (dd, *J* = 2.2, 0.7 Hz, 1 H), 7.91 - 7.85 (m, 1 H), 7.12 (d, *J* = 8.8 Hz, 1 H), 4.81 (dd, *J* = 9.1, 1.3 Hz, 1 H), 4.39 (d, *J* = 17.6 Hz, 1 H), 4.10 (ddd, *J* = 8.6, 7.6, 2.4 Hz, 1 H), 3.90 (d, *J* = 17.5 Hz, 1 H), 3.72 (td, *J* = 9.2, 7.1 Hz, 1 H), 3.57 (td, *J* = 10.2, 7.6 Hz, 1 H), 3.48 - 3.37 (m, 2 H), 3.08 (td, *J* = 12.2, 6.0 Hz, 1 H), 2.31 - 2.17 (m, 2 H). ^{13}C NMR (101 MHz, DMSO-d_6) δ = 167.6, 147.4, 137.8, 130.8 (q, *J* = 3.1 Hz), 124.7 (q, *J* = 4.3 Hz), 124.1(q, *J* = 271.0 Hz), 119.9, 118.8 (q, *J* = 33.7 Hz), 87.6, 64.6, 55.2, 49.3, 45.1, 32.3. HRMS (ESI-TOF): *m/z* calcd for $C_{14}H_{15}F_3N_3O$ $[M+H]^+$ 346.1009, found 346.1009.

(6*R*,9*aR*-6-Methyl-7-tosylhexahydrooxazolo[3,2-*d*][1,4]diazepin-5(6*H*)-one (*R,R*)-6{*1,2,2,2*}

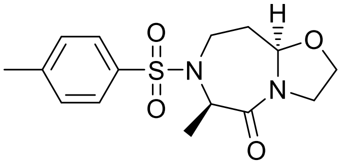

Yield 9.0 mg (49%) of amorphous solid. ^{1}H NMR (400 MHz, DMSO-d_6) δ = 7.72 - 7.67 (m, 2 H), 7.46 - 7.41 (m, 2 H), 4.85 (d, *J* = 9.7 Hz, 1 H), 4.58 (d, *J* = 7.3 Hz, 1 H), 4.13 - 4.04 (m, 1 H), 3.87 - 3.76 (m, 1 H), 3.73 - 3.64 (m, 1 H), 3.47 - 3.35 (m, 2 H), 3.17 (d, *J* = 4.0 Hz, 1 H), 2.40 (s, 3 H), 2.37 - 2.28 (m, 1 H), 1.80 (br. s., 1 H), 1.10 (d, *J* = 7.5 Hz, 3 H). ^{13}C NMR (101 MHz, DMSO-d_6) δ = 169.4, 144.2, 136.7, 130.5, 127.2, 113.5, 87.2, 64.7, 55.9, 45.9, 32.3, 21.5, 15.0. HRMS (ESI-TOF): *m/z* calcd for $C_{15}H_{21}N_2O_4S$ $[M+H]^+$ 325.1217, found 325.1218.

(6*R*,9*aS*)-6-Methyl-7-tosylhexahydrooxazolo[3,2-*d*][1,4]diazepin-5(6*H*)-one (*R,S*)-6{*1,2,2,2*}

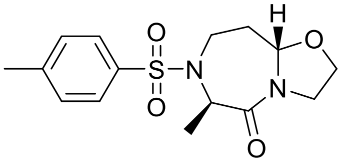

Yield 1.1 mg (6%) of amorphous solid.^{1}H NMR (400 MHz, DMSO-d_6) δ = 7.68 - 7.63 (m, 2 H), 7.43 - 7.39 (m, 2 H), 5.19 (dd, *J* = 10.2, 1.7 Hz, 1 H), 4.63 (q, *J* = 7.9 Hz, 1 H), 4.13 - 4.10 (m, 1 H), 3.95 (ddd, *J* = 8.7, 6.9, 3.0 Hz, 1 H), 3.85 (td, *J* = 15.5, 3.6 Hz, 1 H), 3.70 (td, *J* = 8.8, 6.6 Hz, 1 H), 3.58 - 3.52 (m, 1 H), 3.17 (d, *J* = 5.3 Hz, 2 H), 2.39 (s, 3 H), 1.52 - 1.39 (m, 1 H), 1.21 (d, *J* = 7.7 Hz, 3 H). ^{13}C NMR (101 MHz, DMSO-d_6) δ = 169.4, 144.2, 136.9, 130.5, 127.2, 113.5, 87.3, 64.7, 55.9, 45.9, 32.3, 21.5, 15.0. HRMS (ESI-TOF): *m/z* calcd for $C_{15}H_{21}N_2O_4S$ [M+H]$^+$ 325.1217, found 325.1218.

(6*S*,9*aS*)-6-Methyl-7-tosylhexahydrooxazolo[3,2-*d*][1,4]diazepin-5(6*H*)-one (*S,S*)-6{*1,2,3,2*}

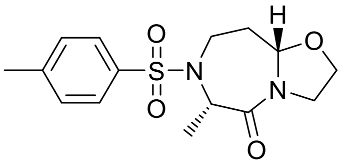

Yield 8.0 mg (44%) of amorphous solid. ^{1}H NMR (400 MHz, DMSO-d_6) δ = 7.72 - 7.68 (m, 2 H), 7.46 - 7.41 (m, 2 H), 4.85 (d, *J* = 9.3 Hz, 1 H), 4.58 (d, *J* = 7.8 Hz, 1 H), 4.13 - 4.04 (m, 1 H), 3.88 - 3.76 (m, 1 H), 3.74 - 3.63 (m, 1 H), 3.39 (d, *J* = 15.6 Hz, 1 H), 3.19 - 3.07 (m, 2 H), 2.40 (s, 3 H), 2.37 - 2.29 (m, 1 H), 1.87 - 1.73 (m, 1 H), 1.10 (d, *J* = 7.8 Hz, 3 H). ^{13}C NMR (101 MHz, DMSO-d_6) δ = 168.8, 143.7, 136.3, 130.0, 126.7, 86.7, 64.2, 55.4, 45.6, 45.4, 31.8, 21.0, 14.5. HRMS (ESI-TOF): *m/z* calcd for $C_{15}H_{21}N_2O_4S$ [M+H]$^+$ 325.1217, found 325.1217.

(6*S*,9*aR*)-6-Methyl-7-tosylhexahydrooxazolo[3,2-*d*][1,4]diazepin-5(6*H*)-one (*S,R*)-6{*1,2,3,2*}

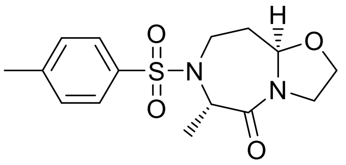

Yield 1.5 mg (8 %) of amorphous solid. ^{1}H NMR (400 MHz, DMSO-d_6) δ = 7.68 - 7.63 (m, 2 H), 7.43 - 7.38 (m, 2 H), 5.22 - 5.16 (m, 1 H), 4.66 - 4.59 (m, 1 H), 3.98 - 3.92 (m, 1 H), 3.85 (td, *J* = 15.4, 3.6 Hz, 1 H), 3.70 (td, *J* = 9.0, 6.1 Hz, 2 H), 3.58 - 3.51 (m, 1 H), 3.18 - 3.09 (m, 2 H), 2.39

(s, 3 H), 1.99 (d, *J* = 12.6 Hz, 1 H), 1.20 (s, 3 H). ^{13}C NMR (101 MHz, DMSO-d_6) δ = 169.4, 144.2, 143.1, 130.5, 127.2, 100.0, 87.2, 64.7, 55.7, 45.9, 32.3, 21.5, 15.0. HRMS (ESI-TOF): *m/z* calcd for $C_{15}H_{21}N_2O_4S$ [M+H]$^+$ 325.1217, found 325.1217.

7-((4-Nitrophenyl)sulfonyl)octahydro-5*H*-oxazolo[3,2-*d*][1,4]diazocin-5-one 6{*1,3,1,1*}

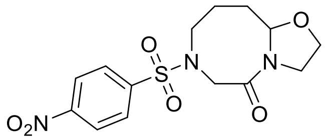

Yield 8.5 mg (66%) of amorphous solid. ^{1}H NMR (400 MHz, DMSO-d_6) δ = 8.45 - 8.39 (m, 2 H), 8.14 - 8.09 (m, 2 H), 6.17 (dd, *J* = 9.1, 4.2 Hz, 1 H), 4.32 (d, *J* = 18.0 Hz, 1 H), 3.93 (t, *J* = 6.6 Hz, 2 H), 3.73 - 3.63 (m, 2 H), 3.61 - 3.45 (m, 2 H), 2.81- 2.73 (m, 1 H), 1.94 - 1.69 (m, 3 H), 1.68 - 1.55 (m, 1 H). ^{13}C NMR (101 MHz, DMSO-d_6) δ = 164.8, 150.1, 142.6, 128.6, 124.8, 86.0, 62.0, 50.6, 48.9, 45.5, 32.3, 22.6. HRMS (ESI-TOF): *m/z* calcd for $C_{14}H_{18}N_3O_6S$ [M+H]$^+$ 356.0911, found 356.0909.

7-Tosyloctahydro-5*H*-oxazolo[3,2-*d*][1,4]diazocin-5-one 6{*1,3,1,2*}

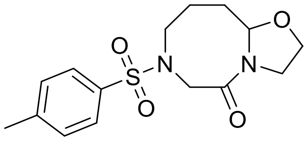

Yield 4.5 mg (21%) of amorphous solid. ^{1}H NMR (400 MHz, DMSO-d_6) δ = 7.72 - 7.67 (m, 2 H), 7.48 - 7.41 (m, 2 H), 6.25 - 6.19 (m, 1 H), 4.25 (d, *J* = 18.2 Hz, 1 H), 3.92 (t, *J* = 6.8 Hz, 2 H), 3.67 - 3.58 (m, 1 H), 3.57 - 3.45 (m, 3 H), 2.65 (td, *J* = 12.8, 3.5 Hz, 1 H), 2.40 (s, 3 H), 1.89 - 1.67 (m, 3 H), 1.64 - 1.53 (m, 1 H). ^{13}C NMR (101 MHz, DMSO-d_6) δ = 165.3, 143.8, 134.5, 130.1, 126.88, 85.9, 61.9, 50.7, 48.8, 45.5, 32.2, 22.6, 21.0. HRMS (ESI-TOF): *m/z* calcd for $C_{15}H_{21}N_2O_4S$ [M+H]$^+$ 325.1217, found 325.1217.

8-(2-Nitro-4-(trifluoromethyl)phenyl)hexahydro-2*H*,6*H*-pyrazino[2,1-*b*][1,3]oxazin-6-one 6{*2,1,1,3*}

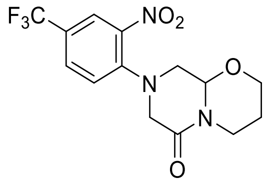

Yield 12.5 mg (62%) of amorphous solid. ^{1}H NMR (400 MHz, DMSO-d_6) δ = 8.47 - 8.39 (m, 1 H), 8.14 - 8.03 (m, 1 H), 4.82 (t, *J* = 4.1 Hz, 1 H), 4.38 - 4.28 (m, 1 H), 3.95 - 3.89 (m, 1 H), 3.86 (dd, *J* = 16.3, 1.3 Hz, 1 H), 3.72 (dd, *J* = 11.5, 3.3 Hz, 2 H), 3.70 (d, *J* = 16.3 Hz, 1 H), 3.52 (dd, *J* = 13.4, 4.3 Hz, 1 H), 3.41 (ddd, *J* = 13.4, 4.0, 1.2 Hz, 1 H), 2.76 (td, *J* = 12.5, 4.2 Hz, 1 H), 1.49 - 1.43 (m, 1 H), 1.43 - 1.33 (m, 1 H). ^{13}C NMR (101 MHz, DMSO-d_6) δ = 163.6, 145.7, 139.4, 130.3 (q, *J* = 3.4 Hz), 123.8 (q, *J* = 4.1 Hz), 123.5 (q, *J* = 271.0 Hz), 122.1, 120.4 (q, *J* = 33.7 Hz), 82.3, 66.6, 53.6, 51.24, 25.0. HRMS (ESI-TOF): *m/z* calcd for $C_{14}H_{15}F_3N_3O_4$ $[M+H]^+$ 346.1009, found 346.1008.

8-((4-Nitrophenyl)sulfonyl)hexahydro-2*H*-[1,3]oxazino[3,2-*d*][1,4]diazepin-6(7*H*)-one 6{*2,2,1,1*}

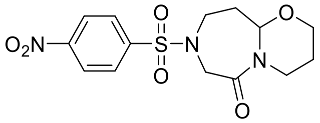

Yield 21.0 mg (83%) of amorphous solid. ^{1}H NMR (400 MHz, DMSO-d_6 δ = 8.45 - 8.38 (m, 2 H), 8.04 - 7.98 (m, 2 H), 4.65 (dd, *J* = 7.0, 2.3 Hz, 1 H), 4.22 (d, *J* = 17.4 Hz, 1 H), 4.17 (d, *J* = 17.4 Hz, 1 H), 4.14 - 4.09 (m, 1 H), 3.82 - 3.75 (m, 1 H), 3.62 (td, *J* = 11.7, 2.8 Hz, 1 H), 3.53 (ddd, *J* = 11.8, 9.3, 4.7 Hz, 1 H), 3.32 - 3.28 (m, 1 H), 2.63 (td, *J* = 12.7, 3.2 Hz, 1 H), 2.34 - 2.25 (m, 1 H), 2.23 - 2.13 (m, 1 H), 1.37 - 1.30 (m, 1 H), 1.18 - 1.05 (m, 1 H). ^{13}C NMR (101 MHz, DMSO-d_6) δ = 167.4, 150.4, 144.2, 129.2, 125.1, 85.9, 67.9, 49.6, 42.8, 41.5, 29.4, 25.5. HRMS (ESI-TOF): *m/z* calcd for $C_{14}H_{18}N_3O_6S$ $[M+H]^+$ 356.0911, found 356.0904.

8-Tosylhexahydro-2*H*-[1,3]oxazino[3,2-*d*][1,4]diazepin-6(7*H*)-one 6{*2,2,1,2*}

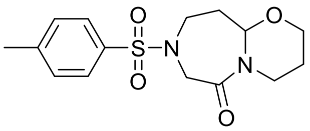

Yield 28.0 mg (90%) of amorphous solid. ^{1}H NMR (400 MHz, DMSO-d_6) δ = 7.64 - 7.60 (m, 2 H), 7.43 - 7.38 (m, 2 H), 4.62 (dd, *J* = 7.1, 2.4 Hz, 1 H), 4.18 - 4.11 (m, 1 H), 4.13 (d, *J* = 17.3 Hz, 1 H), 4.06 (d, *J* = 17.3 Hz, 1 H), 3.86 - 3.79 (m, 1 H), 3.62 (td, *J* = 11.6, 3.0 Hz, 1 H), 3.44 (ddd, *J* = 11.7, 8.7, 4.7 Hz, 1 H), 3.26 - 3.19 (m, 1 H), 2.63 (td, *J* = 12.6, 3.3 Hz, 1 H), 2.39 (s, 3 H), 2.29 - 2.20 (m, 1 H), 2.19 - 2.09 (m, 1 H), 1.41 - 1.32 (m, 1 H), 1.31 - 1.17 (m, 1 H). ^{13}C NMR (101 MHz, DMSO-d_6) δ = 167.5, 143.6, 135.4, 129.9, 127.2, 85.7, 67.4, 49.3, 42.4, 41.1, 29.3, 25.1, 21.1. HRMS (ESI-TOF): *m/z* calcd for $C_{15}H_{21}N_2O_4S$ $[M+H]^+$ 325.1217, found 325.1222.

8-(2-Nitro-4-(trifluoromethyl)phenyl)hexahydro-2*H*-[1,3]oxazino[3,2-*d*][1,4]diazepin-6(7*H*)-one 6{*2,2,1,3*}

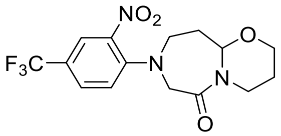

Yield 19.0 mg (80%) of amorphous solid. ^{1}H NMR (400 MHz, DMSO-d_6) δ = 8.12 - 8.07 (m, 1 H), 7.85 (dd, *J* = 9.0, 2.3 Hz, 1 H), 7.10 (d, *J* = 9.0 Hz, 1 H), 4.81 (d, *J* = 5.2 Hz, 1 H), 4.52 (d, *J* = 17.5 Hz, 1 H), 4.43 (m, 1 H), 4.11 (d, *J* = 17.5 Hz, 1 H), 3.83 - 3.76 (m, 1 H), 3.66 (td, *J* = 11.7, 2.7 Hz, 1 H), 3.30 - 3.23 (m, 1 H), 2.91 (td, *J* = 12.7, 3.1 Hz, 1 H), 2.80 (td, *J* = 11.2, 4.6 Hz, 1 H), 2.61 - 2.52 (m, 1 H), 2.31 - 2.21 (m, 1 H), 1.46 - 1.38 (m, 1 H), 1.38 - 1.25 (m, 1 H). ^{13}C NMR (101 MHz, DMSO-d_6) δ = 169.5, 146.9, 136.4, 129.9 (q, *J* = 3.4 Hz), 124.3 (q, *J* = 4.3 Hz), 123.7 (q, *J* = 271.0 Hz), 117.8, 117.2 (q, *J* = 33.7 Hz), 85.2, 67.8, 53.4, 46.1, 43.4, 29.7, 26.1. HRMS (ESI-TOF): *m/z* calcd for $C_{15}H_{17}F_3N_3O_4$ $[M+H]^+$ 360.1166, found 360.1171.

(7*S*,10a*S*)-7-Methyl-8-tosylhexahydro-2*H*-[1,3]oxazino[3,2-*d*][1,4]diazepin-6(7*H*)-one (*S,S*)-6{*2,2,3,2*}

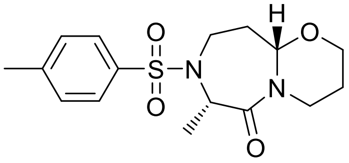

Yield 22.0 mg (32%) of amorphous solid. ^{1}H NMR (400 MHz, DMSO-d_6) δ = 7.67 - 7.63 (m, 2 H), 7.43 - 7.38 (m, 2 H), 4.72 - 4.63 (m, 2 H), 4.18 - 4.12 (m, 1 H), 3.88 - 3.73 (m, 2 H), 3.69 (td, *J* = 11.6, 3.1 Hz, 1 H), 3.22 - 3.14 (m, 1 H), 2.89 (td, *J* = 12.5, 3.3 Hz, 1 H), 2.38 (s, 3 H), 2.25 - 2.18 (m, 2 H), 1.39 - 1.32 (m, 1 H), 1.25 (d, *J* = 7.7 Hz, 3 H), 1.26 - 1.17 (m, 1 H). ^{13}C NMR (101 MHz, DMSO-d_6) δ = 170.8, 143.2, 136.8, 129.7, 126.9, 84.9, 67.7, 56.4, 44.2, 37.5, 29.1, 25.3, 21.0, 15.4. HRMS (ESI-TOF): *m/z* calcd for $C_{16}H_{23}N_2O_4S$ $[M+H]^+$ 339.1372, found 339.1371.

(7*S*,10*aR*)-7-Methyl-8-tosylhexahydro-2*H*-[1,3]oxazino[3,2-*d*][1,4]diazepin-6(7*H*)-one (*S,R*)-6{*2,2,3,2*}

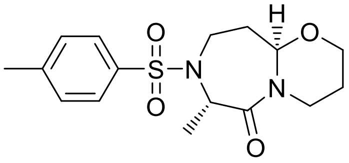

Yield 12.5 mg (17%) of amorphous solid. ^{1}H NMR (400 MHz, DMSO-d_6) δ = 7.67 - 7.63 (m, 2 H), 7.45 - 7.41 (m, 2 H), 4.64 - 4.56 (m, 2 H), 3.95 - 3.83 (m, 2 H), 3.80 - 3.71 (m, 1 H), 3.59 (ddd, *J* = 10.0, 10.9, 4.1 Hz, 1 H), 3.22 - 3.14 (m, 1 H), 2.83 - 2.75 (m, 1 H), 2.39 (s, 3 H), 2.11 - 2.07 (m, 1 H), 2.07 - 2.01 (m, 1 H), 1.68 - 1.55 (m, 1 H), 1.53 - 1.44 (m, 1 H), 1.22 (d, *J* = 7.5 Hz, 3 H). ^{13}C NMR (101 MHz, DMSO-d_6) δ = 170.8, 143.2, 136.8, 129.7, 126.9, 84.9, 67.7, 56.4, 44.2, 37.5, 29.1, 25.3, 21.0, 15.4. HRMS (ESI-TOF): *m/z* calcd for $C_{16}H_{23}N_2O_4S$ $[M+H]^+$ 339.1372, found 339.1371.

9-(2-Nitro-4-(trifluoromethyl)phenyl)octahydro-7*H*-pyrazino[2,1-*b*][1,3]oxazepin-7-one 6{*3,1,1,3*}

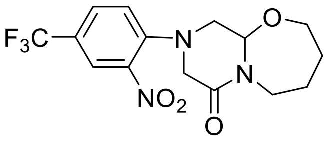

Yield 12.6 mg (84%) of amorphous solid. ^{1}H NMR (400 MHz, DMSO-d_6) δ = 8.17 (d, *J* = 2.2 Hz, 1 H), 7.86 (dd, *J* = 8.9, 2.3 Hz, 1 H), 7.53 (d, *J* = 8.9 Hz, 1 H), 5.09 (t, *J* = 4.2 Hz, 2 H), 3.98 - 3.89 (m, 1 H), 3.78 - 3.74 (m, 1 H), 3.68 - 3.55 (m, 3 H), 3.11 - 3.03 (m, 1 H), 1.77 - 1.63 (m, 4 H), 1.62 - 1.50 (m, 1 H). ^{13}C NMR (101 MHz, DMSO-d_6) δ = 165.2, 145.5, 138.5, 130.0 (q, *J* = 3.4 Hz), 123.8 (q, *J* = 4.3 Hz), 123.1 (q, *J* = 271.0 Hz), 121.4, 119.4 (q, *J* = 33.7 Hz), 82.4, 66.9, 52.9, 51.2, 44.4, 29.3, 24.8. HRMS (ESI-TOF): *m/z* calcd for $C_{15}H_{17}F_3N_3O_4$ $[M-H]^+$ 358.1009, found 358.0985.

9-Tosyloctahydro-[1,4]diazepino[7,1-*b*][1,3]oxazepin-7(8*H*)-one 6{*3,2,1,2*}

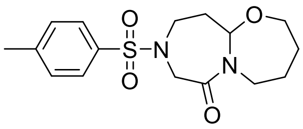

Yield 11.0 mg (54%) of amorphous solid. ^{1}H NMR (400 MHz, DMSO-d_6) δ = 7.67 - 7.61 (m, 2 H), 7.45 - 7.37 (m, 2 H), 4.99 (dd, *J* = 1.3, 8.0 Hz, 1 H), 4.02 - 3.95 (m, 1 H), 3.95 - 3.89 (m, 1 H), 3.84 (d, *J* = 13.0 Hz, 1 H), 3.59 - 3.45 (m, 2 H), 3.32 - 3.23 (m, 1 H), 3.11 - 3.01 (m, 2 H), 2.39 (s, 3 H), 1.85 - 1.77 (m, 1 H), 1.77 - 1.54 (m, 3 H), 1.52 - 1.33 (m, 2 H). ^{13}C NMR (101 MHz, DMSO-d_6) δ = 168.5, 144.1, 135.9, 130.4, 127.5, 87.5, 70.7, 53.3, 45.9, 42.7, 33.1, 28.3, 27.7, 21.5. HRMS (ESI-TOF): *m/z* calcd for $C_{16}H_{23}N_2O_4S$ $[M+H]^+$ 339.1373, found 339.1374.

9-(2-Nitro-4-(trifluoromethyl)phenyl)octahydro-[1,4]diazepino[7,1-*b*][1,3]oxazepin-7(8*H*)-one 6{*3,2,1,3*}

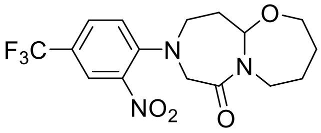

Yield 6.0 mg (44%) of amorphous solid. ^{1}H NMR (400 MHz, DMSO-d_6) δ = 8.10 (dd, *J* = 2.3, 0.8 Hz, 1 H), 7.85 (dd, *J* = 9.1, 2.3 Hz, 1 H), 7.07 (d, *J* = 8.8 Hz, 1 H), 4.89 (d, *J* = 7.3 Hz, 1 H), 4.44 (d, *J* = 17.1 Hz, 1 H), 4.06 (d, *J* = 17.6 Hz, 1 H), 4.05 - 3.97 (m, 1 H), 3.51 - 3.38 (m, 3 H), 3.06 (ddd, *J* = 13.5, 8.6, 4.4 Hz, 1 H), 2.89 (td, *J* = 11.4, 5.2 Hz, 1 H), 2.44 - 2.33 (m, 1 H), 2.26 - 2.15 (m, 1 H), 1.76 - 1.54 (m, 3 H), 1.35 - 1.21 (m, 1 H). ^{13}C NMR (101 MHz, DMSO-d_6) δ = 170.6, 147.0, 136.6, 130.0 (q, *J* = 3.4 Hz), 124.2 (q, *J* = 4.3 Hz), 123.7 (q, *J* = 271.0 Hz), 118.5, 117.2 (q, *J* = 34 Hz), 85.4, 68.2, 54.7, 47.1, 45.6, 31.0, 27.6, 25.7. HRMS (ESI-TOF): *m/z* calcd for $C_{16}H_{19}F_3N_3O_4$ $[M+H]^+$ 374.1322, found 374.1321.

2-((4,4-Dimethoxybutyl)(2-nitro-4-(trifluoromethyl)phenyl)amino)-*N*-(2-hydroxyethyl) acetamide 7{*1,3,1,3*}

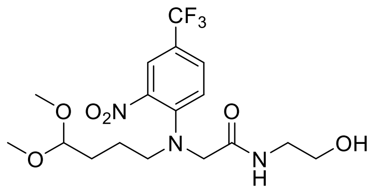

Yield 13.0 mg (42%) of amorphous solid. ^{1}H NMR (400 MHz, DMSO-d_6) δ = 8.06 (dd, *J* = 2.3, 0.8 Hz, 1 H), 7.99 (t, *J* = 5.6 Hz, 1 H), 7.78 (dd, *J* = 9.0, 2.4 Hz 1 H), 7.40 (d, *J* = 8.8 Hz, 1 H), 4.31 - 4.27 (m, 1 H), 3.85 (s, 2 H), 3.38 (t, *J* = 6.0 Hz, 2 H), 3.23 (t, *J* = 7.1 Hz, 2 H), 3.17 (s, 6 H), 3.11 (q, *J* = 6.0 Hz, 2 H), 1.88 (s, 1 H), 1.53 - 1,45 (m, 4 H). ^{13}C NMR (101 MHz, DMSO-d_6) δ = 167.9, 146.7, 138.9, 129.4 (q, *J* = 3.2 Hz), 123.6 (q, *J* = 4.1 Hz), 123.7(q, *J* = 271.0 Hz), 121.8, 118.3 (q, *J* = 33.7 Hz), 59.7, 54.7, 52.4, 52.0, 41.4, 29.1, 21.7. HRMS (ESI-TOF): *m/z* calcd for $C_{17}H_{24}F_3N_3NaO_6$ $[M+Na]^+$ 446.1509, found 446.1509.

2-((4,4-Dimethoxybutyl)(2-nitro-4-(trifluoromethyl)phenyl)amino)-*N*-(3-hydroxypropyl) acetamide 7{*2,3,1,3*}

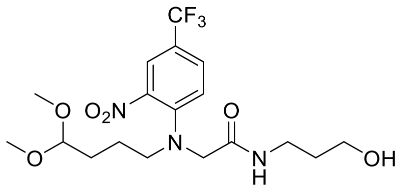

Yield 29.0 mg (88%) of amorphous solid. ^{1}H NMR (400 MHz, DMSO-d_6) δ = 8.06 (dd, *J* = 2.3, 0.7 Hz, 1 H), 7.95 (t, *J* = 5.6 Hz, 1 H), 7.81 - 7.75 (m, 1 H), 7.41 (d, *J* = 8.8 Hz, 1 H), 4.31 - 4.27 (m, 1 H), 3.83 (s, 2 H), 3.38 (t, *J* = 6.3 Hz, 2 H), 3.23 (t, *J* = 6.7 Hz, 2 H), 3.18 (s, 6 H), 3.13 - 3.06 (m, 2 H), 1.84 (s, 1 H), 1.57 - 1.43 (m, 6 H). ^{13}C NMR (101 MHz, DMSO-d_6) δ = 167.7, 146.7, 138.9, 129.4 (q, *J* = 3.2 Hz), 123.6 (q, *J* = 4.2 Hz), 123.7(q, *J* = 271.0 Hz), 121.8, 118.3 (q, *J* = 33.5 Hz), 103.7, 58.3, 54.7, 52.4, 52.1, 35.8, 32.2, 29.1, 22.1, 21.7. HRMS (ESI-TOF): *m/z* calcd for $C_{18}H_{26}F_3N_3NaO_6$ [M+Na]$^+$ 460.1666, found 460.1666.

2-Tosyl-1,2,3,11*a*-tetrahydro-4*H*,6*H*-benzo[e]pyrazino[2,1-*b*][1,3]oxazin-4-one 10{*1,1*}

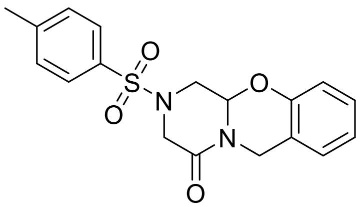

Yield 4.4 mg (41%) of amorphous solid. ^{1}H NMR (400 MHz, DMSO-d_6) δ = 7.76 - 7.71 (m, 2 H), 7.48 - 7.42 (m, 2 H), 7.21 - 7.15 (m, 2 H), 6.97 (td, *J* = 7.5, 0.9 Hz, 1 H), 6.89 - 6.85 (m, 1 H), 5.41 (t, *J* = 2.9 Hz, 1 H), 5.12 (d, *J* = 16.8 Hz, 1 H), 4.22 (d, *J* = 16.8 Hz, 1 H), 3.94 (dd, *J* = 16.2, 1.7 Hz, 1 H), 3.88 (td, *J* = 13.5, 2.1 Hz, 1 H), 3.52 (d, *J* = 16.2 Hz, 1 H), 2.39 (s, 3 H), 2.31 (d, *J* = 2.9 Hz, 1 H). ^{13}C NMR (101 MHz, DMSO-d_6) δ = 162.8, 152.7, 144.2, 132.3, 130.0, 127.9, 127.7, 127.2, 121.8, 119.4, 116.7, 79.7, 48.8, 46.1, 41.4, 21.0. HRMS (ESI-TOF): *m/z* calcd for $C_{18}H_{19}N_2O_4S$ [M+H]$^+$ 359.1060, found 359.1060.

3-Tosyl-3,4,5,5*a*-tetrahydro-11*H*-benzo[5,6][1,3]oxazino[3,2-*d*][1,4]diazepin-1(2*H*)-one 10{*1,2*}

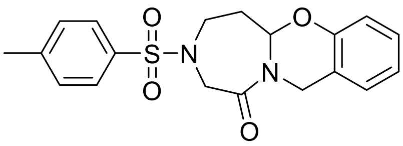

Yield 5.7 mg (43%) of amorphous solid. ^{1}H NMR (400 MHz, DMSO-d_6) δ = 7.52 - 7.48 (m, 2 H), 7.16 - 7.09 (m, 1 H), 7.08 - 7.05 (m, 3 H), 6.92 (td, *J* = 7.4, 1.1 Hz, 1 H), 6.70 (dd, *J* = 8.2 Hz, 1.0 Hz, 1 H), 5.21 - 5.19 (m, 1 H), 4.70 (d, *J* = 16.7 Hz, 1 H), 4.29 (d, *J* = 17.7 Hz, 1 H), 4.24 (d, *J* = 17.7 Hz, 1 H), 4.15 (d, *J* = 16.7 Hz, 1 H), 3.76 (td, *J* = 12.2, 4.5 Hz, 1 H), 3.26 - 3.19 (m, 1 H), 2.66 - 2.55 (m, 1 H), 2.44 - 2.33 (m, 1 H), 2.09 (s, 3 H). ^{13}C NMR (101 MHz, DMSO-d_6) δ = 166.6, 153.7, 143.6, 135.5, 129.9, 127.9, 127.4, 127.1, 121.8, 120.7, 117.1, 82.7, 49.6, 44.7, 40.6, 28.1, 21.4. HRMS (ESI-TOF): *m/z* calcd for $C_{19}H_{21}N_2O_4S$ $[M+H]^+$ 373.1217, found 373.1217.

2-Tosyl-1,2,3,6,11,12*a*-hexahydro-4*H*-benzo[*e*]pyrazino[2,1-*b*][1,3]oxazepin-4-one 10{*2,1*}

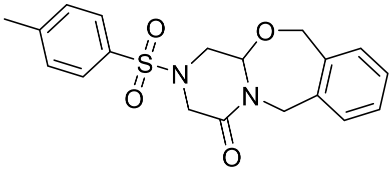

Yield 31.0 mg (90%) of amorphous solid. ^{1}H NMR (400 MHz, DMSO-d_6) δ = 7.69 - 7.64 (m, 2 H), 7.46 - 7.41 (m, 2 H), 7.32 - 7.21 (m, 4 H), 5.28 (t, *J* = 3.0 Hz, 1 H), 5.06 (d, *J* = 13.9 Hz, 1 H), 5.00 (d, *J* = 14.8 Hz, 1 H), 4.80 (d, *J* = 14.0 Hz, 1 H), 4.28 (d, *J* = 14.7 Hz, 1 H), 3.74 (dd, *J* = 16.2, 1.3 Hz, 1 H), 3.67 (dd, *J* = 12.5, 2.1 Hz, 1 H), 3.31 (d, *J* = 16.1 Hz, 1 H), 3.12 (dd, *J* = 12.9, 3.3 Hz, 1 H), 2.40 (s, 3 H). ^{13}C NMR (101 MHz, DMSO-d_6) δ = 163.8, 144.6, 139.2, 138.5, 132.5, 130.5, 129.4, 129.0, 128.2, 127.9, 88.1, 72.4, 49.6, 49.1, 48.2, 21.6. HRMS (ESI-TOF): *m/z* calcd for $C_{19}H_{21}N_2O_4S$ $[M+H]^+$ 373.1217, found 373.1217.

3-Tosyl-3,4,5,5a,7,12-hexahydrobenzo[*e*][1,4]diazepino[7,1-*b*][1,3]oxazepin-1(2*H*)-one 10*{2,2}*

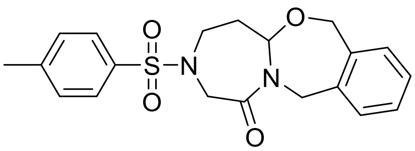

Yield 23.0 mg (81%) of amorphous solid. ^{1}H NMR (400 MHz, DMSO-d_6) δ = 7.63 - 7.58 (m, 2 H), 7.35 - 7.30 (m, 2 H), 7.60 - 7.13 (m, 4 H), 5.23 (dd, *J* = 6.8, 3.0 Hz, 1 H), 4.96 - 4.92 (m, 1 H), 4.91 (s, 1 H), 4.61 (d, *J* = 14.9 Hz, 1 H), 4.34 (d, *J* = 14.9 Hz, 1 H), 4.04 - 3.99 (m, 1 H), 3.98 - 3.93 (m, 1 H), 3.64 - 3.56 (m, 1 H), 3.19 - 3.11 (m, 1 H), 2.38 (s, 3 H), 2.10 - 2.16 (m, 2 H). ^{13}C NMR (101 MHz, DMSO-d_6) δ = 168.2, 144.0, 138.2, 137.7, 135.4, 130.3, 129.3, 127.6, 127.6, 127.4, 90.5, 73.2, 51.6, 48.6, 43.9, 31.3, 21.5. HRMS (ESI-TOF): *m/z* calcd for $C_{20}H_{23}N_2O_4S$ $[M+H]^+$ 387.1373, found 387.1373.

9-Methoxy-2-tosyl-1,2,3,6,7,11*b*-hexahydro-4*H*-pyrazino[2,1-*a*]isoquinolin-4-one 15{*1*}

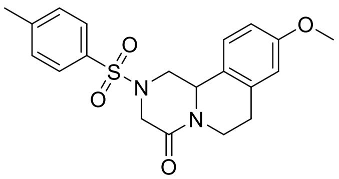

Yield 12.0 mg (42%) of amorphous solid. ^{1}H NMR (400 MHz, DMSO-d_6) δ = 7.77 - 7.71 (m, 2 H), 7.47 - 7.42 (m, 2 H), 7.26 (d, *J* = 8.6 Hz, 1 H), 6.82 (dd, *J* = 8,6, 2.7 Hz, 1 H), 6.73 (d, *J* = 2.7 Hz, 1 H), 4.83 (dd, *J* = 9.7, 4.5 Hz, 1 H), 4.43 (td, *J* = 12.3, 3.9 Hz, 1 H), 4.19 (ddd, *J* = 12.2, 4.6, 1.6 Hz, 1 H), 3.90 (dd, *J* = 16.1, 1.0 Hz, 1 H), 3.72 (s, 3 H), 3.32 (s, 1 H), 2.84 - 2.75 (m, 1 H), 2.74 - 2.64 (m, 3 H), 2.39 (s, 3 H). ^{13}C NMR (101 MHz, DMSO-d_6) δ = 162.5, 158.1, 144.2, 136.5, 131.9, 130.1, 127.7, 126.5, 125.2, 113.6, 112.7, 55.1, 54.0, 48.5, 48.3, 38.4, 28.1, 21.1. HRMS (ESI-TOF): *m/z* calcd for $C_{20}H_{23}N_2O_4S$ $[M+H]^+$ 387.1373, found 387.1373.

10-Methoxy-3-tosyl-1,3,4,7,8,12*b*-hexahydro-[1,4]diazepino[7,1-*a*]isoquinolin-5(2*H*)-one 15{*2*}

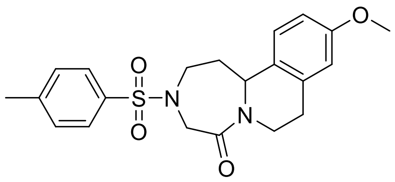

Yield 6.0 mg (22%) of amorphous solid. ^{1}H NMR (400 MHz, DMSO-d_6) δ = 7.71 - 7.67 (m, 2 H), 7.44 (d, *J* = 8.2 Hz, 2 H), 7.03 (d, *J* = 8.5 Hz, 1 H), 6.77 (dd, *J* = 8,5, 2.7 Hz, 1 H), 6.72 (d, *J* = 2.6 Hz, 1 H), 4.96 (dd, *J* = 7.9, 3.9 Hz, 1 H), 4.20 - 4.14 (m, 1 H), 4.14 - 4.07 (m, 1 H), 3.86 - 3.78 (m, 1 H), 3.71 (s, 3 H), 3.54 - 3.47 (m, 2 H), 3.30 - 3.24 (m, 1 H), 3.14 (ddd, *J* = 13.0, 9.7, 3.8 Hz, 1 H), 2.66 - 2.59 (m, 1 H), 2.41 (s, 3 H), 1.69 - 1.62 (m, 2 H). ^{13}C NMR (101 MHz, DMSO-d_6) δ = 168.7, 157.8, 143.6, 136.3, 135.5, 129.9, 129.2, 127.9, 127.2, 112.9, 112.5, 55.1, 52.7, 48.9, 40.3, 40.2, 37.0, 27.7, 29.9. HRMS (ESI-TOF): *m/z* calcd for $C_{21}H_{25}N_2O_4S$ [M+H]$^+$ 401.1530, found 401.1530.

11-Methoxy-2-tosyl-1,2,3,6,7,11*b*-hexahydro-4*H*-pyrazino[2,1-*a*]isoquinolin-4-one 16*{1}*

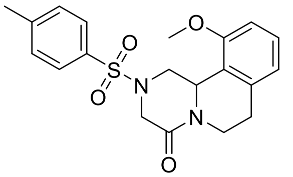

Yield 1.5 mg (6%) of amorphous solid. ^{1}H NMR (400 MHz, DMSO-d_6) δ = 7.68 - 7.64 (m, 2 H), 7.43 - 7.38 (m, 2 H), 7.17 (t, *J* = 8.0 Hz, 1 H), 6.89 (d, *J* = 8.3 Hz, 1 H), 6.72 (d, *J* = 7.3 Hz, 1 H), 4.82 (dd, *J* = 10.1, 3.4 Hz, 1 H), 4.57 - 4.51 (m, 1 H), 4.49 - 4.42 (m, 1 H), 4.00 (d, *J* = 16.6 Hz, 1 H), 3.81 (s, 3 H), 3.68 (s, 1 H), 3.43 (d, *J* = 16.6 Hz, 1 H), 2.71 - 2.62 (m, 1 H), 2.61 - 2.54 (m, 2 H), 2.37 - 2.33 (m, 3 H).^{13}C NMR (101 MHz, DMSO-d_6) δ = 163.0, 156.2, 144.0, 137.5, 132.7, 130.0, 128.3, 127.5, 121.4, 120.0, 109.3, 55.6, 52.4, 48.0, 46.9, 37.6, 28.8, 21.0. HRMS (ESI-TOF): *m/z* calcd for $C_{20}H_{23}N_2O_4S$ [M+H]$^+$ 387.1373, found 387.1373.

3. ^{1}H and ^{13}C NMR spectra

^{1}H and ^{13}C NMR spectra (d_6-DMSO) for compound 6{*1,2,1,1*}

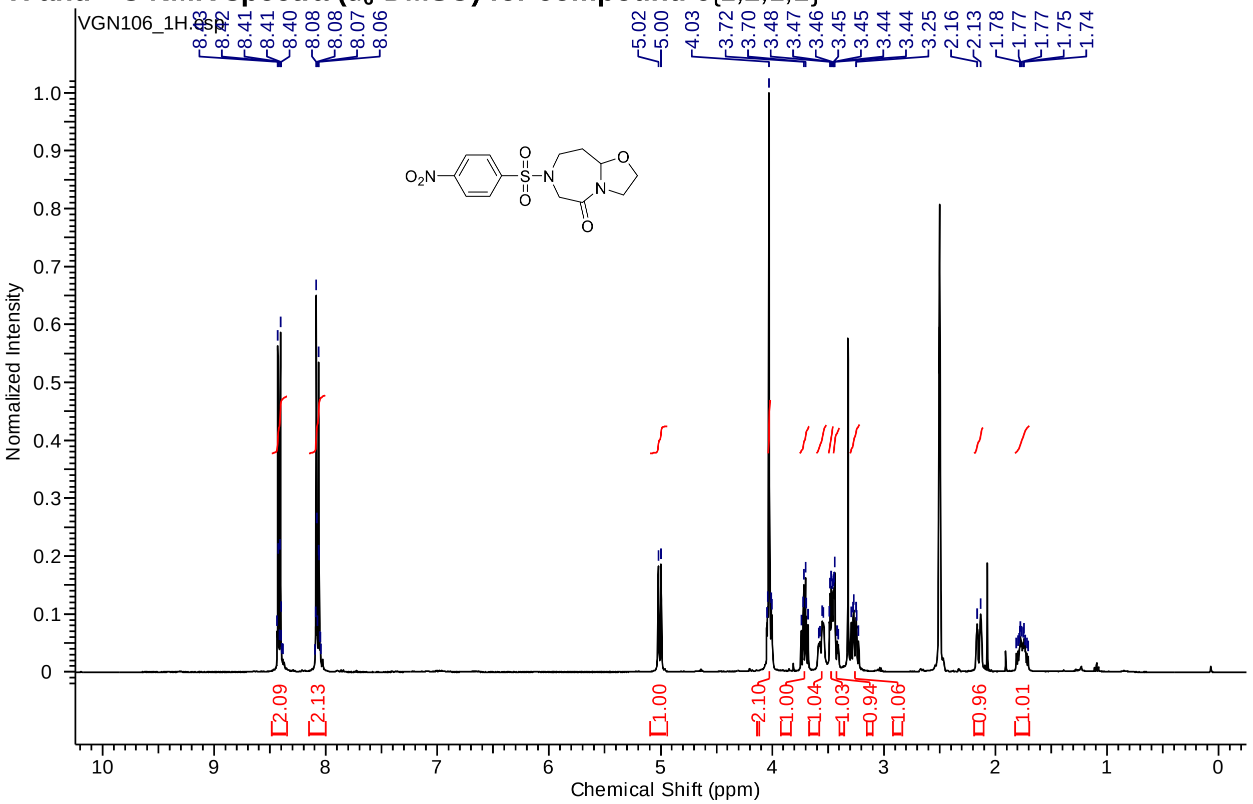

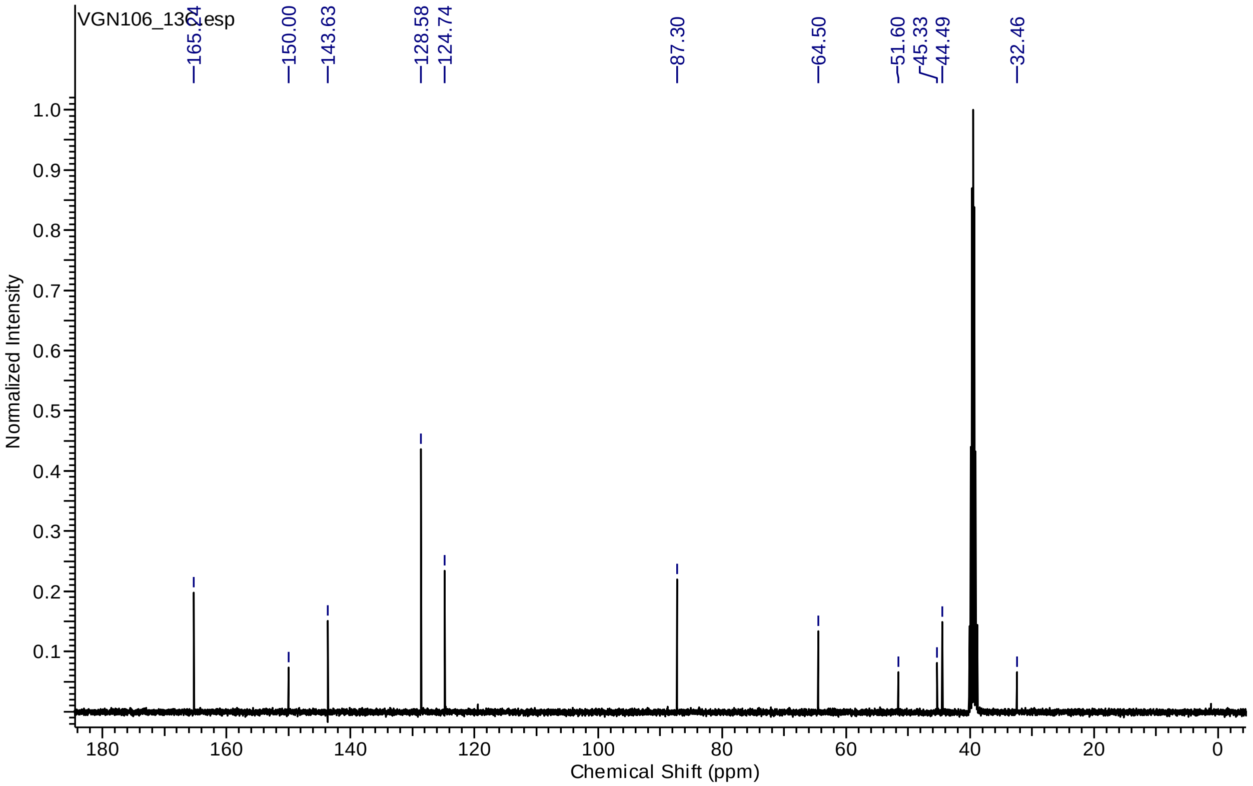

^{1}H and ^{13}C NMR spectra (d_6-DMSO) for compound 6{*1,2,1,2*}

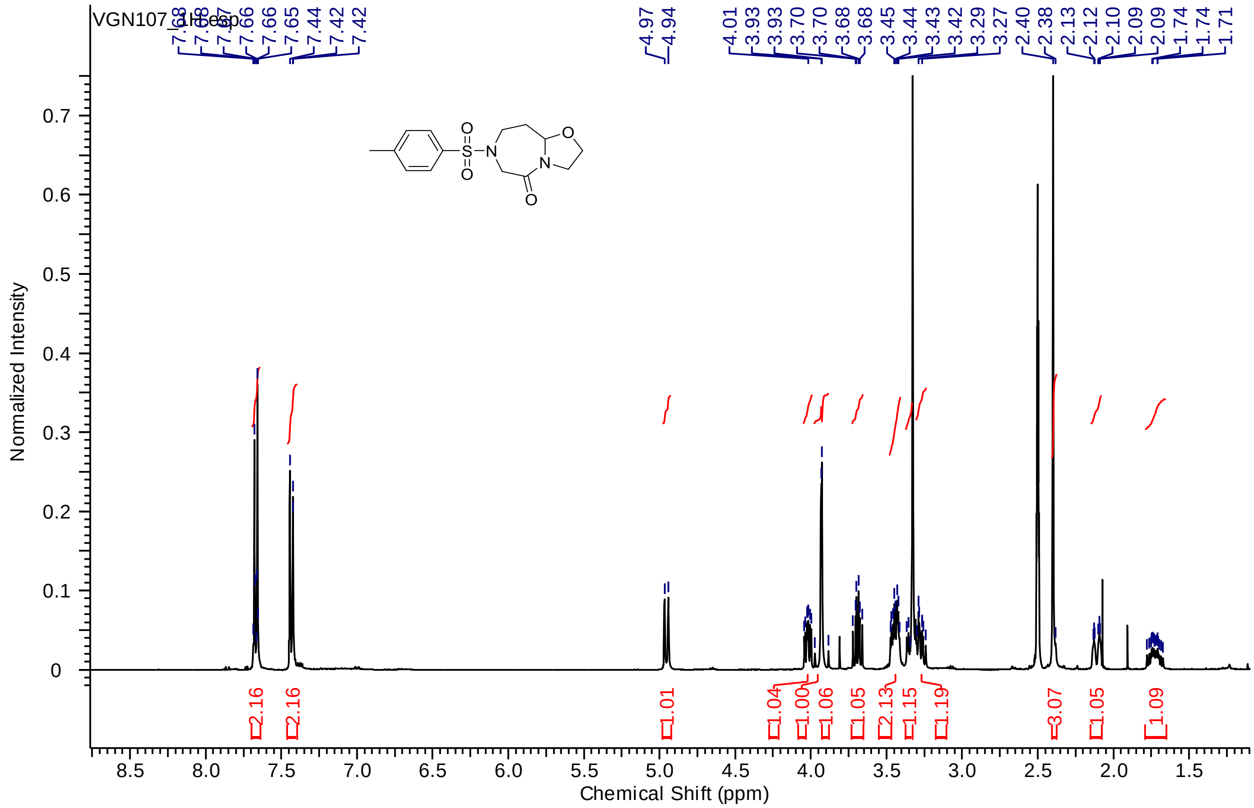

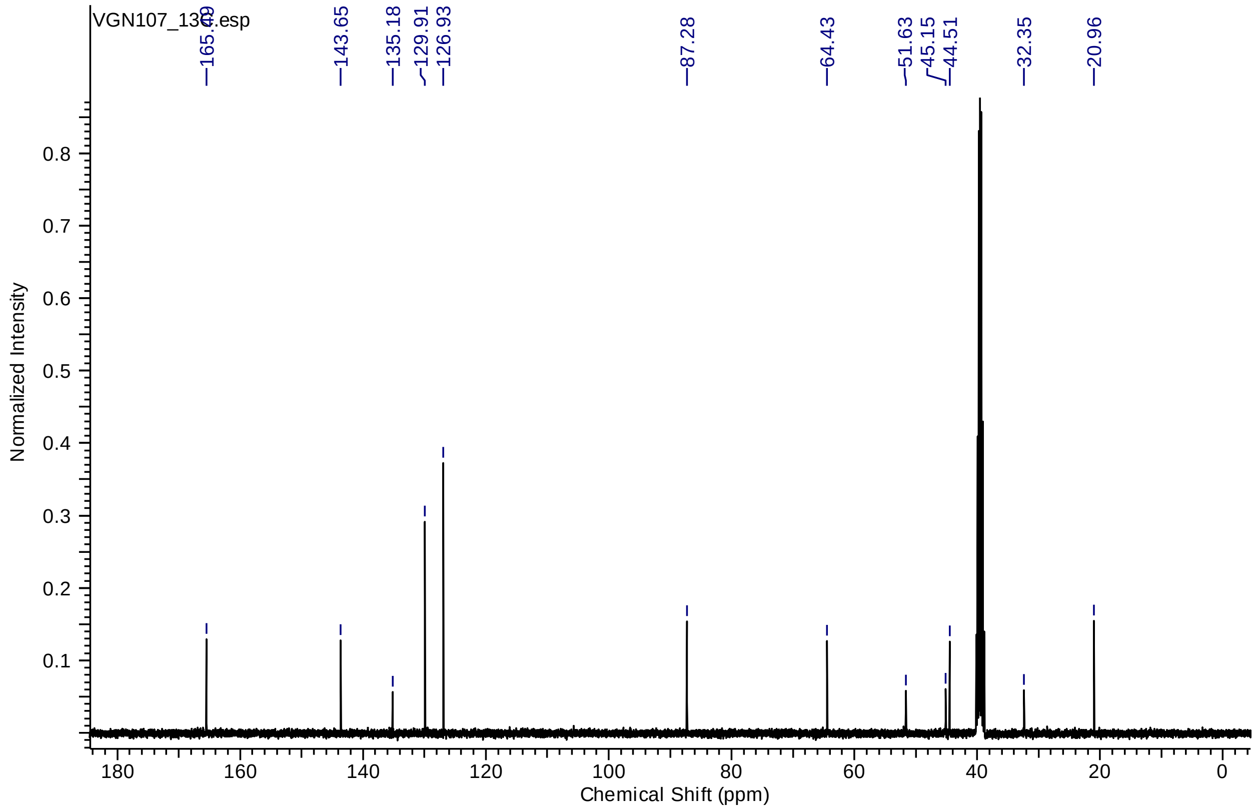

^{1}H and ^{13}C NMR spectra (d_6-DMSO) for compound 6{*1,2,1,3*}

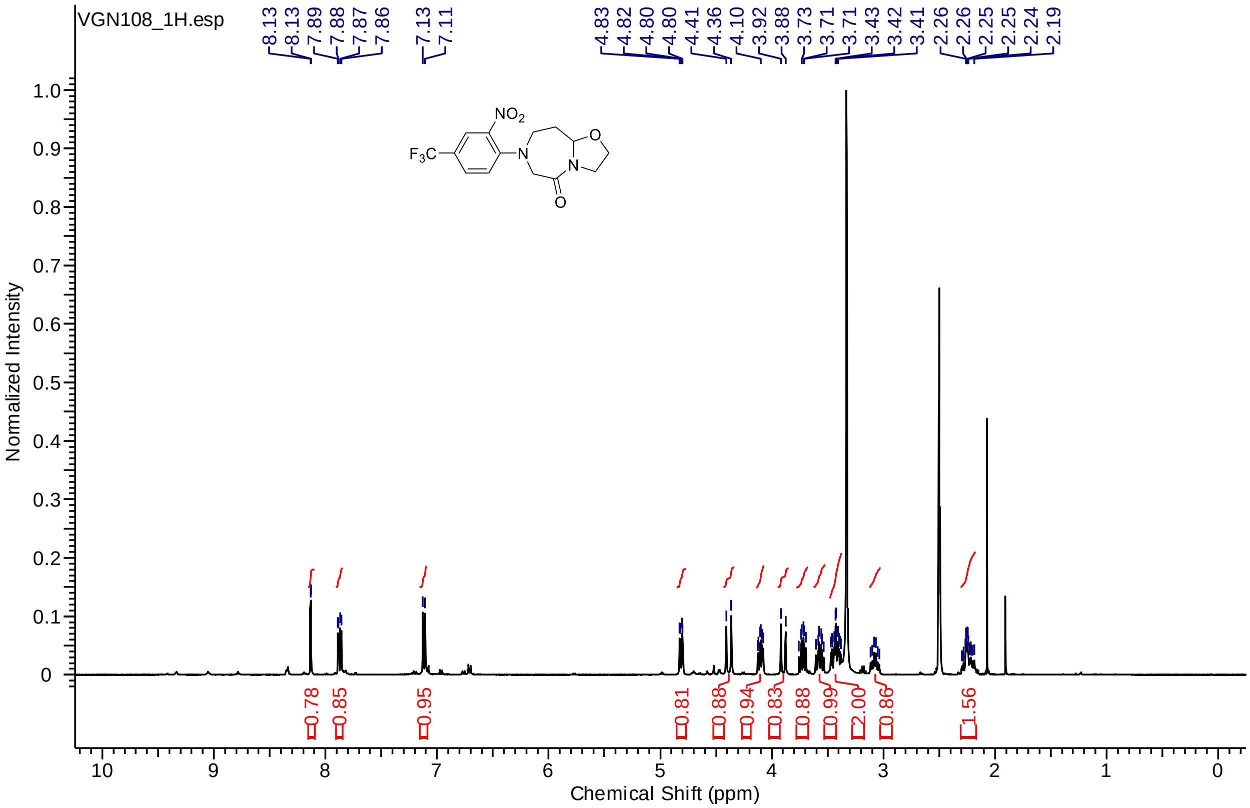

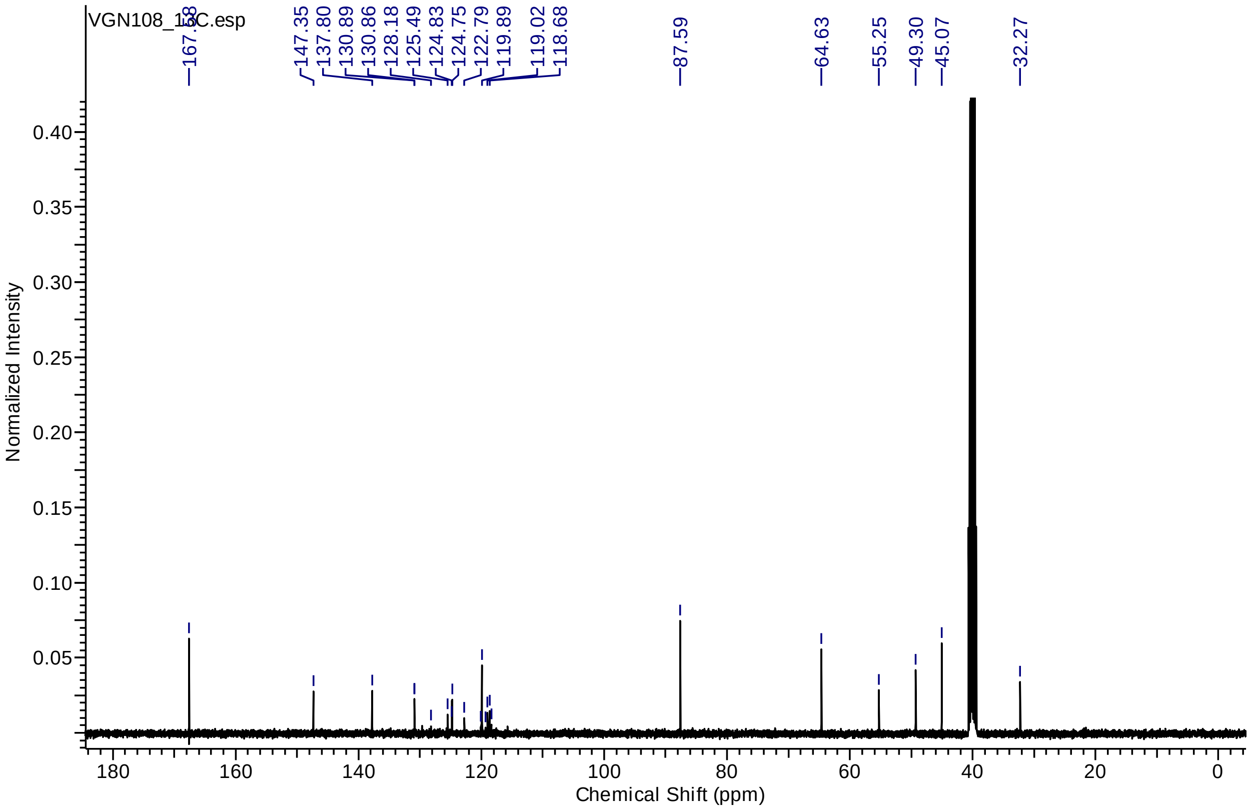

^{1}H and ^{13}C NMR spectra (d_6-DMSO) for compound (*R*,*R*)-6{*1,2,2,2*}

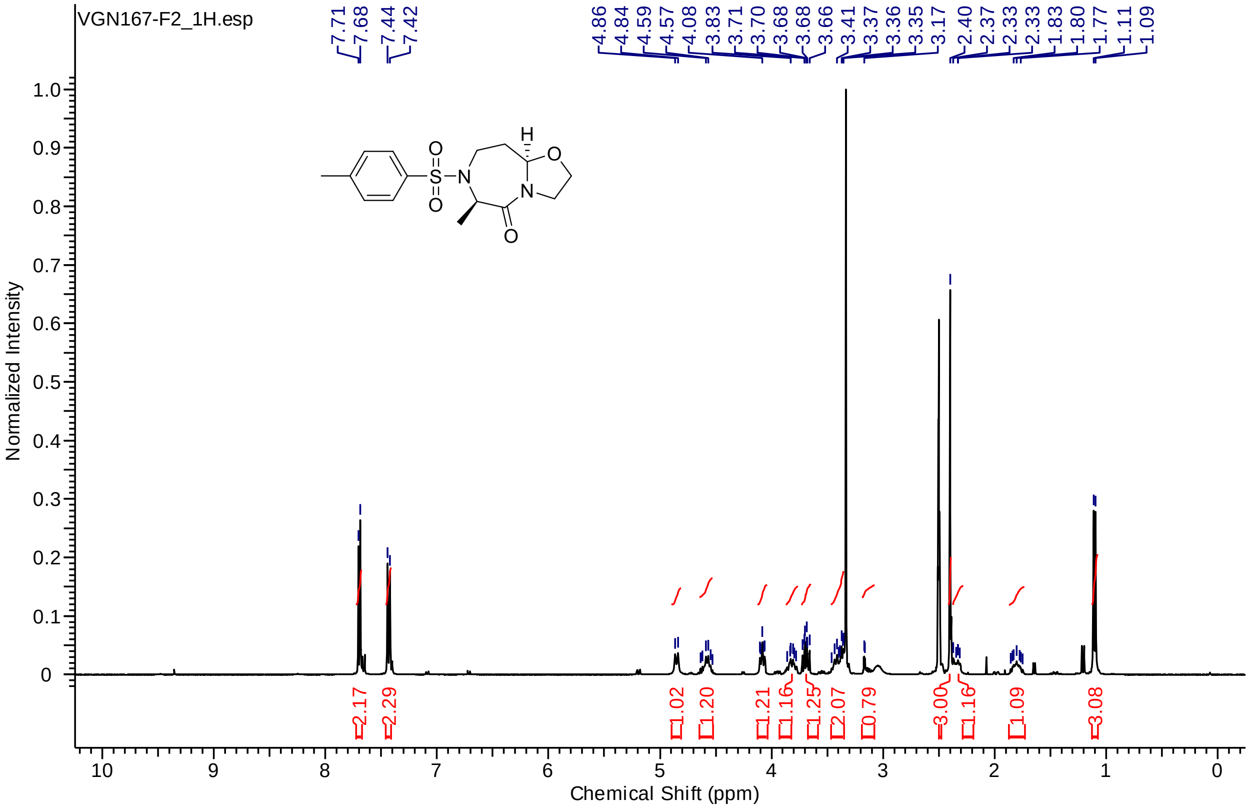

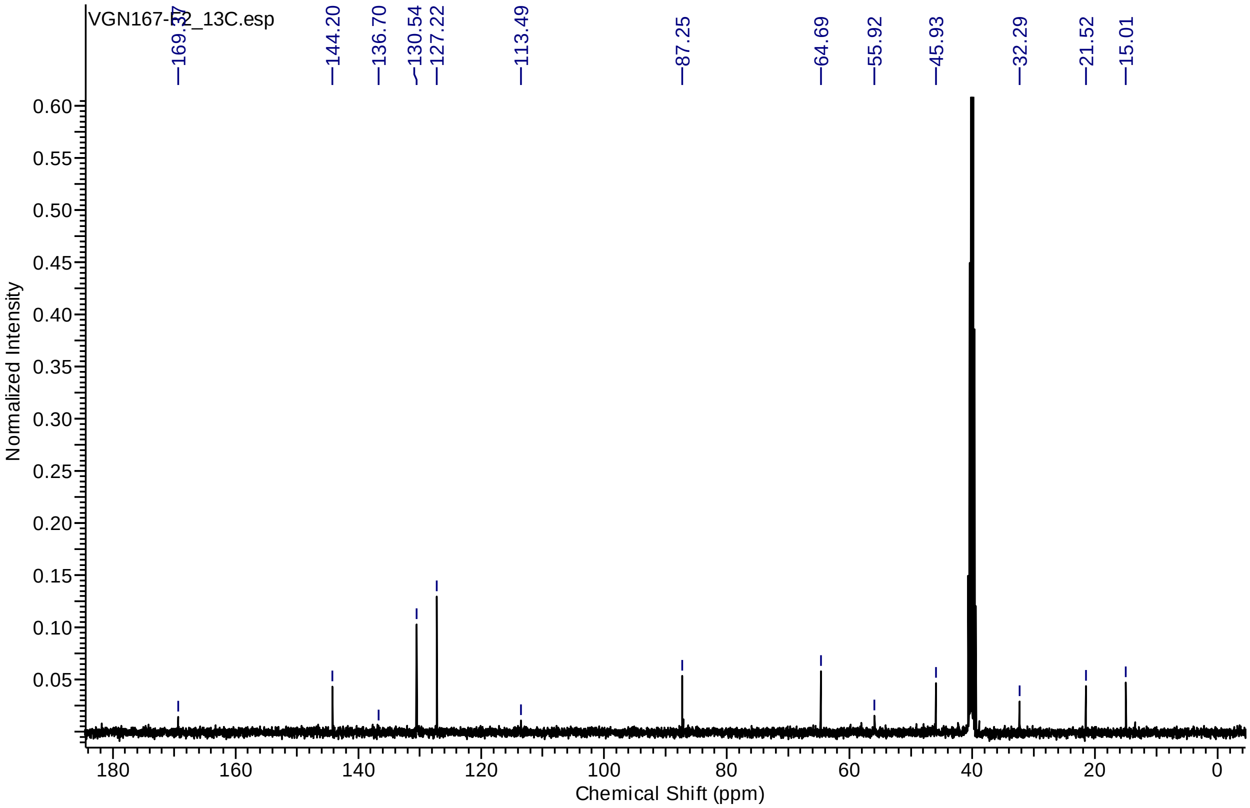

^{1}H and ^{13}C NMR spectra (d_6-DMSO) for compound (*R*,*S*)-6{*1,2,2,2*}

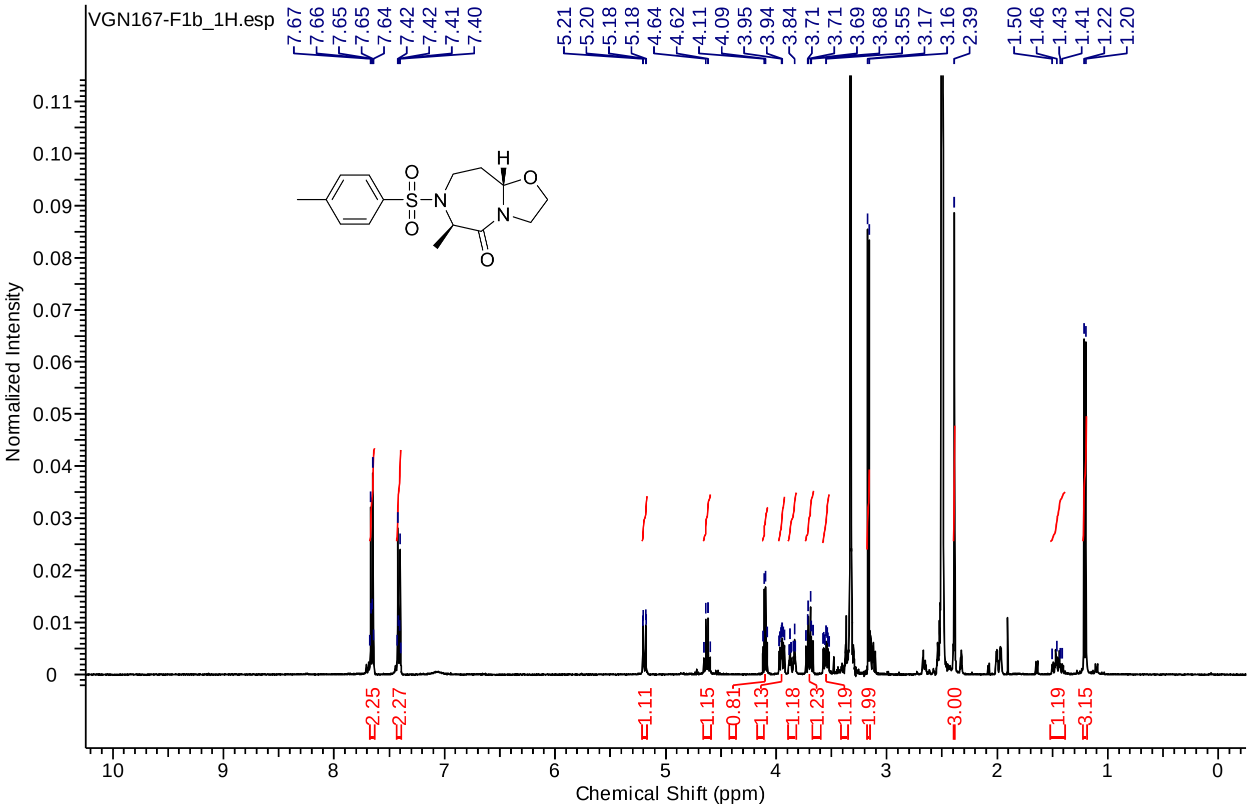

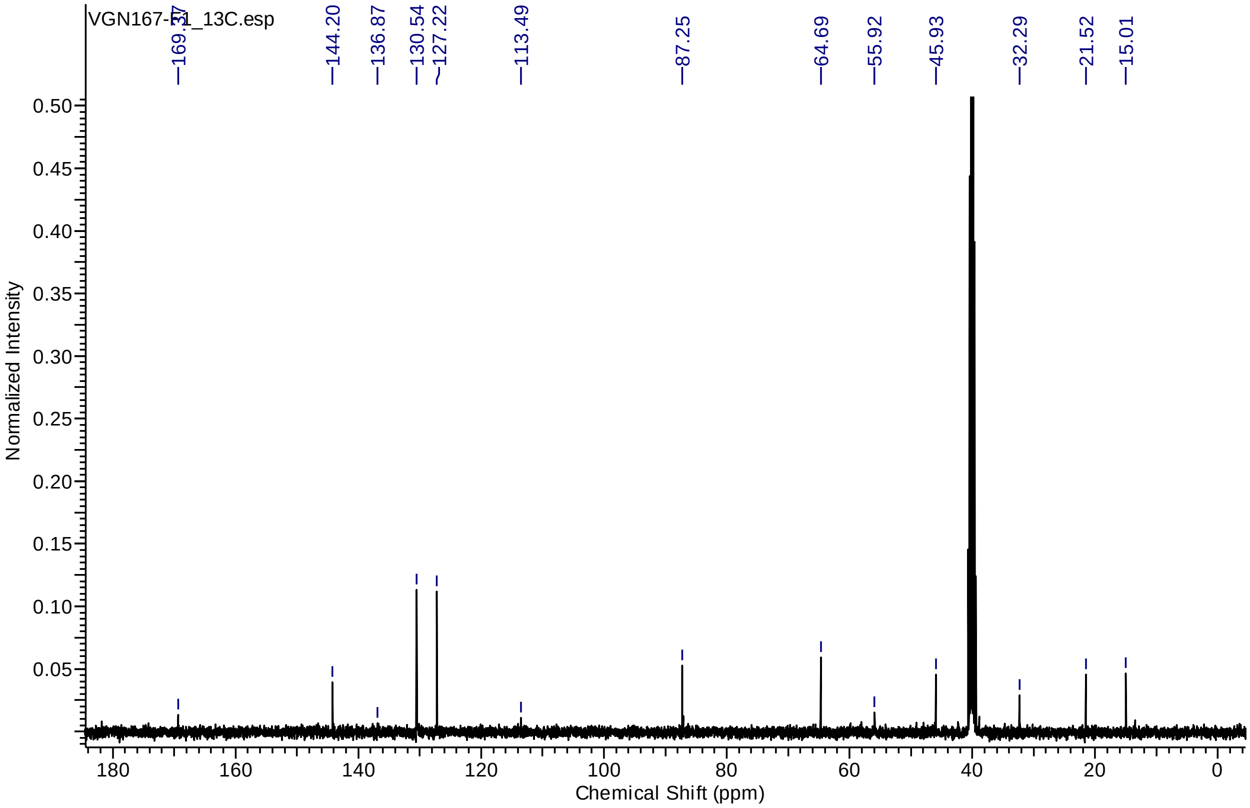

^{1}H and ^{13}C NMR spectra (d_6-DMSO) for compound (*S*,*R*)-6{*1,2,3,2*}

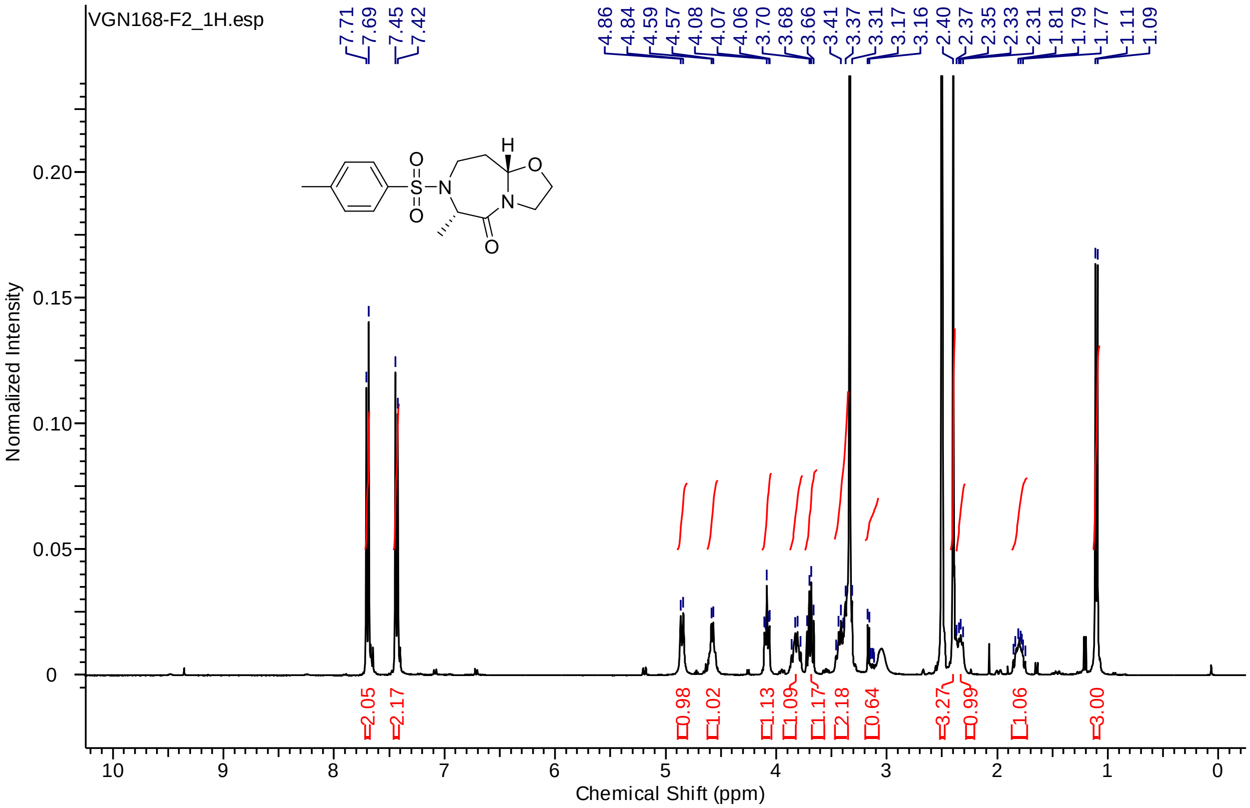

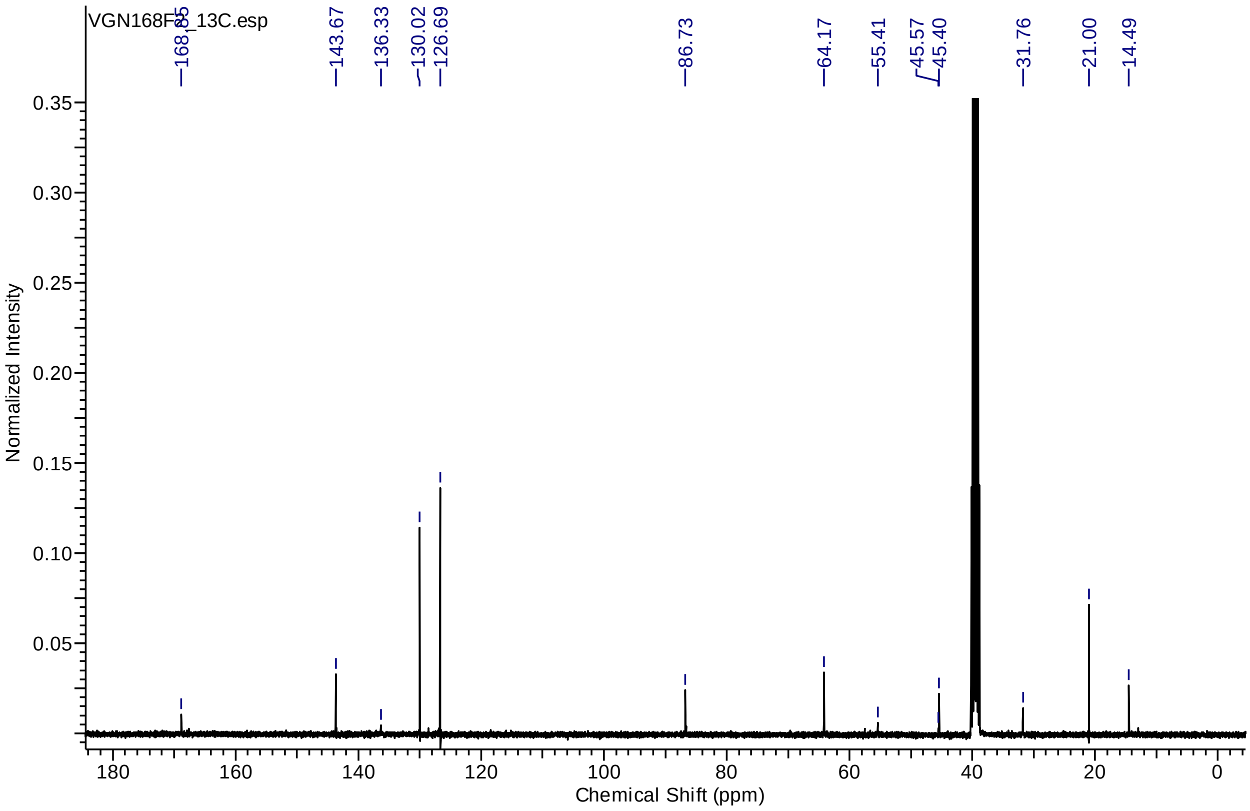

^{1}H and ^{13}C NMR spectra (d_6-DMSO) for compound (*S*,*S*)-6{*1,2,3,2*}

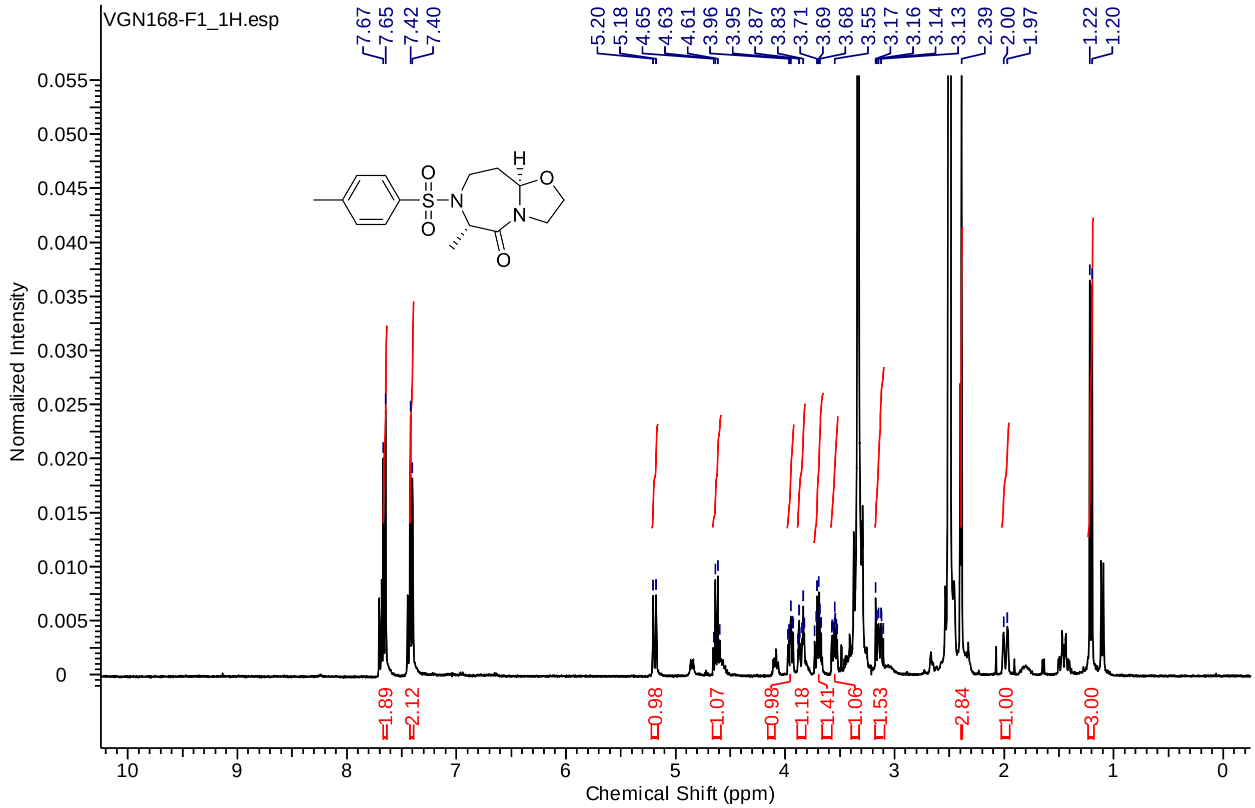

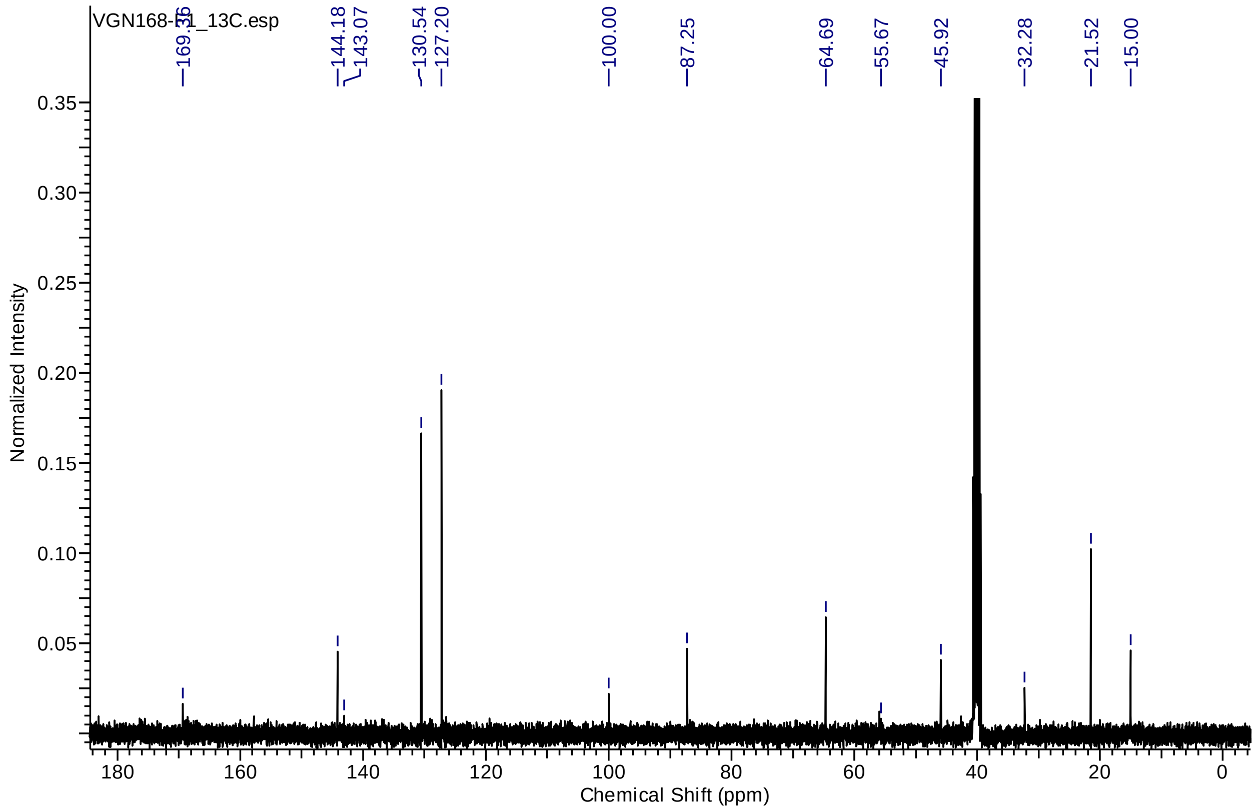

^{1}H and ^{13}C NMR spectra (d_6-DMSO) for compound 6{*1,3,1,1*}

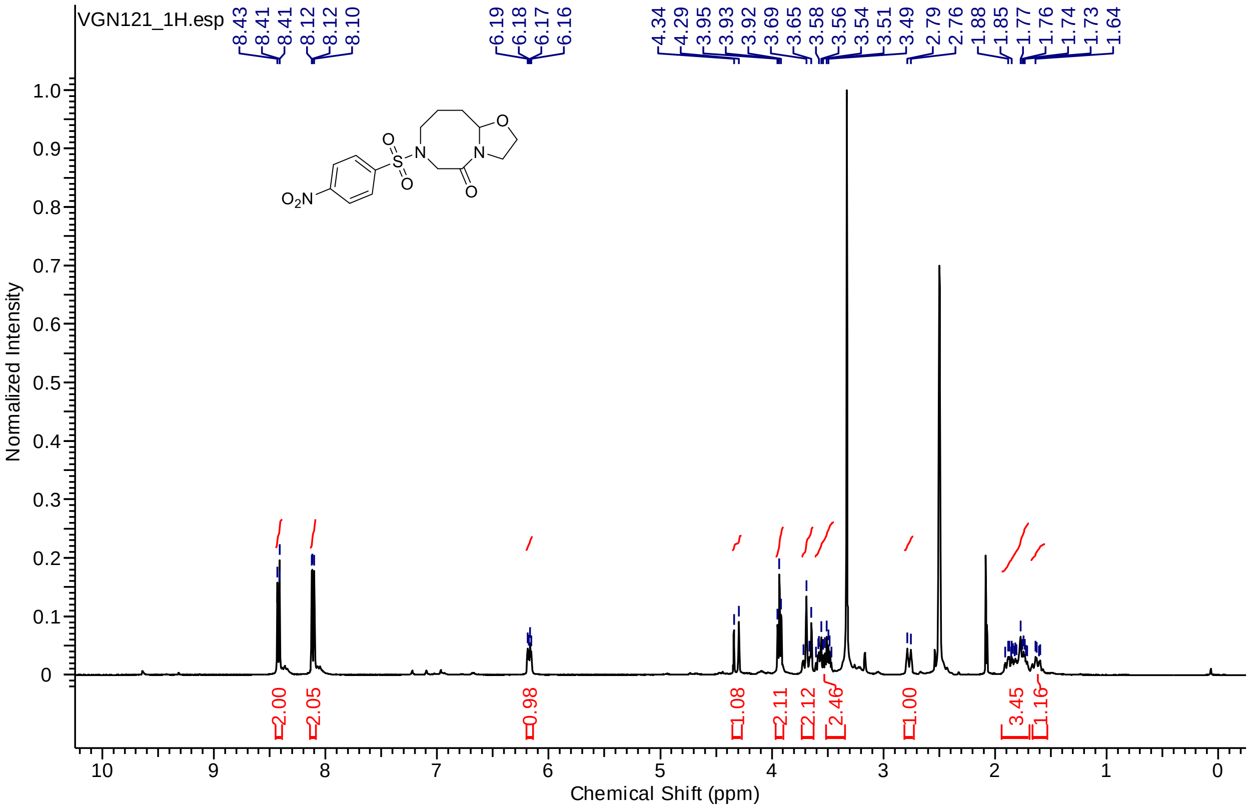

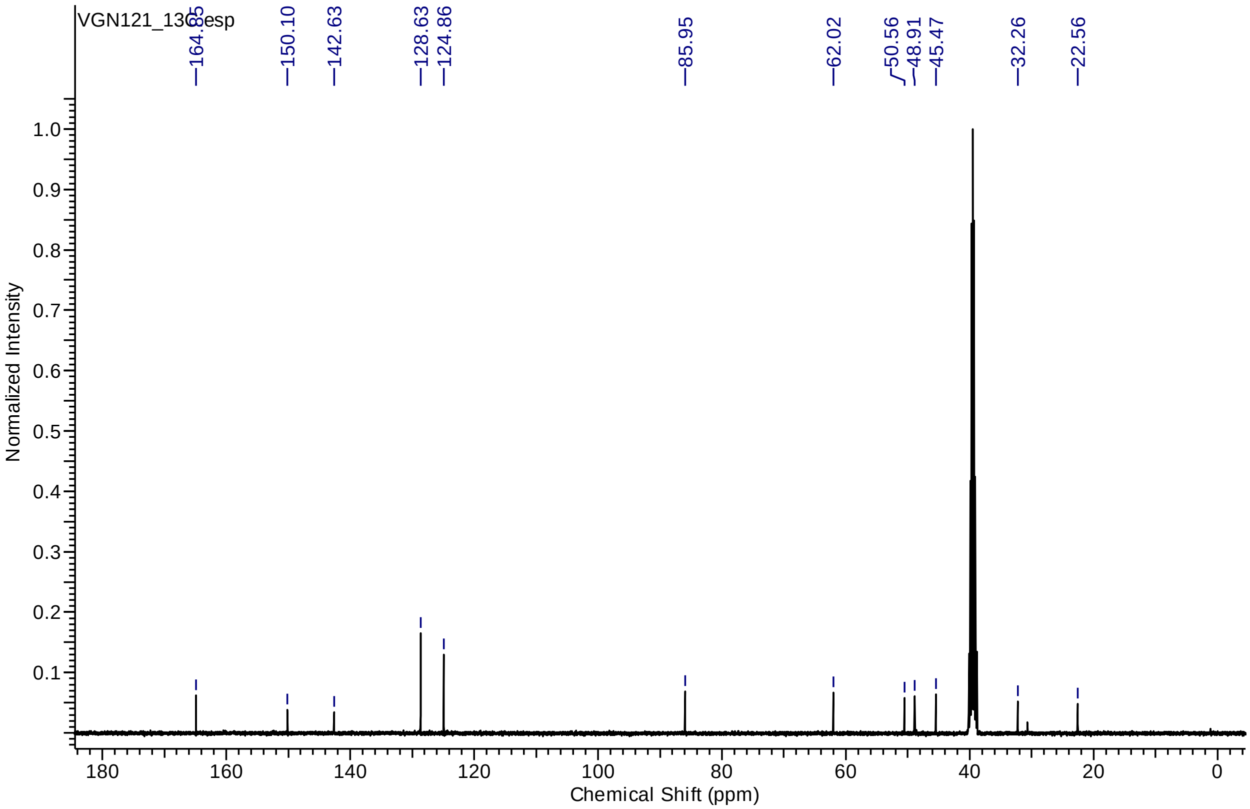

^{1}H and ^{13}C NMR spectra (d_6-DMSO) for compound 6{*1,3,1,2*}

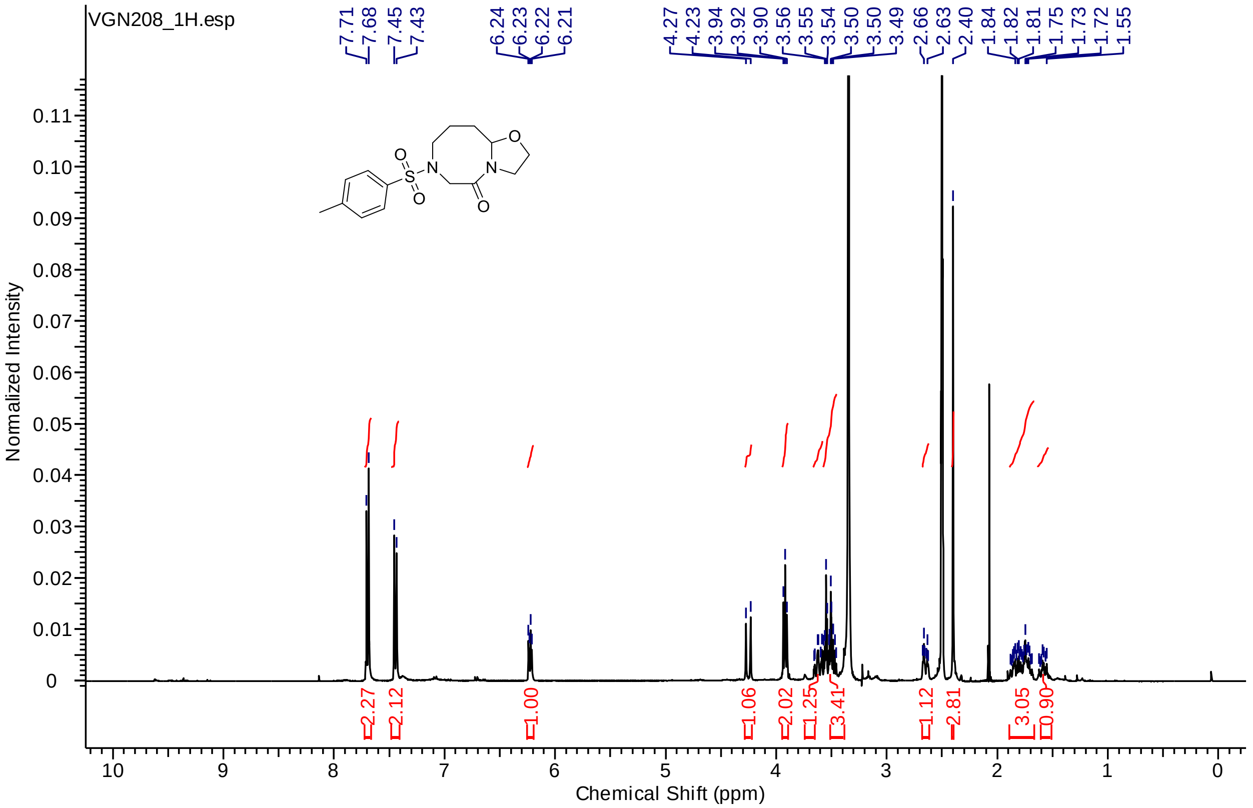

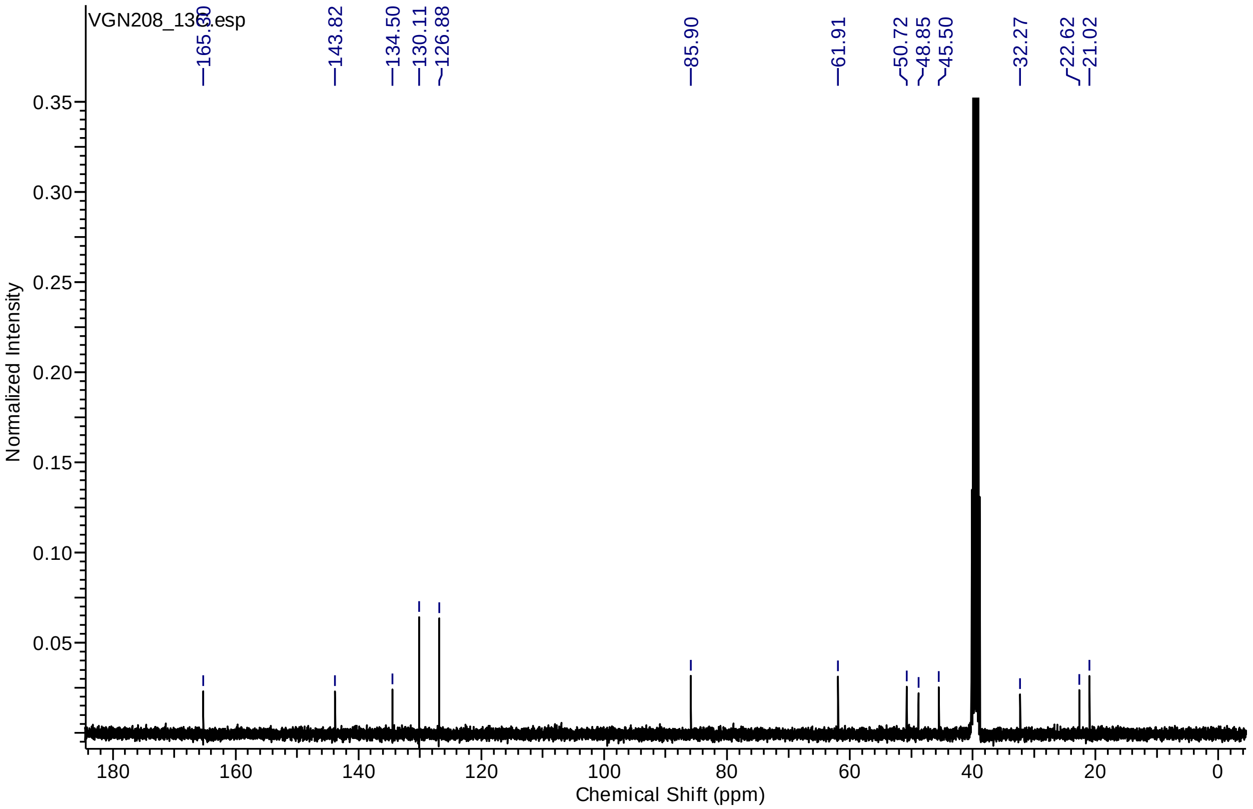

^{1}H and ^{13}C NMR spectra (d_6-DMSO) for compound **6**{*2,1,1,3*}

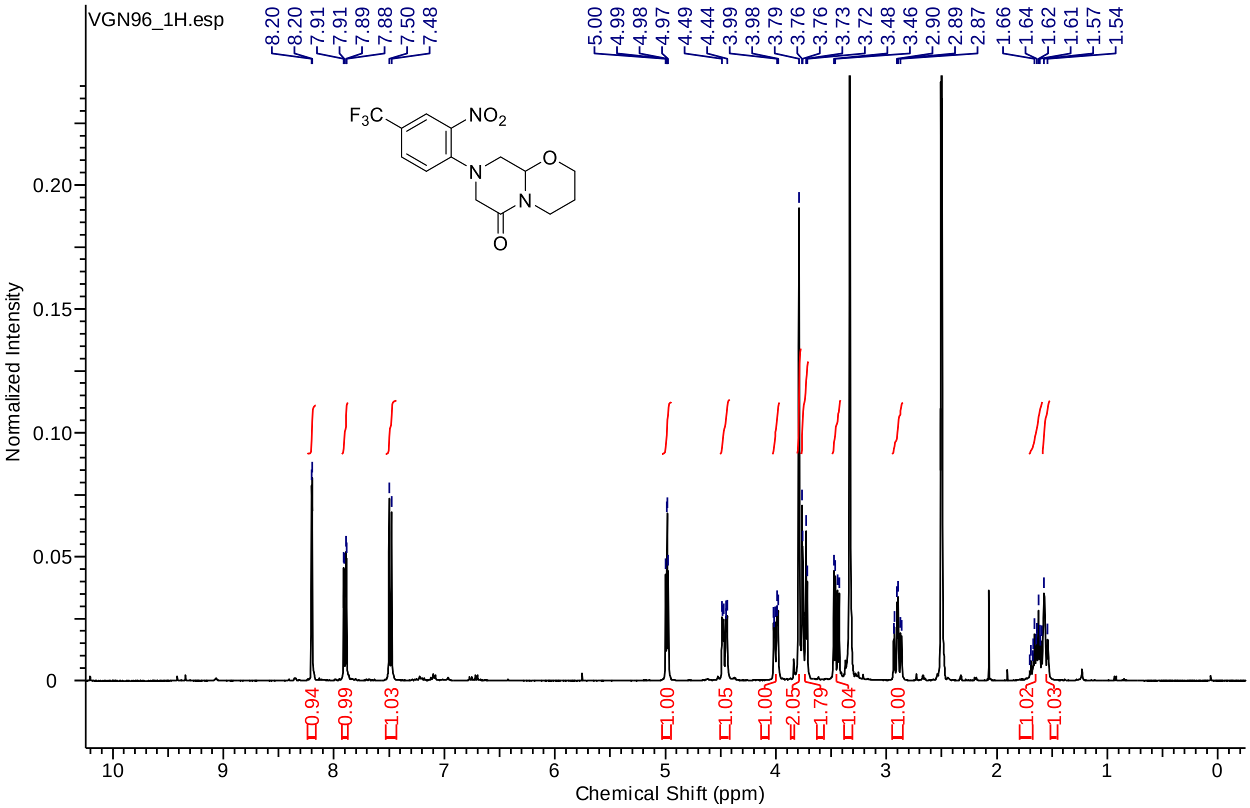

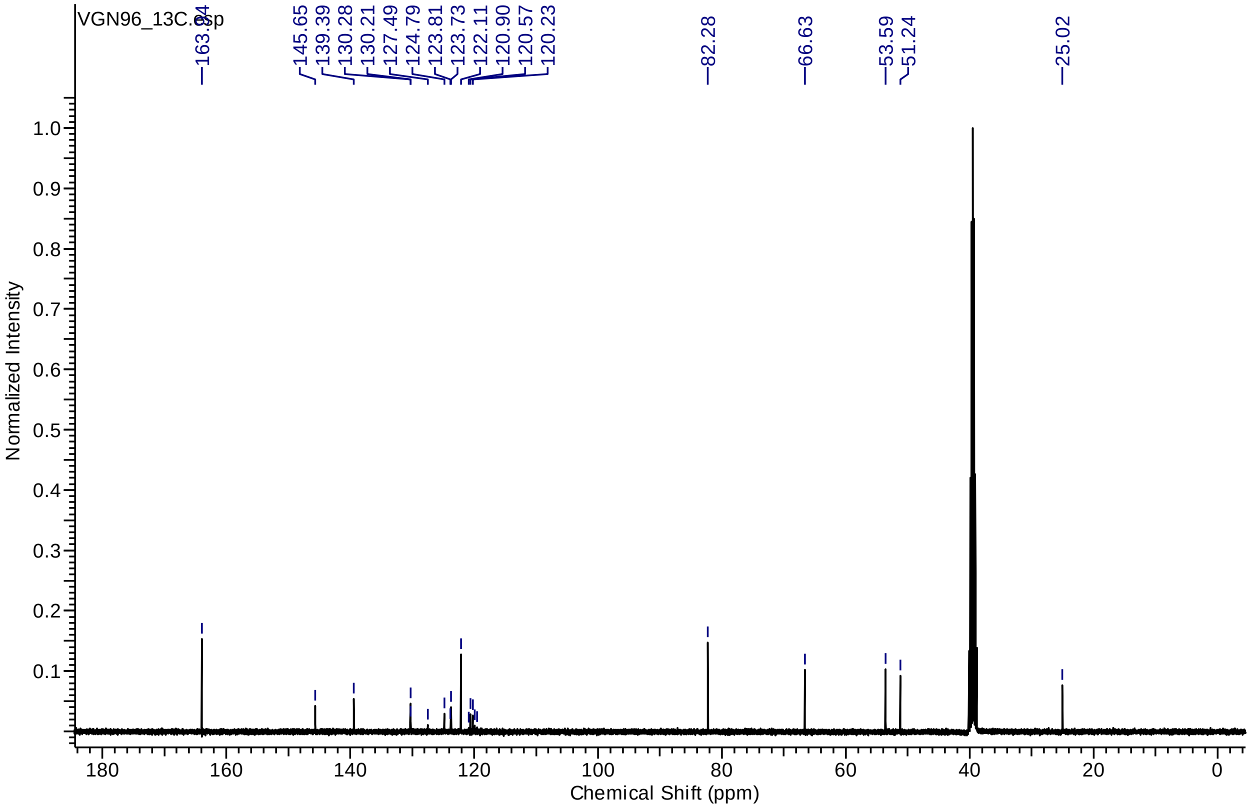

^{1}H and ^{13}C NMR spectra (d_6-DMSO) for compound 6*{2,2,1,1}*

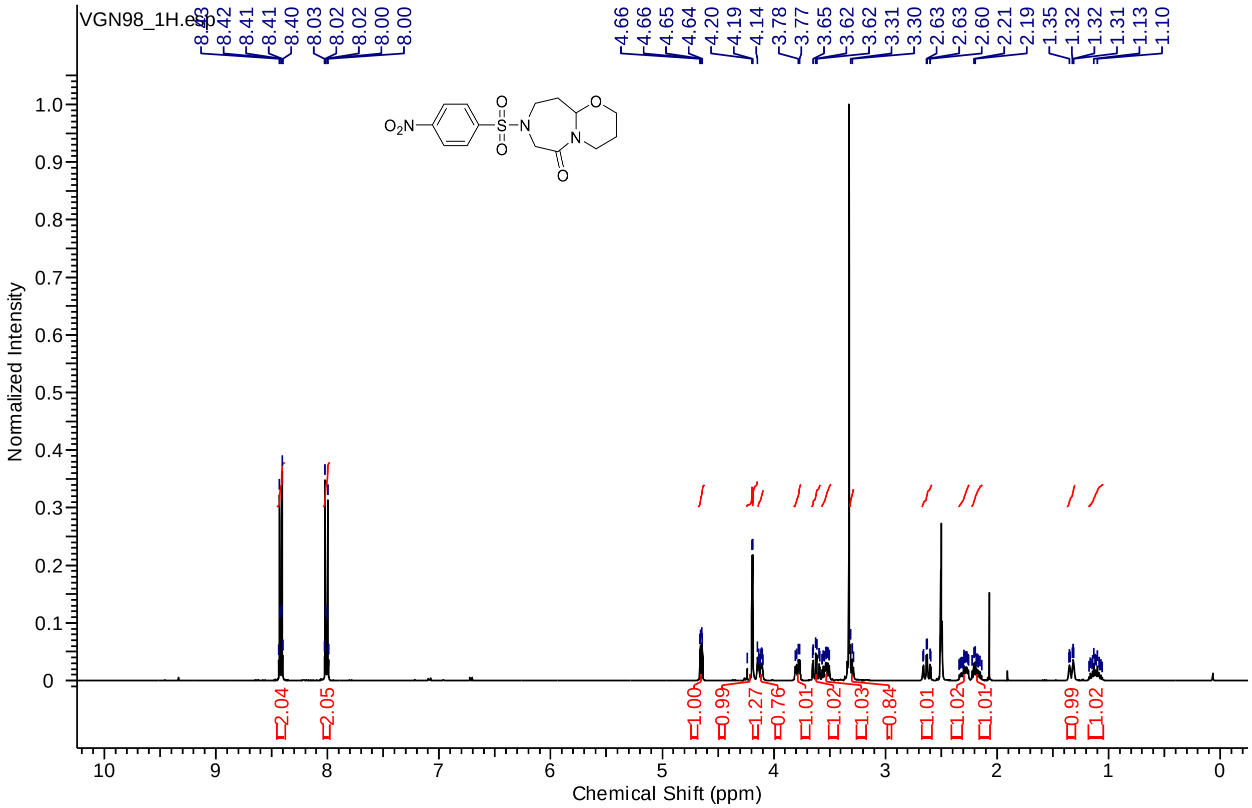

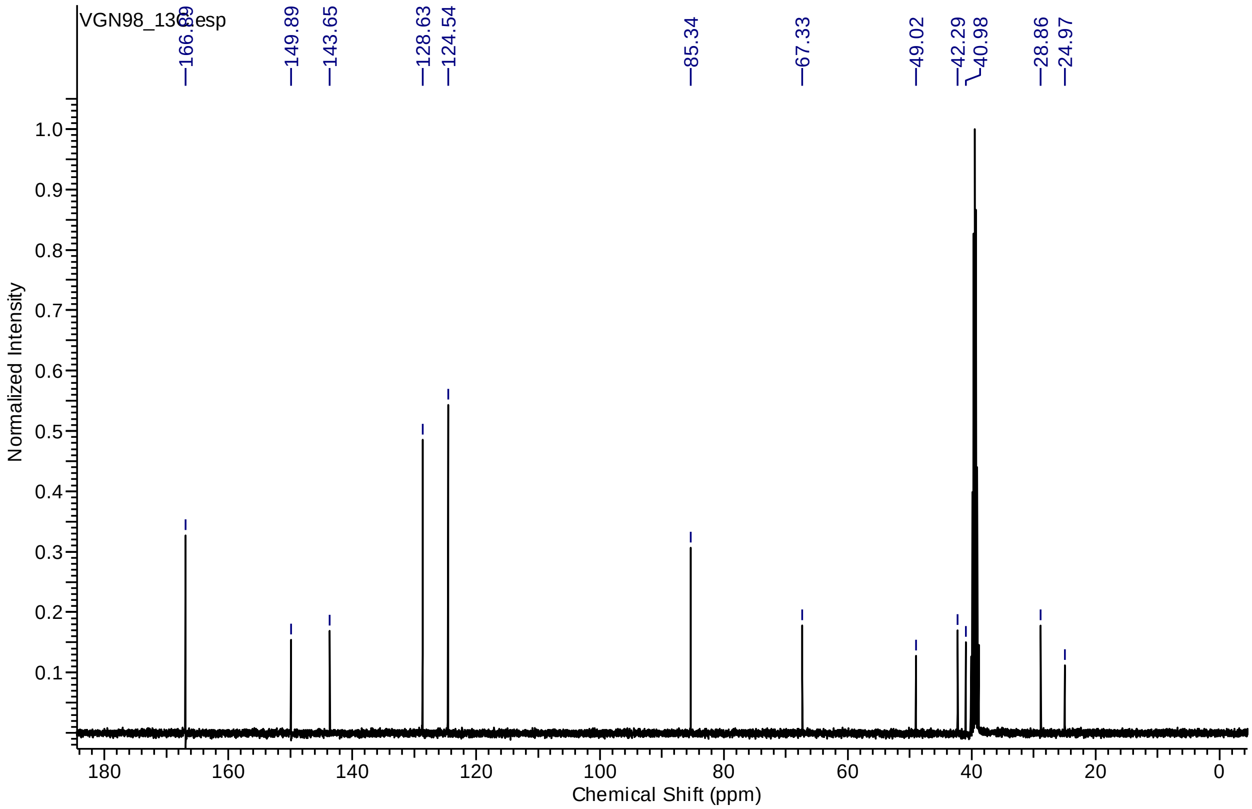

^{1}H and ^{13}C NMR spectra (d_6-DMSO) for compound 6{*2,2,1,2*}

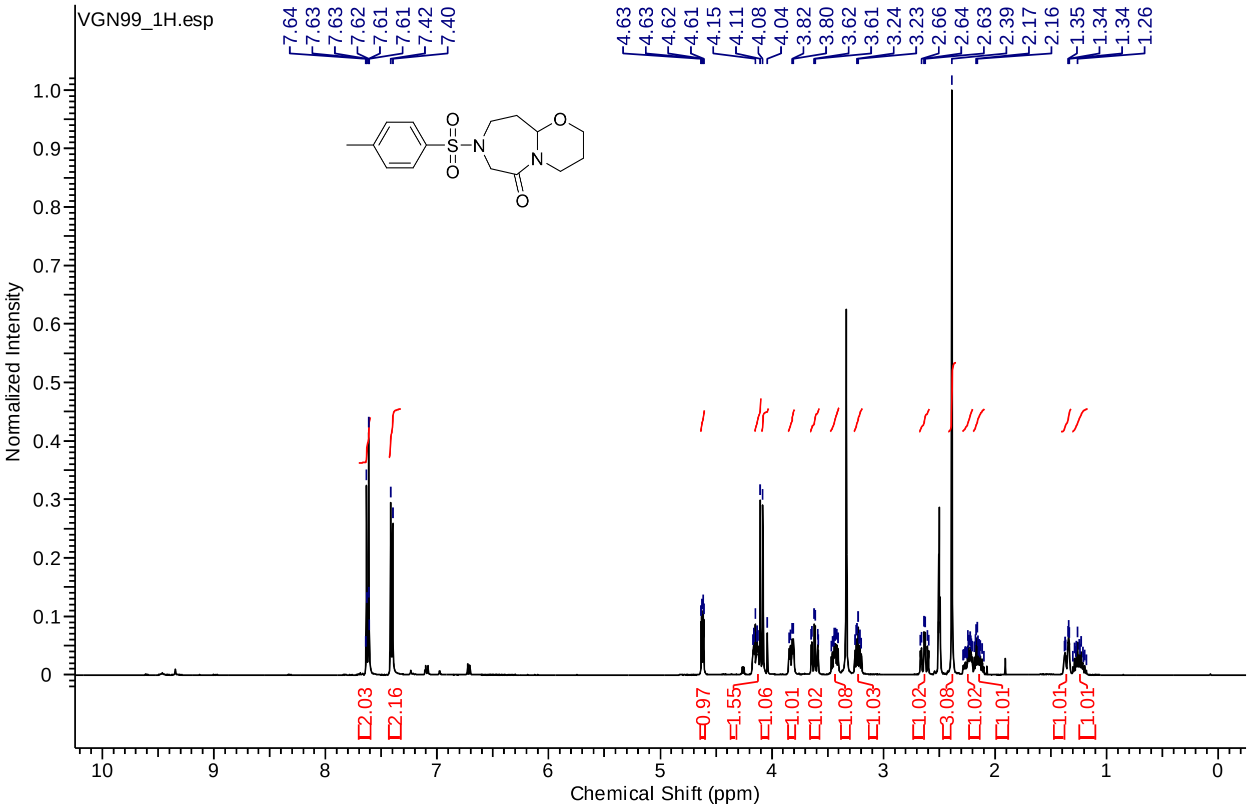

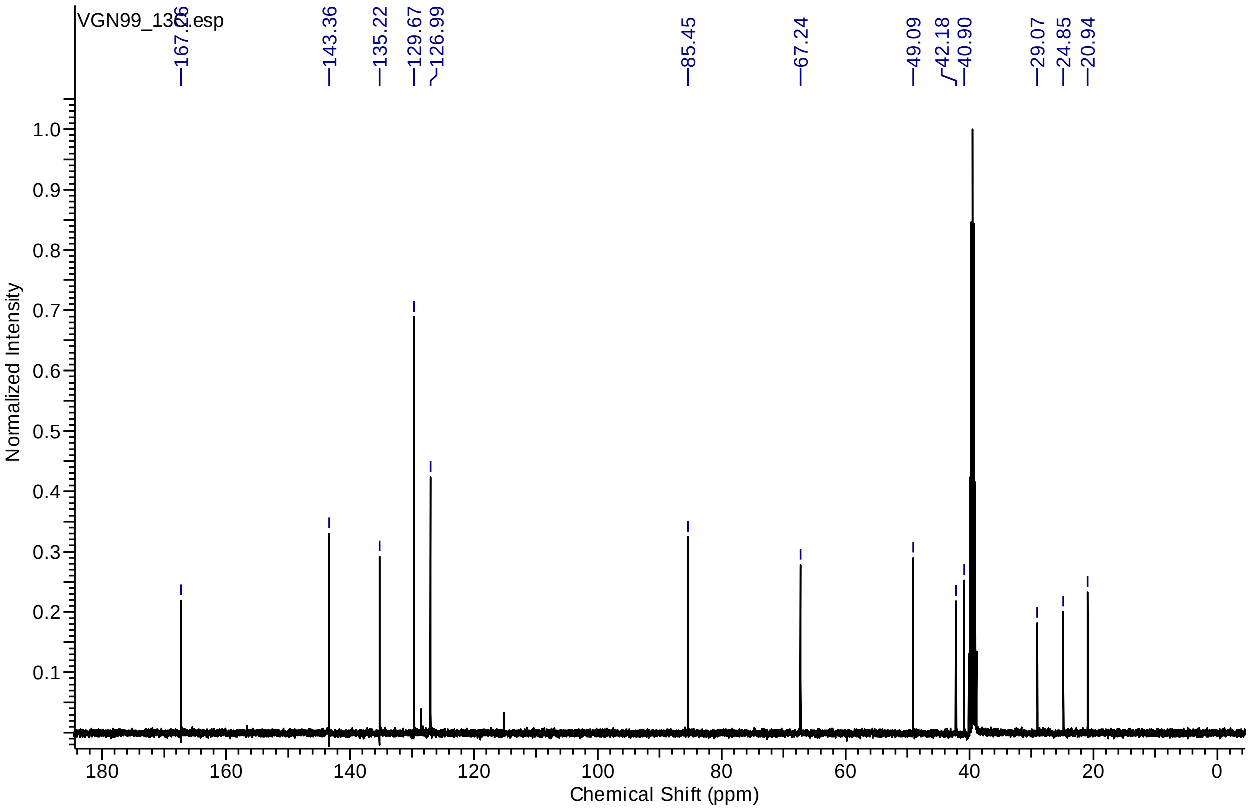

^{1}H and ^{13}C NMR spectra (d_6-DMSO) for compound 6{*2,2,1,3*}

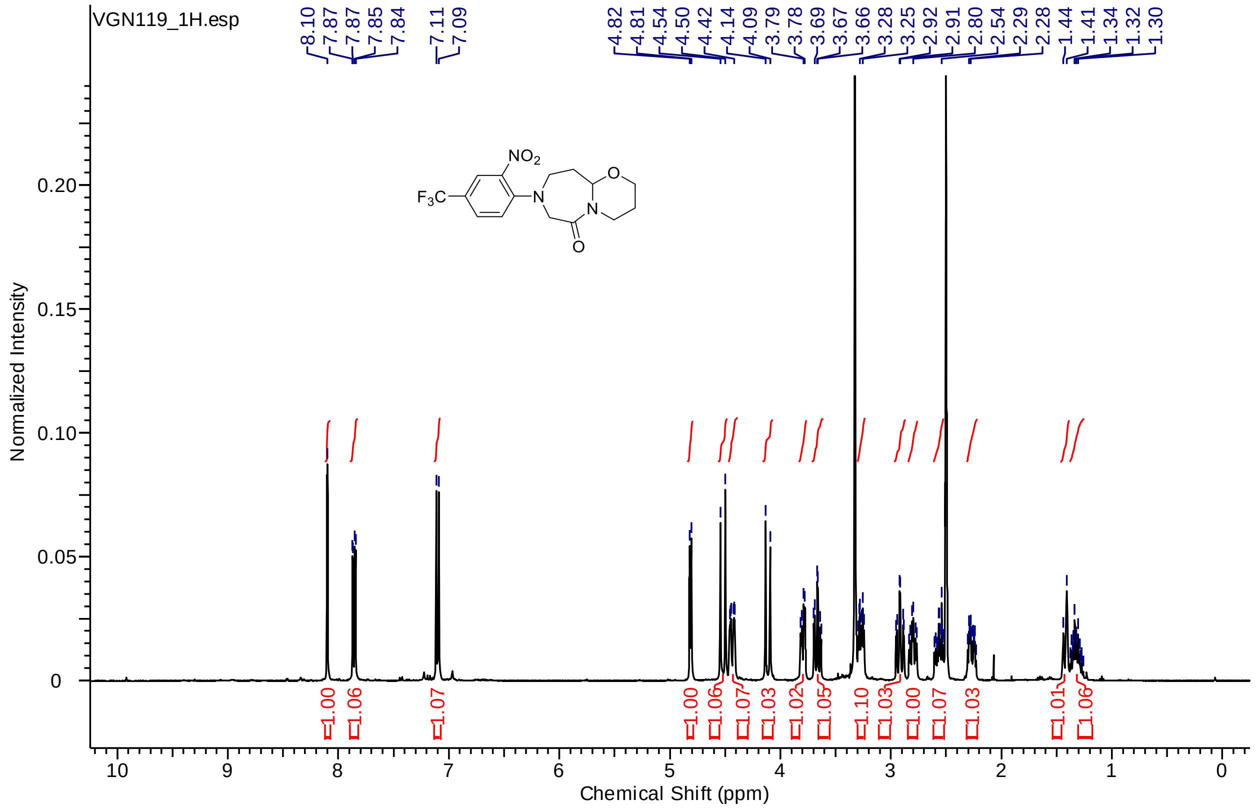

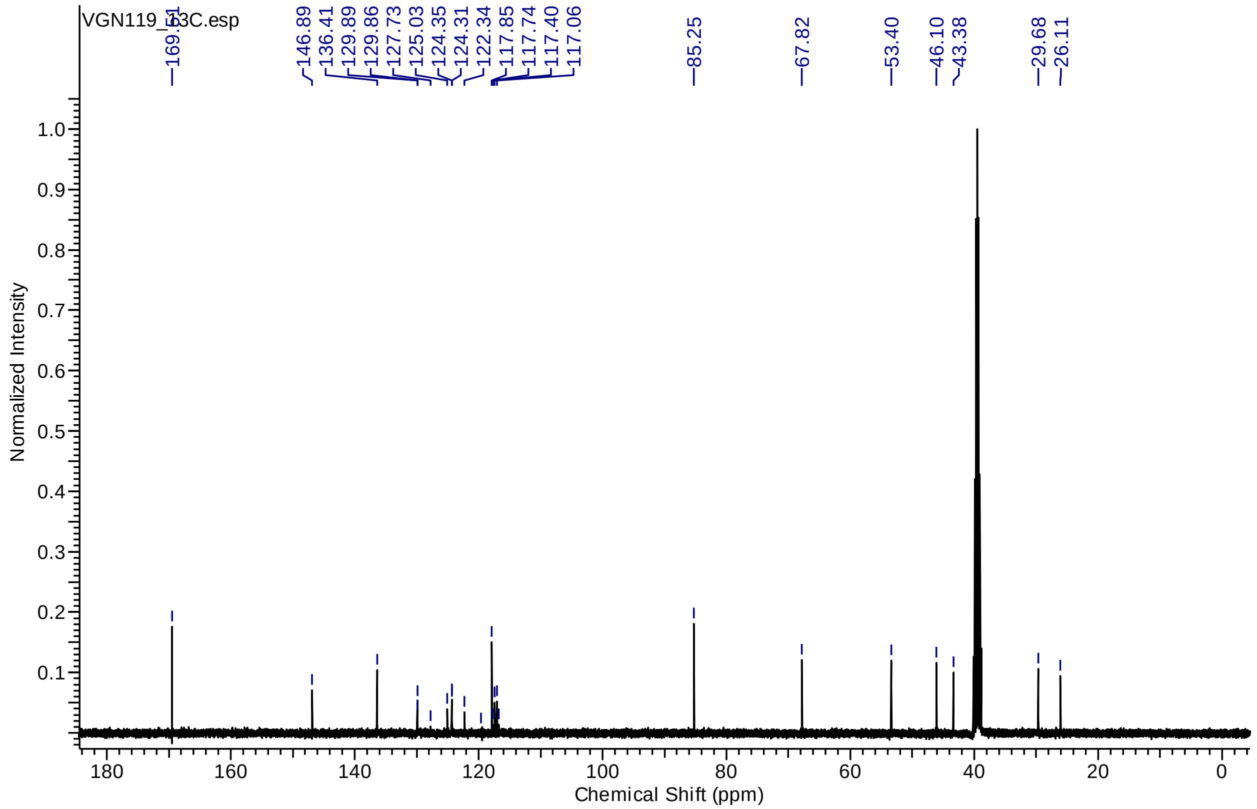

^{1}H and ^{13}C NMR spectra (d_6-DMSO) for compound [(*S,S*)-6{*2,2,3,2*}]

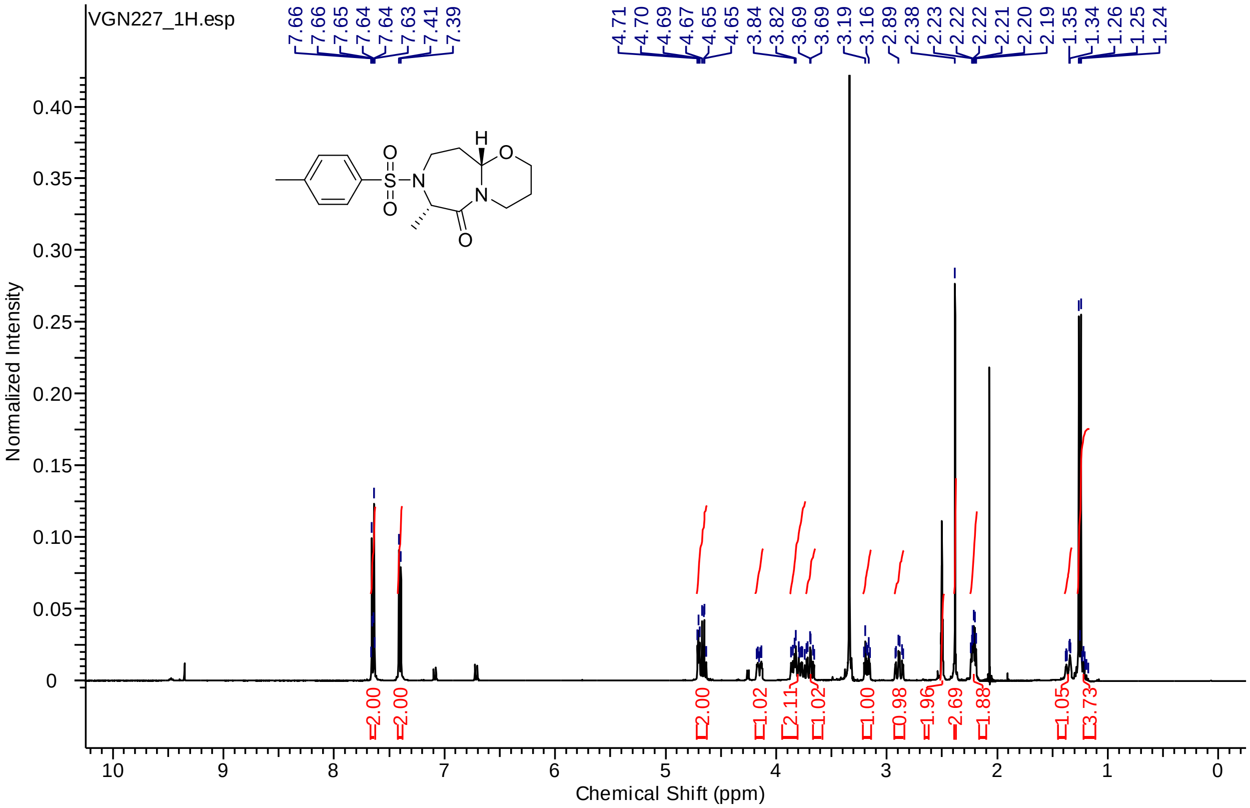

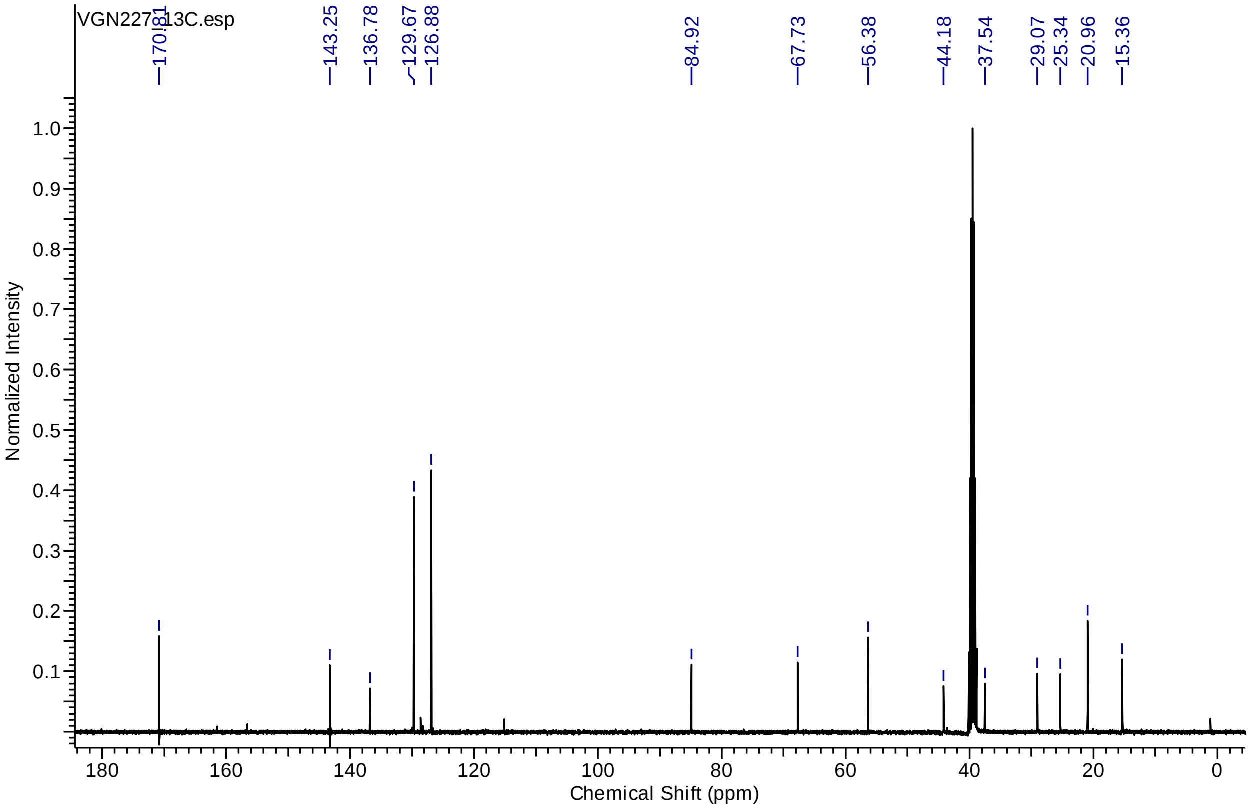

^{1}H and ^{13}C NMR spectra (d_6-DMSO) for compound [(*S,R*)-6{*2,2,3,2*}]

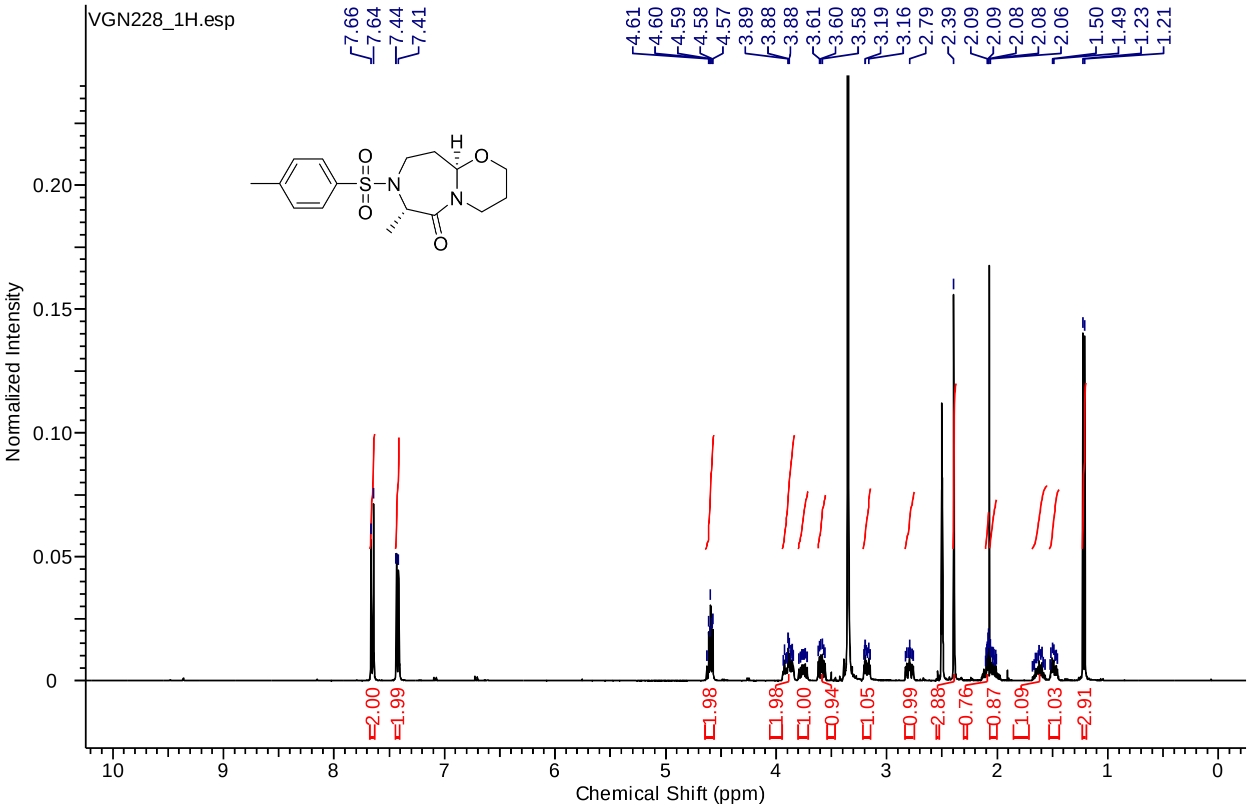

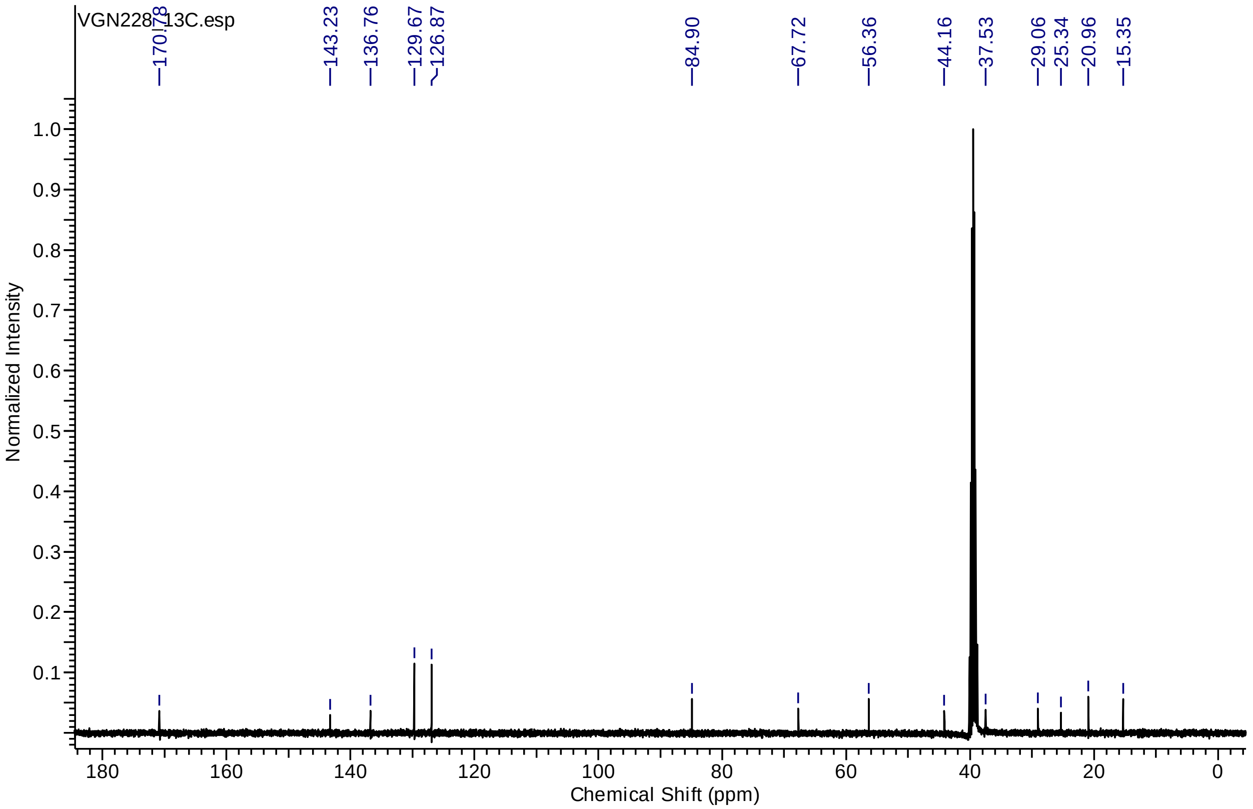

^{1}H and ^{13}C NMR spectra (d_6-DMSO) for compound 6{*3,1,1,3*}

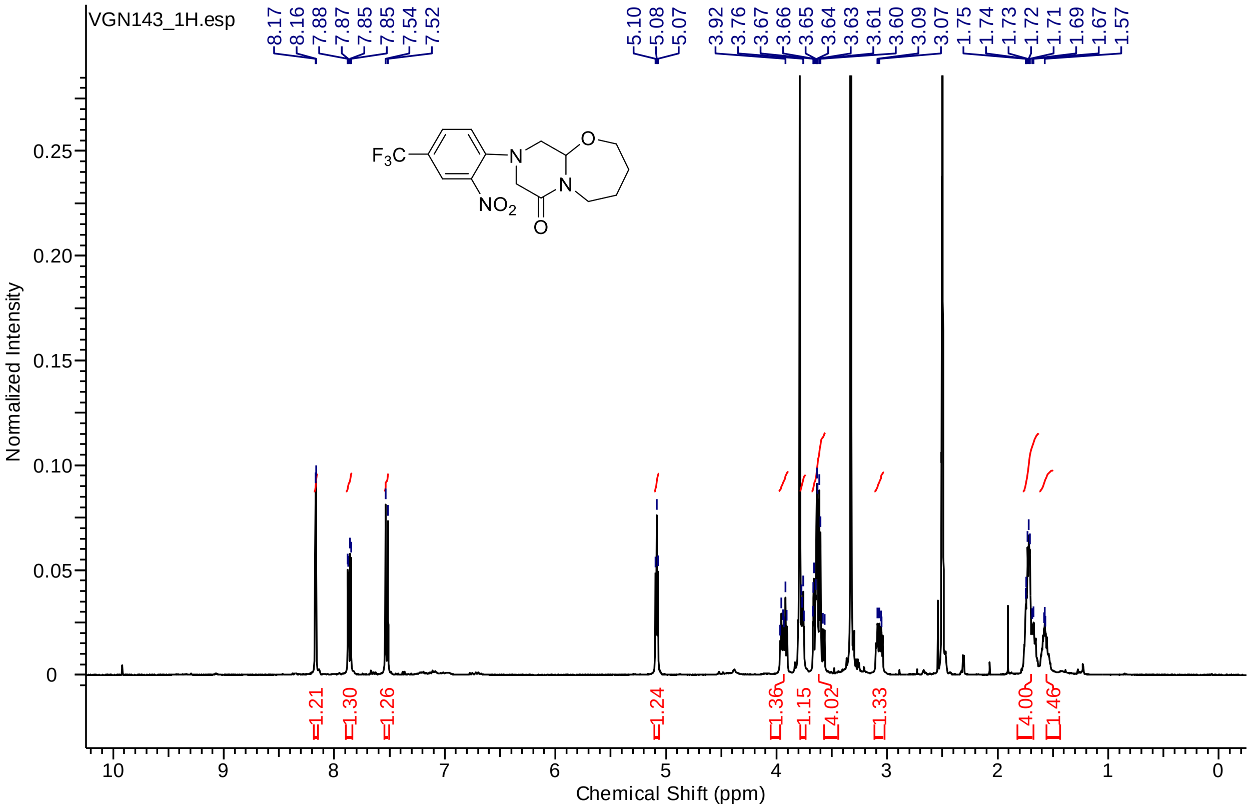

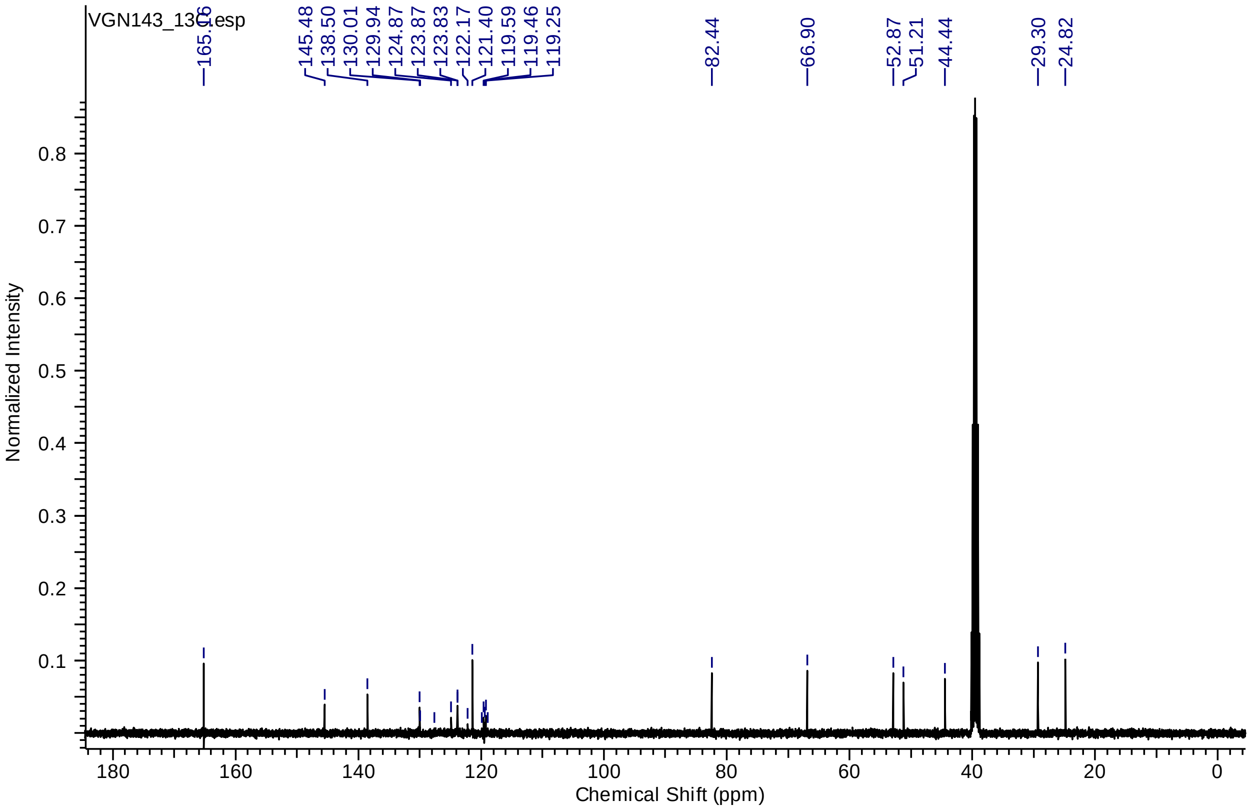

^{1}H and ^{13}C NMR spectra (d_6-DMSO) for compound 6{*3,2,1,2*}

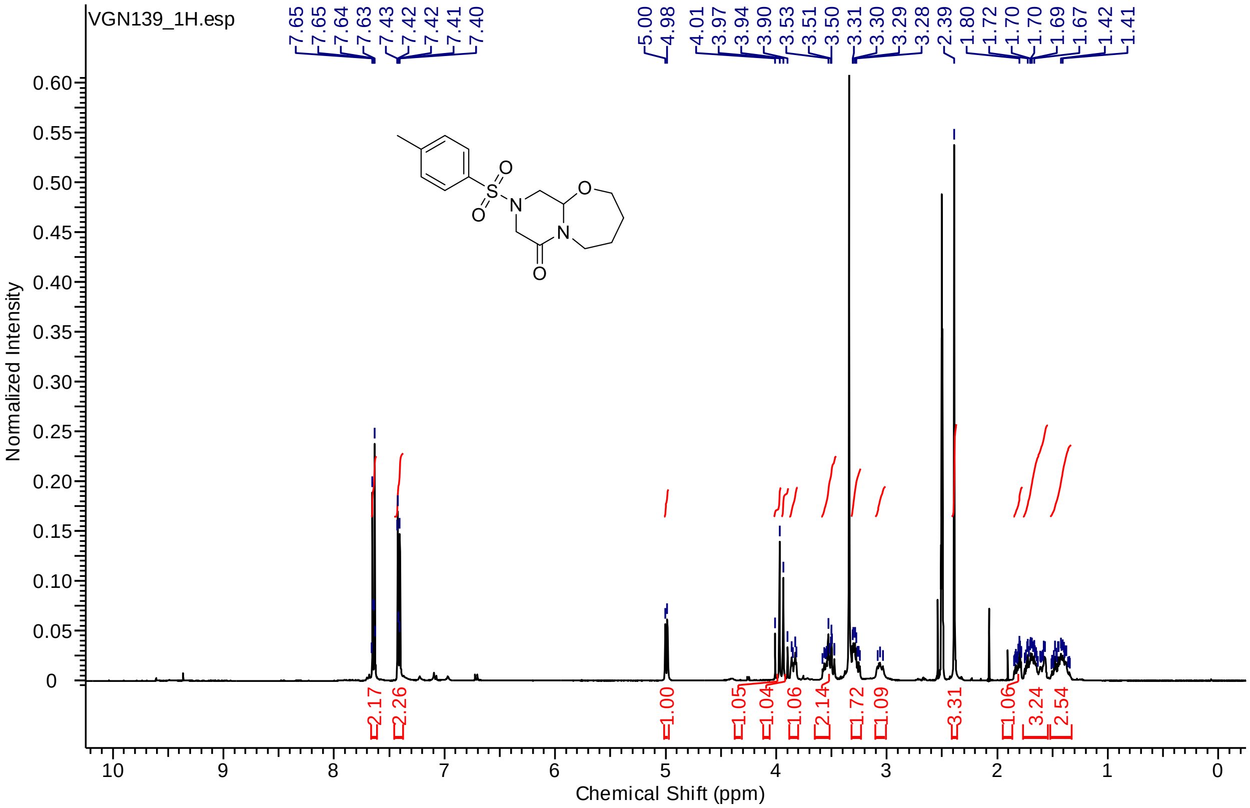

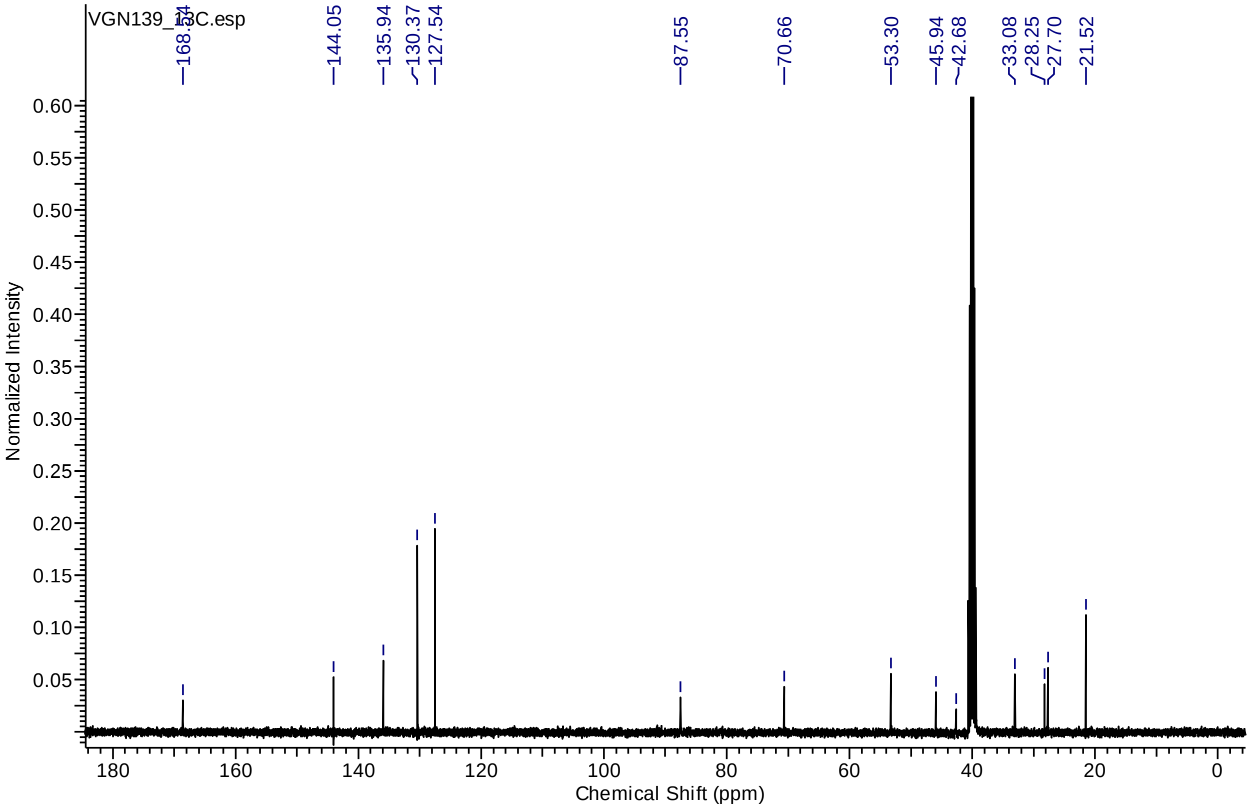

^{1}H and ^{13}C NMR spectra (d_6-DMSO) for compound 6{*3,2,1,3*}

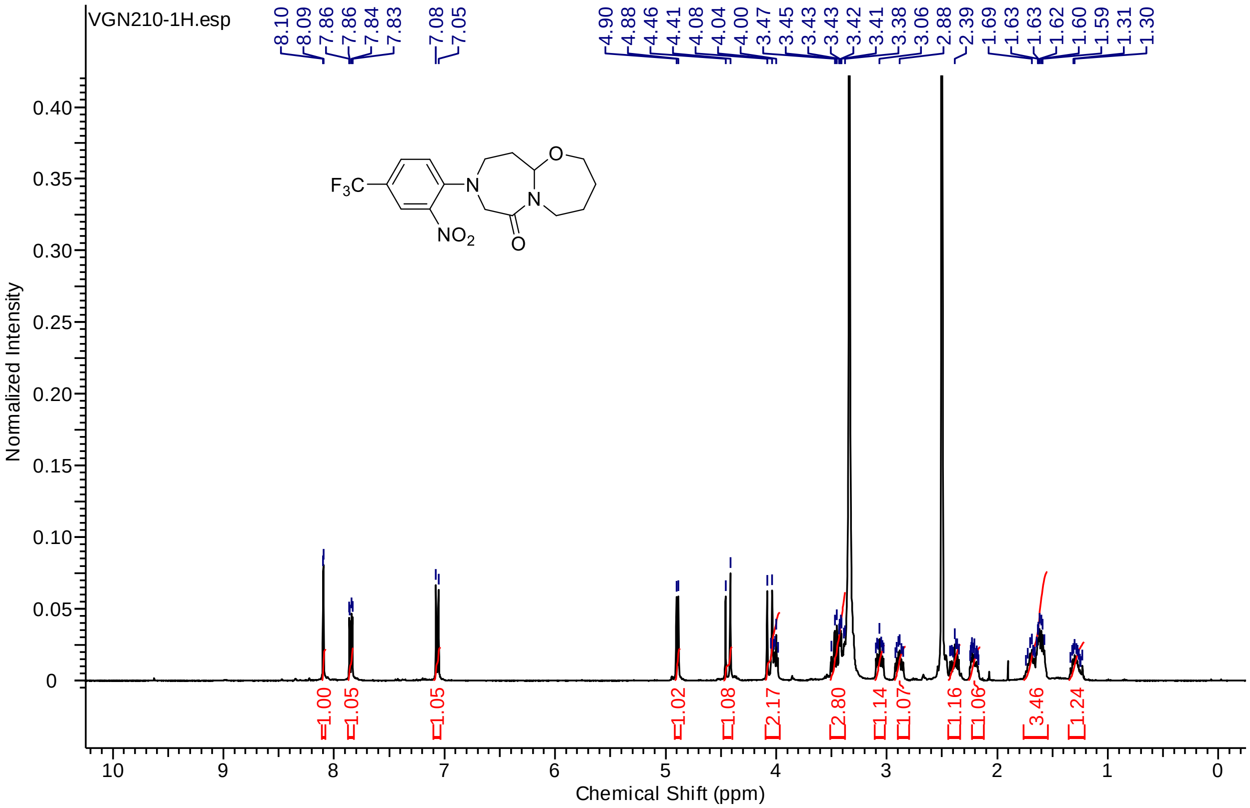

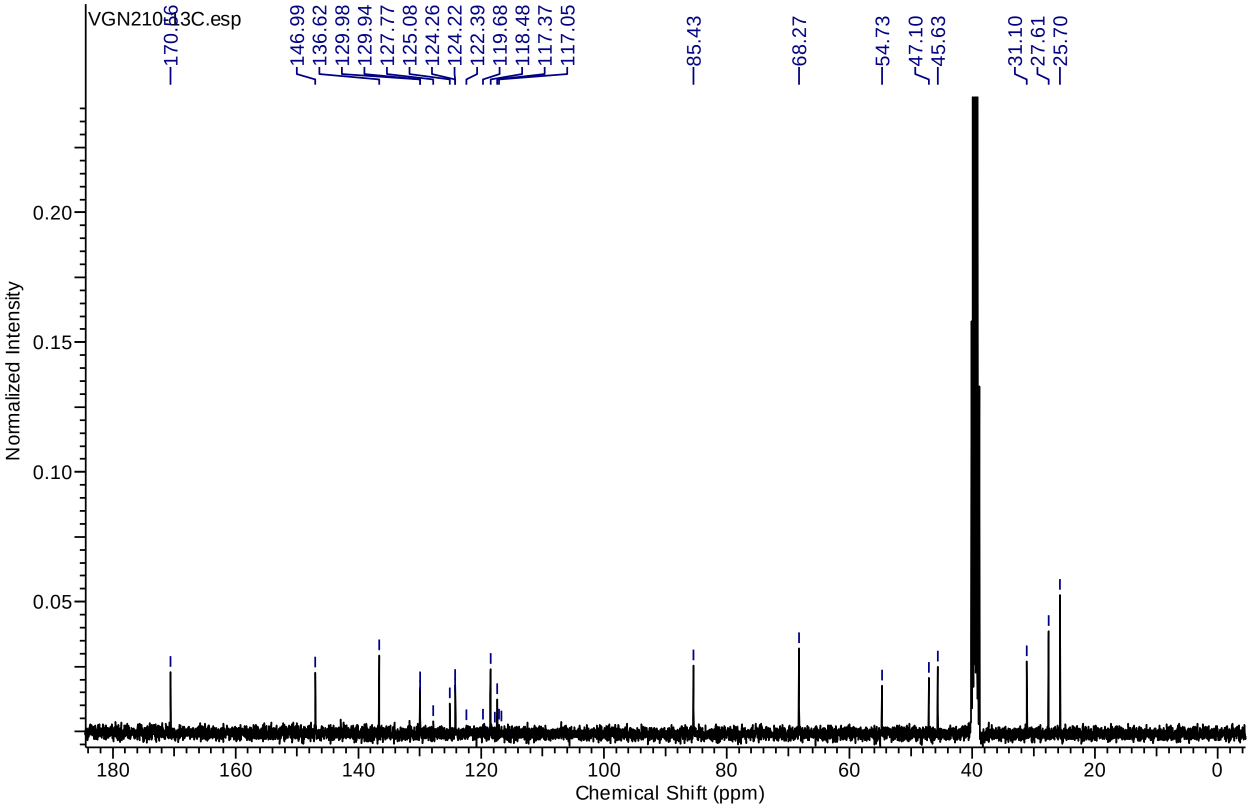

^{1}H and ^{13}C NMR spectra (d_6-DMSO) for compound 7{*1,3,1,3*}

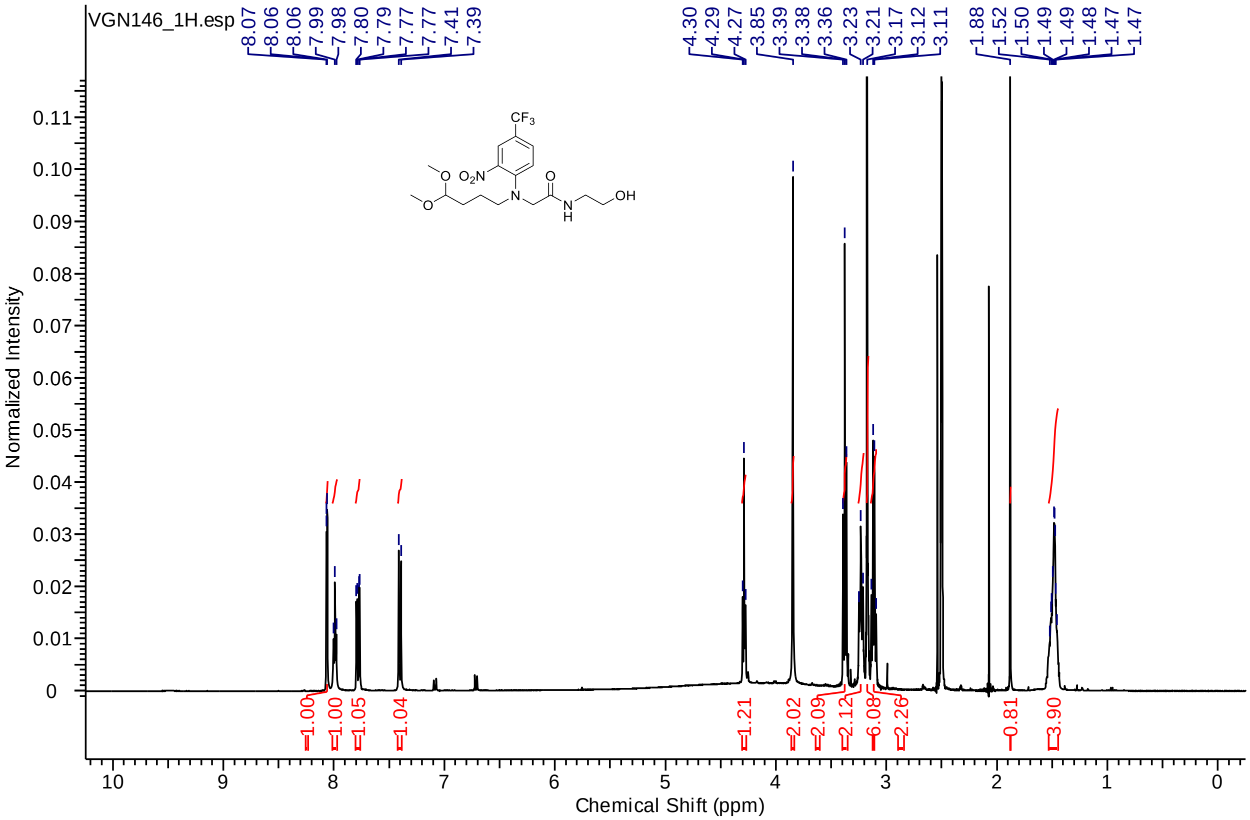

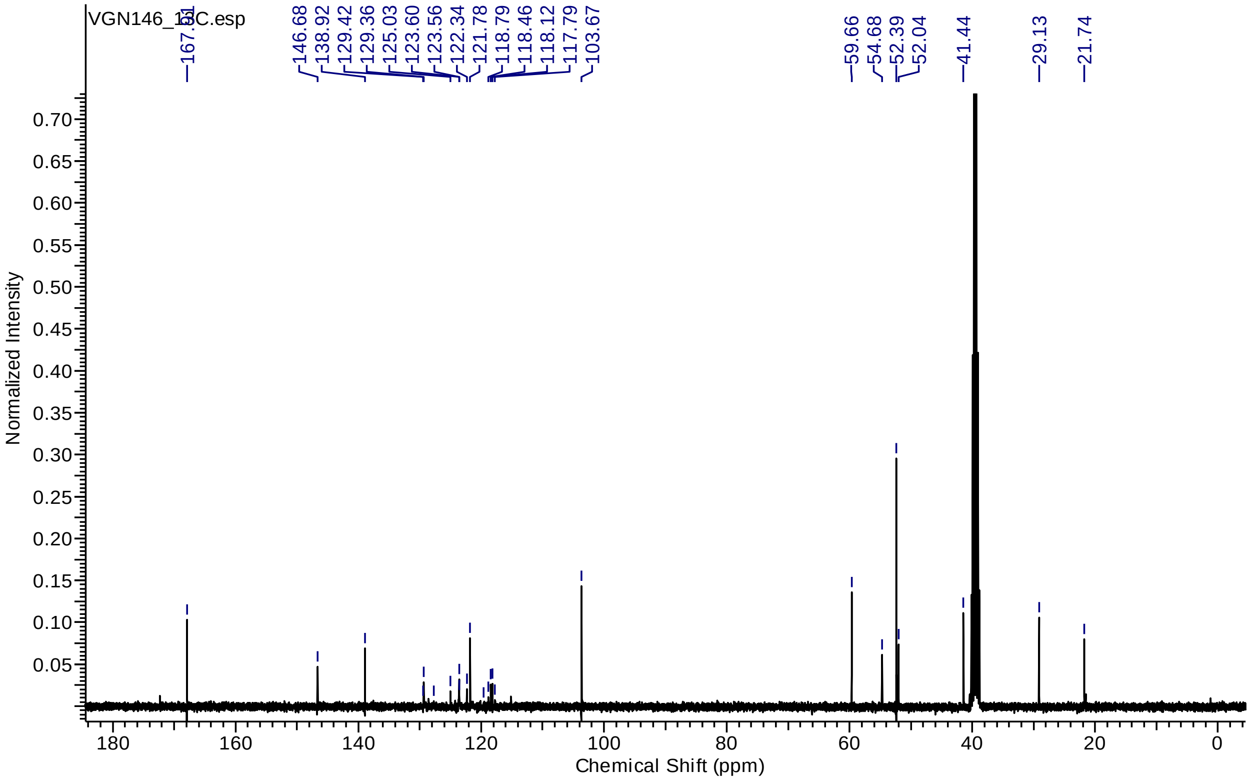

^{1}H and ^{13}C NMR spectra (d_6-DMSO) for compound 7{*2,3,1,3*}

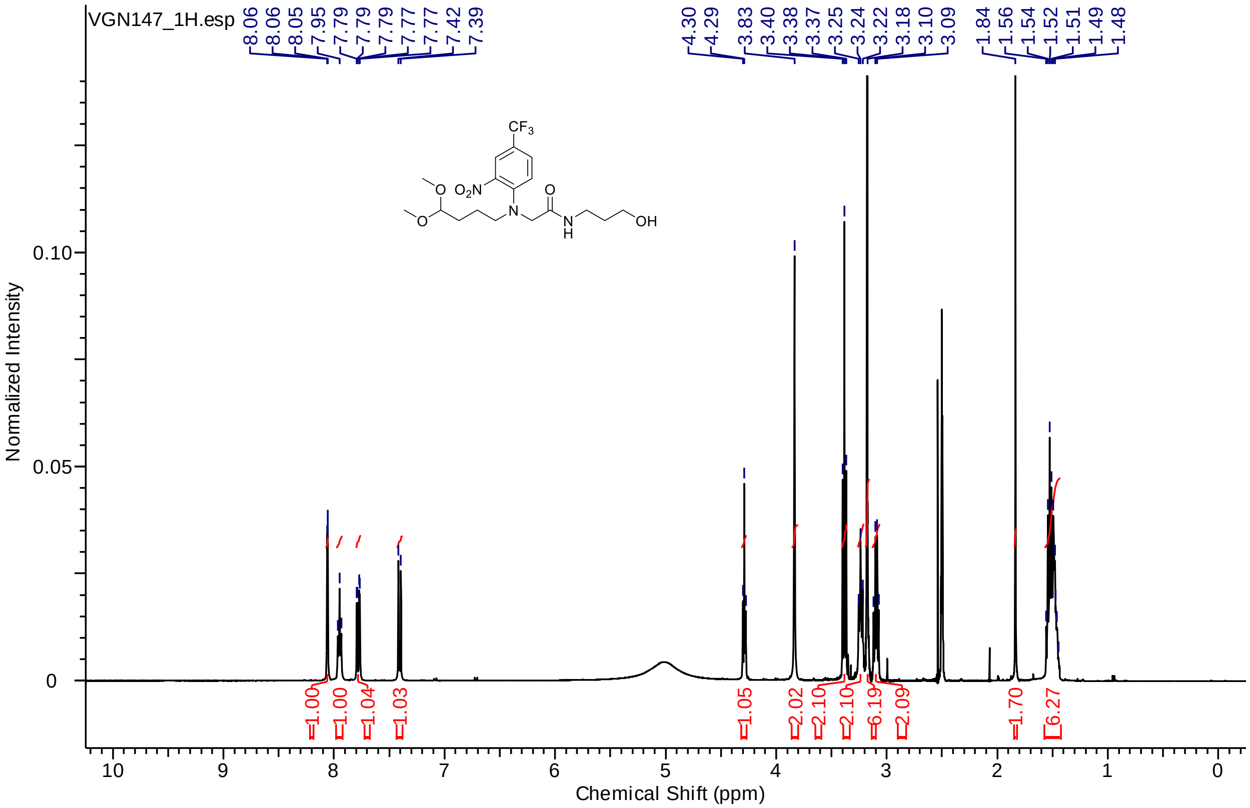

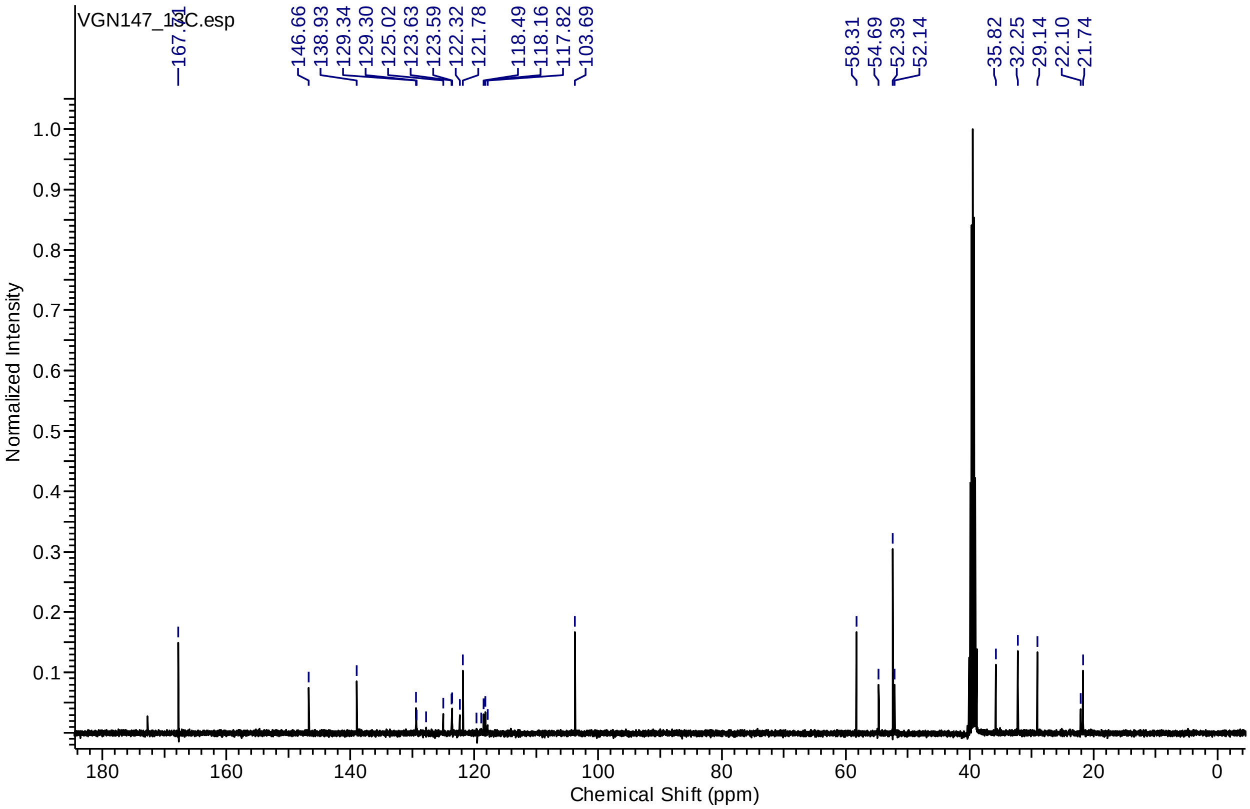

^{1}H and ^{13}C NMR spectra (d_6-DMSO) for compound 10{*1,1*}

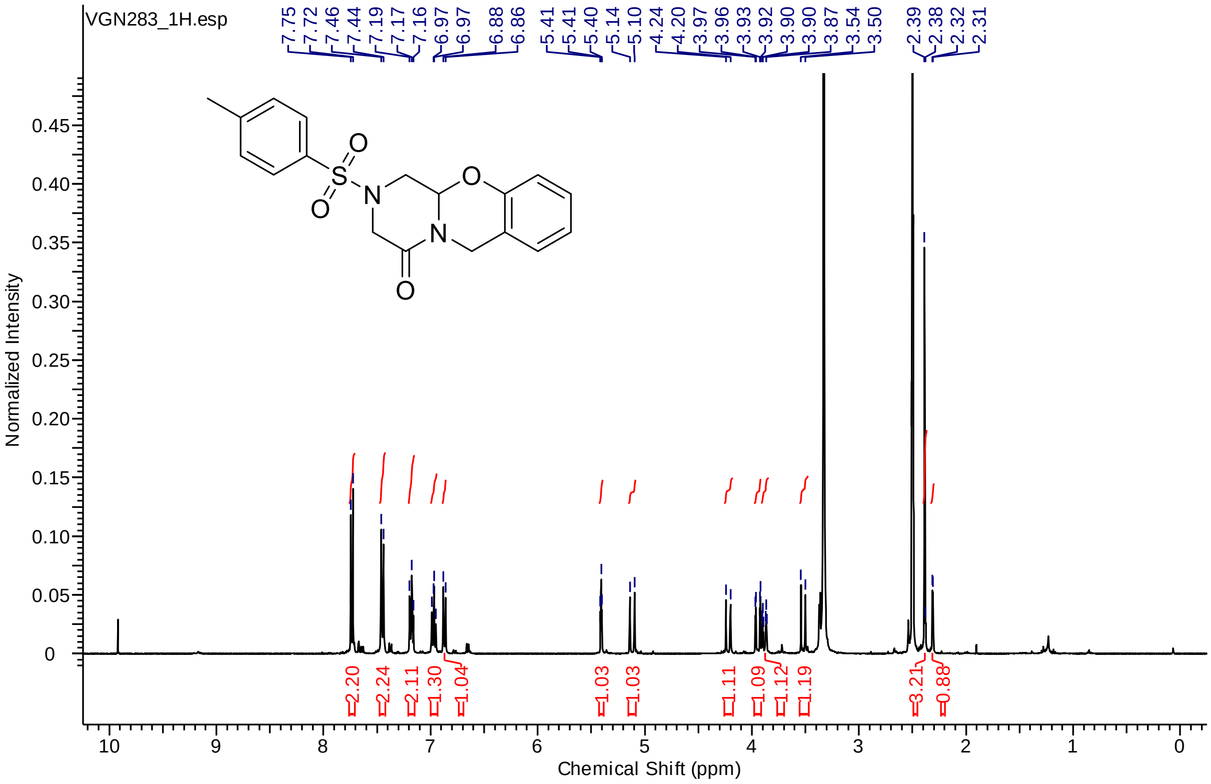

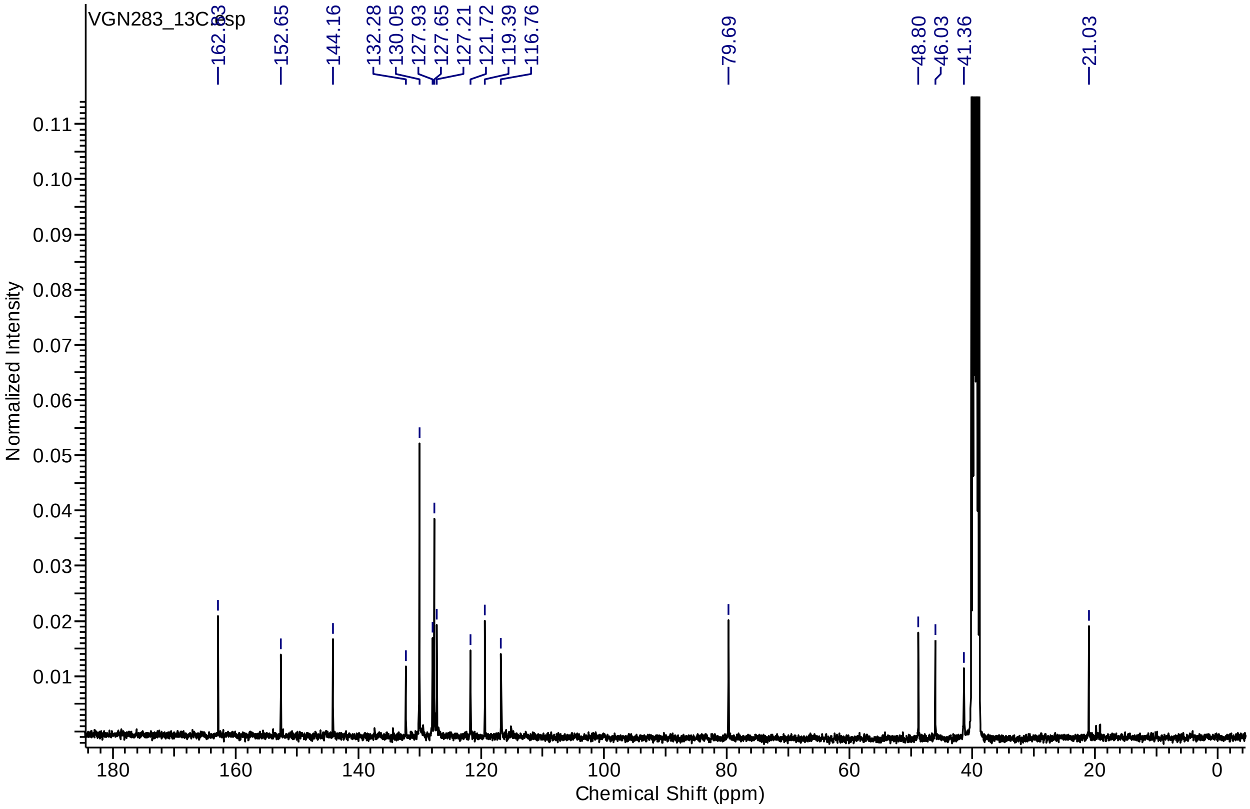

^{1}H and ^{13}C NMR spectra (d_6-DMSO) for compound 10{*1,2*}

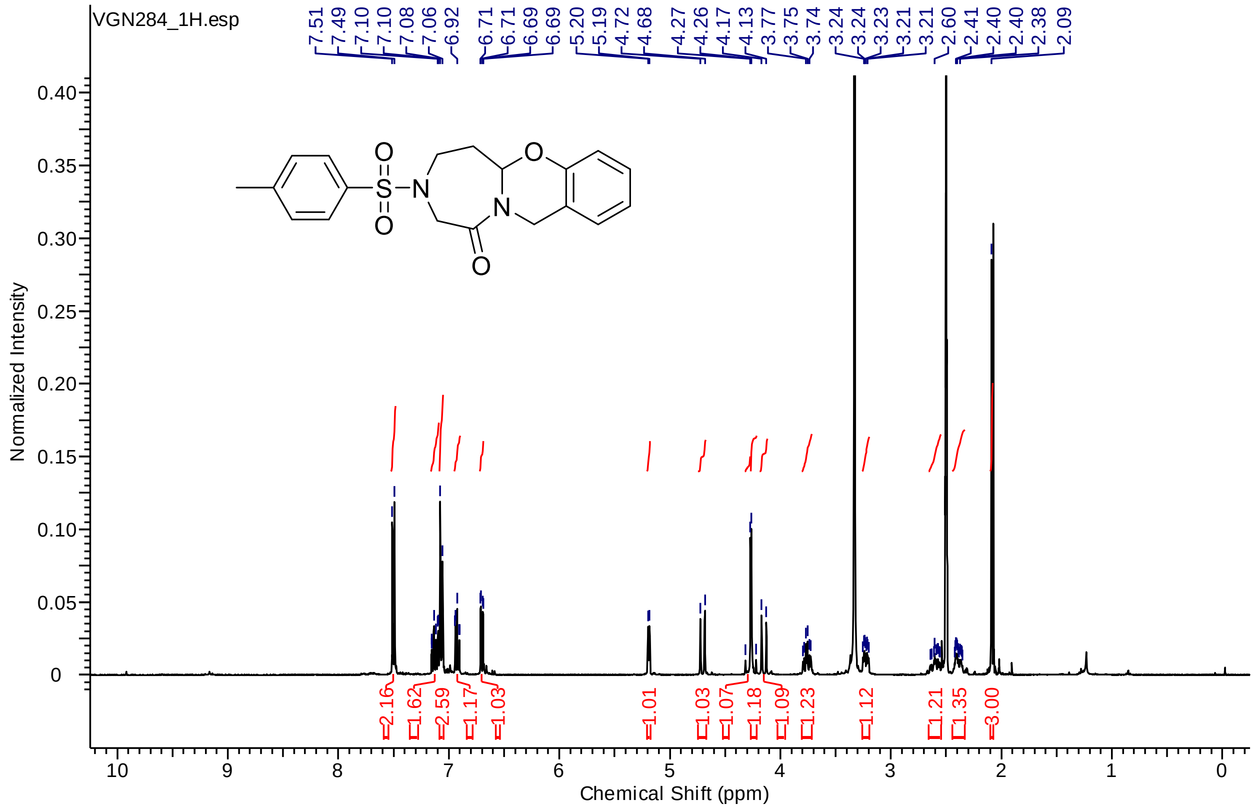

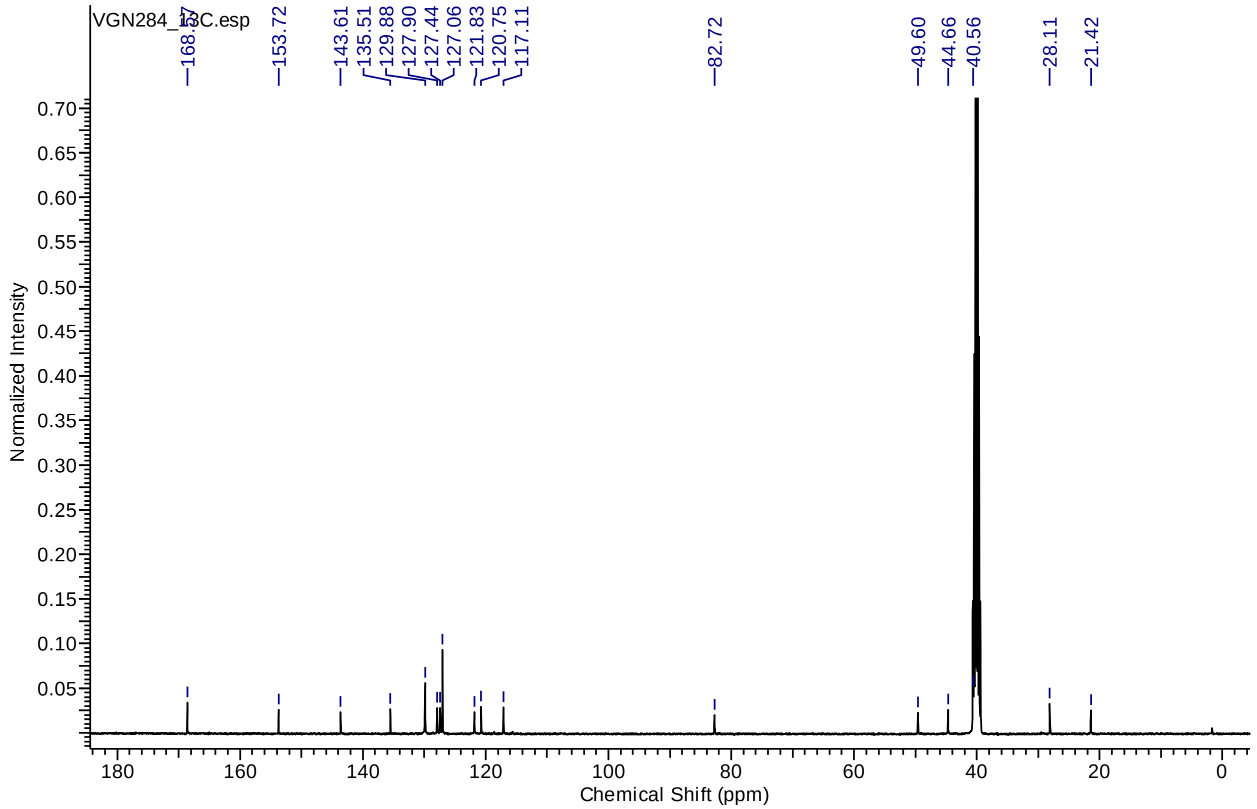

^{1}H and ^{13}C NMR spectra (d_6-DMSO) for compound 10{*2,1*}

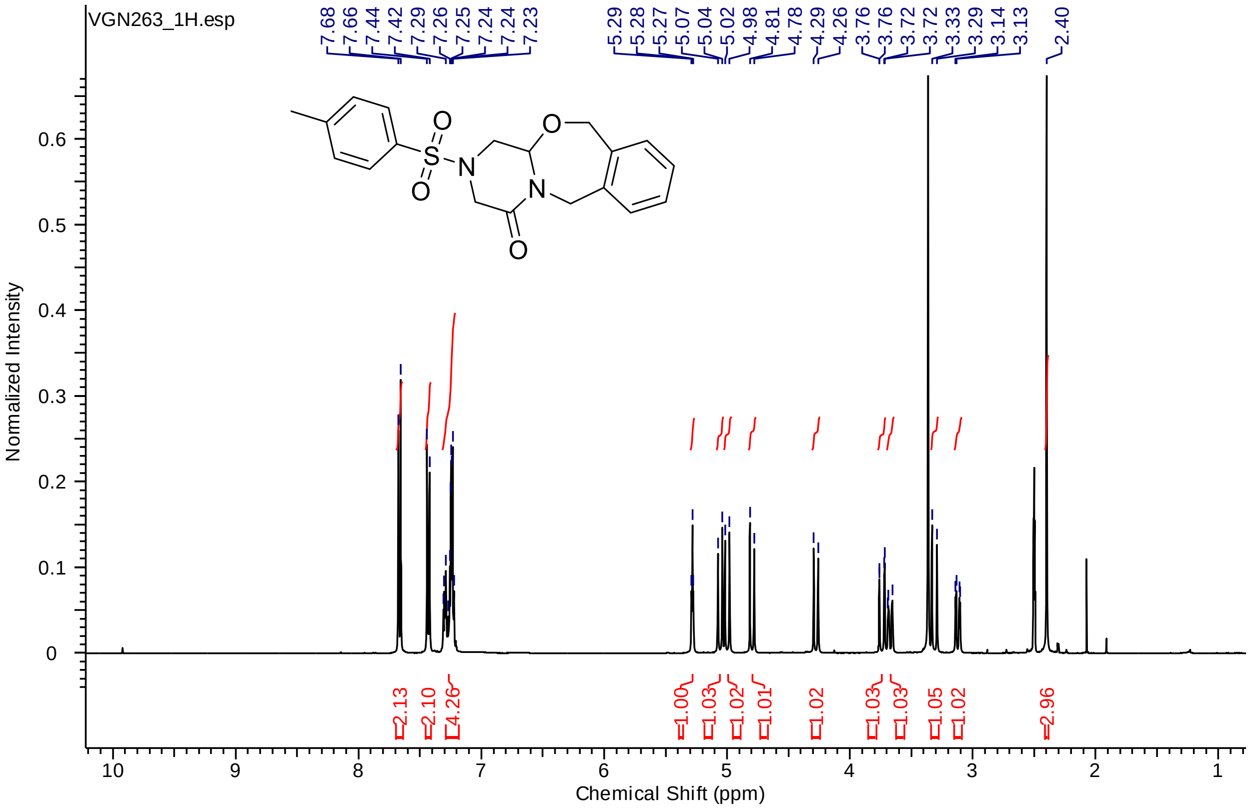

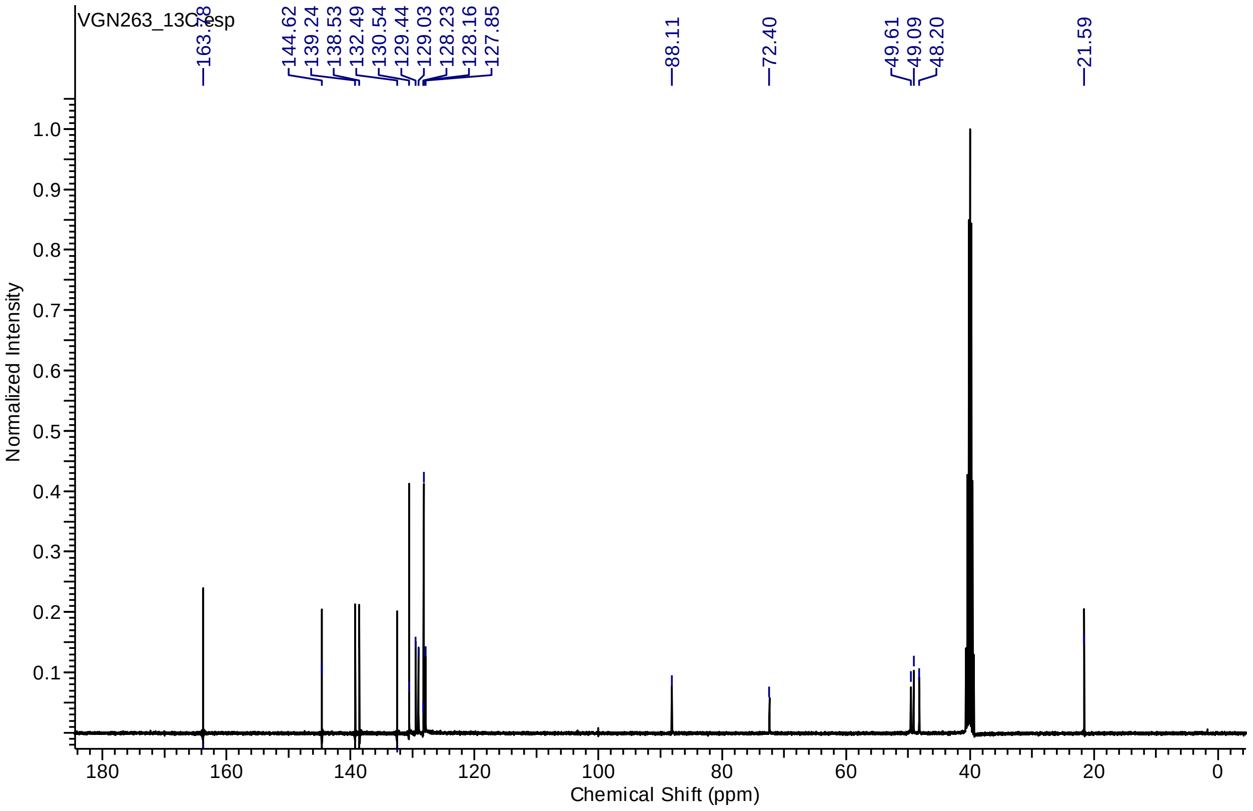

^{1}H and ^{13}C NMR spectra (d_6-DMSO) for compound 10{*2,2*}

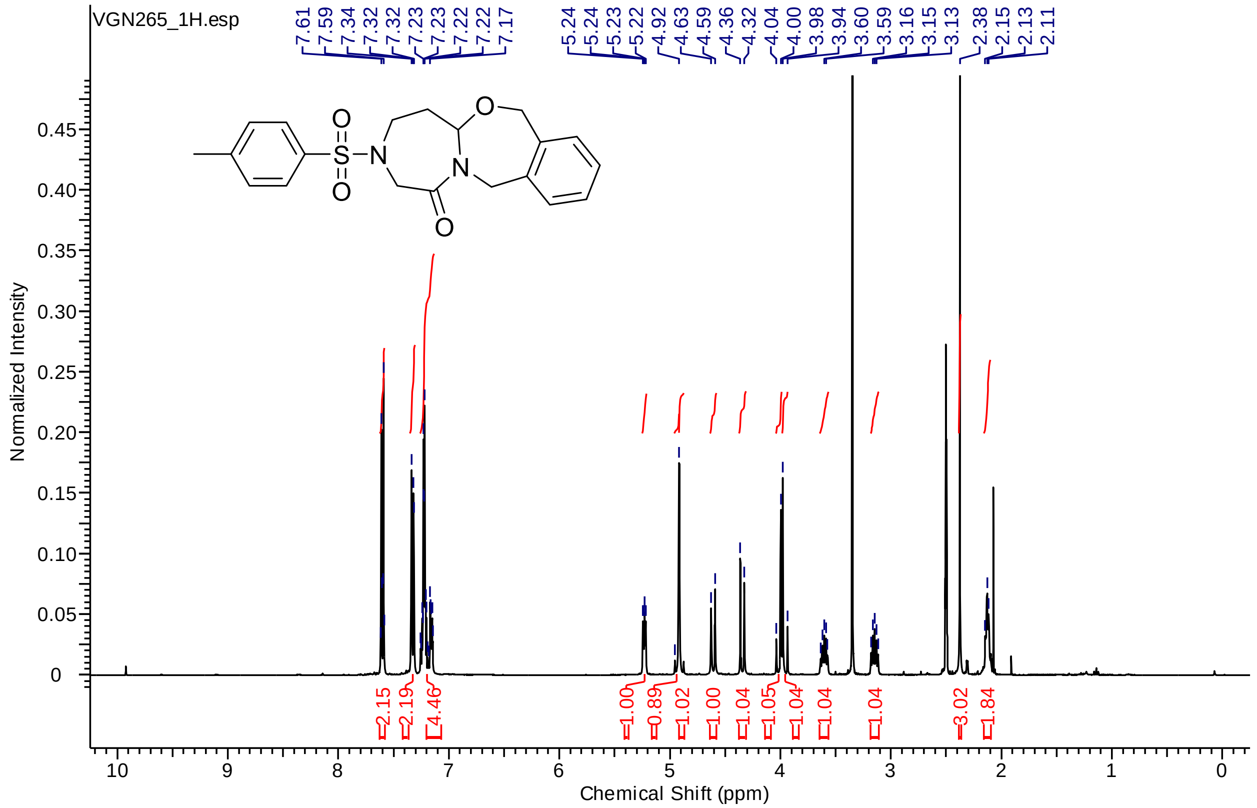

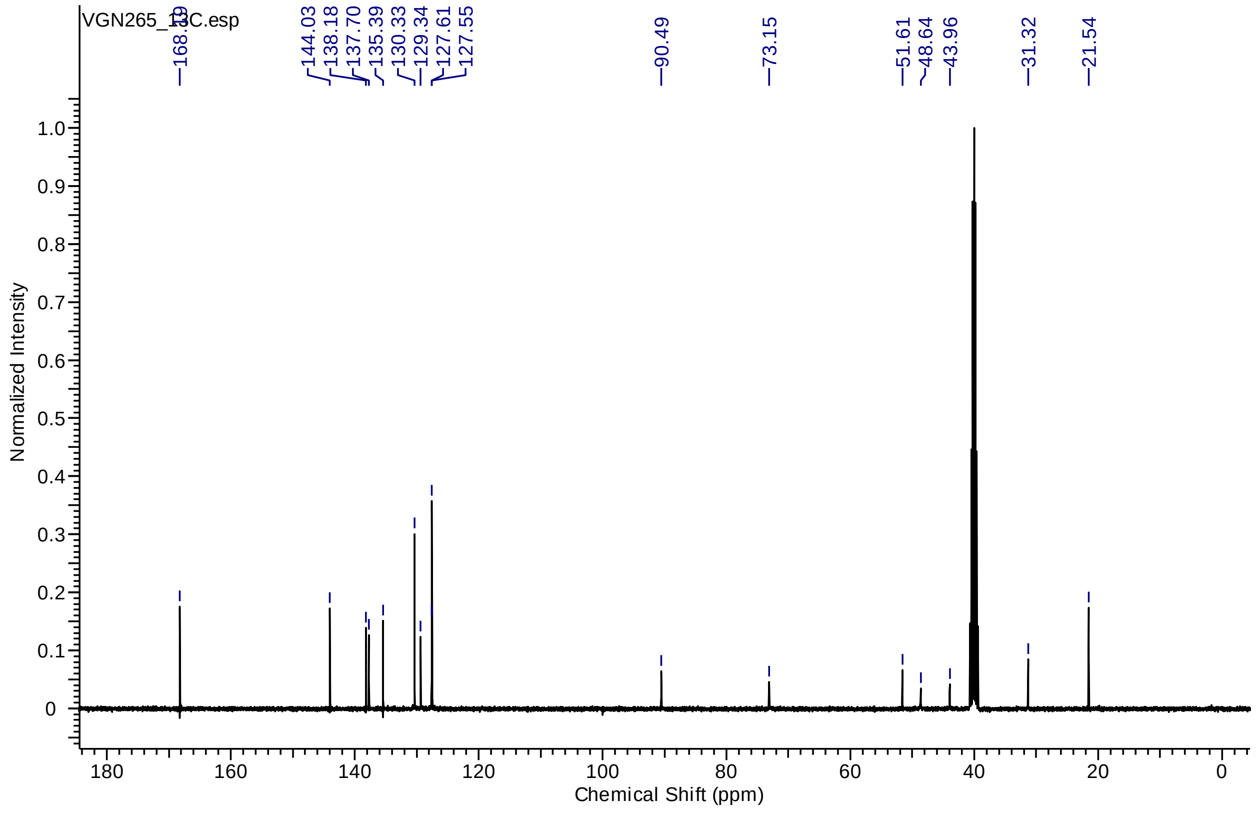

^{1}H and ^{13}C NMR spectra (d_6-DMSO) for compound 15{*1*}

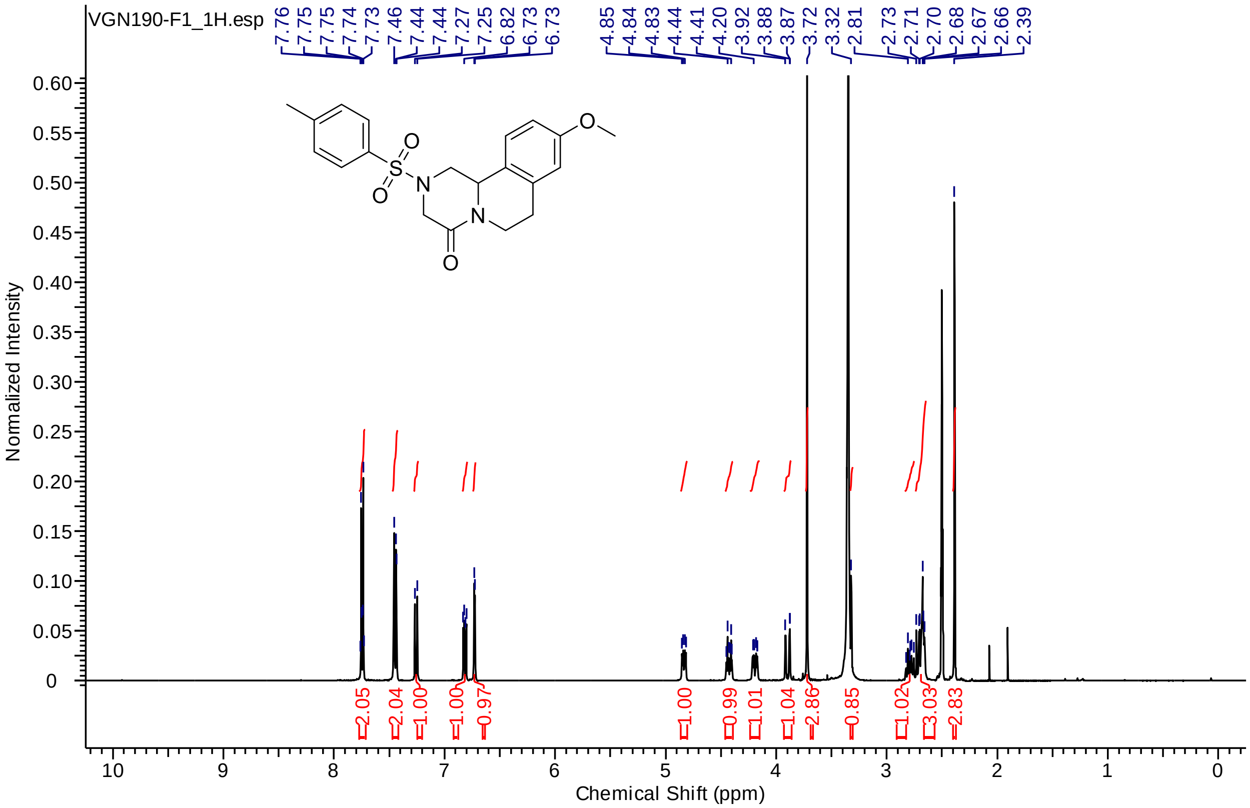

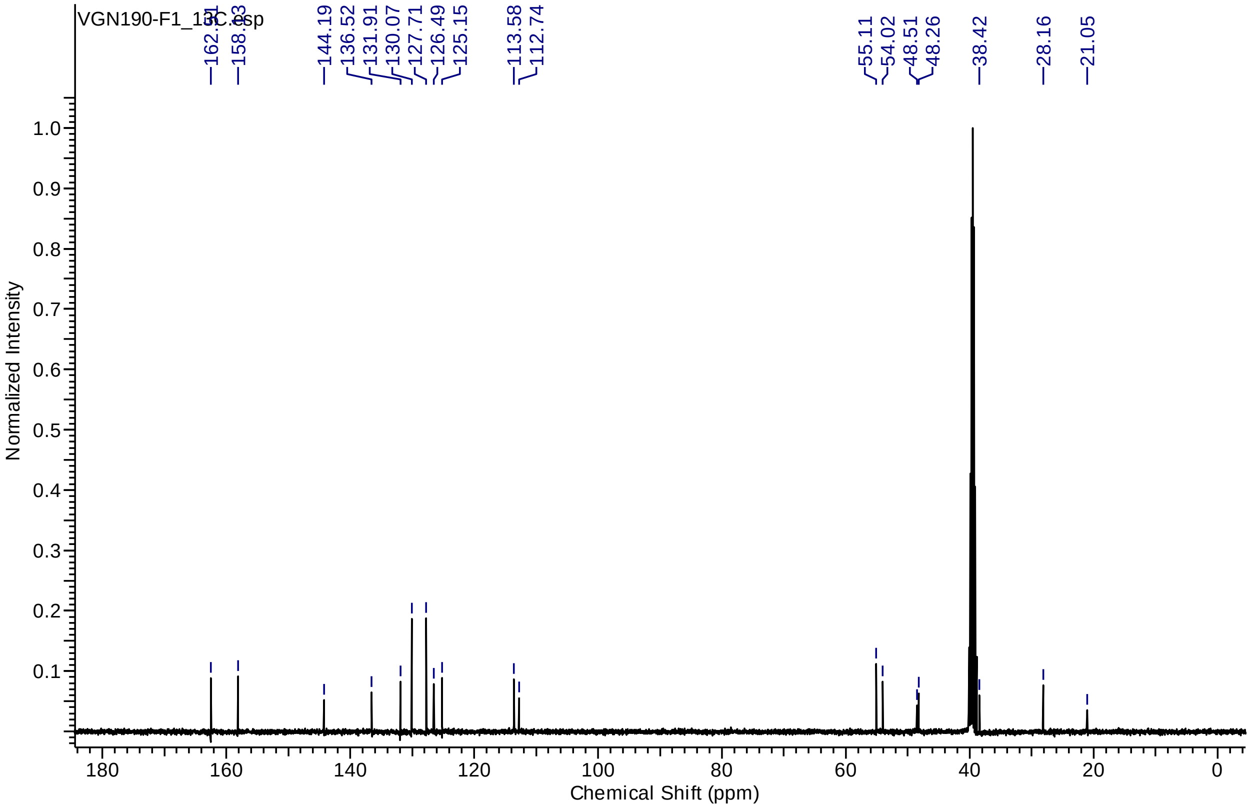

^{1}H and ^{13}C NMR spectra (d_6-DMSO) for compound 15{*2*}

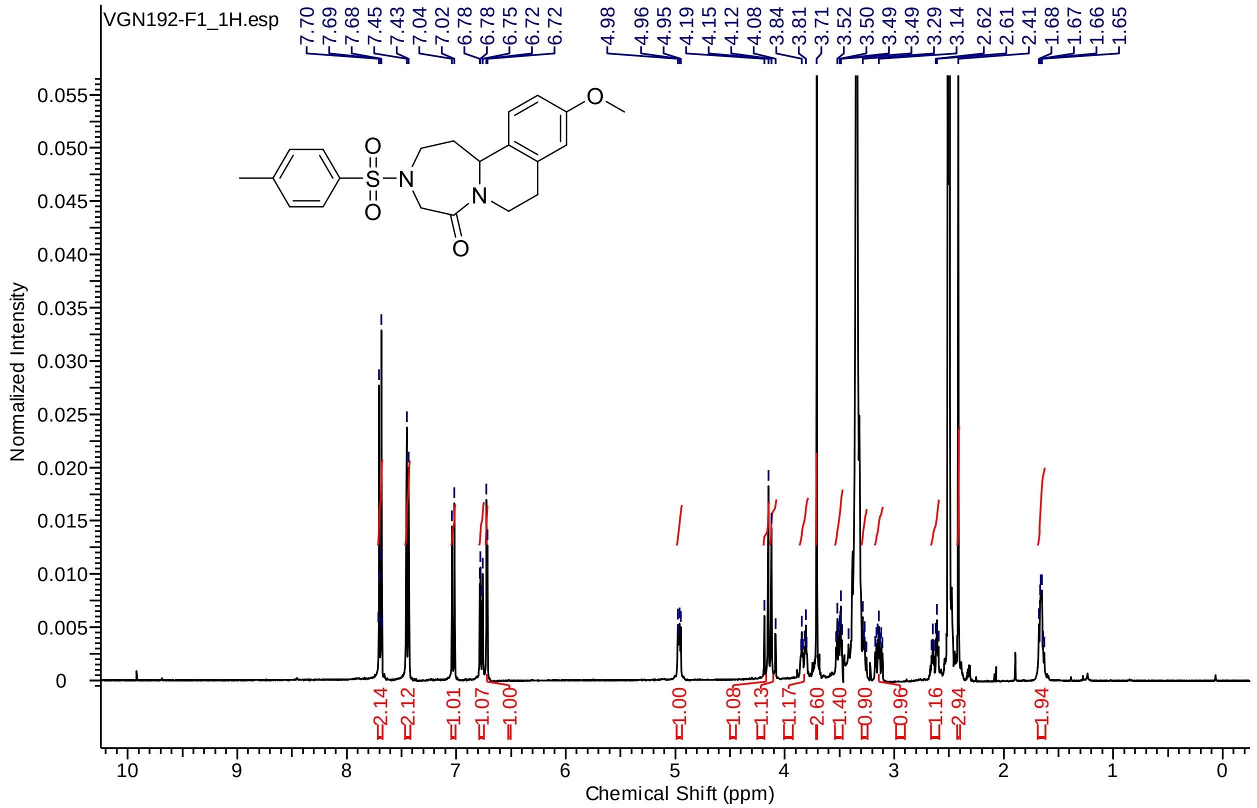

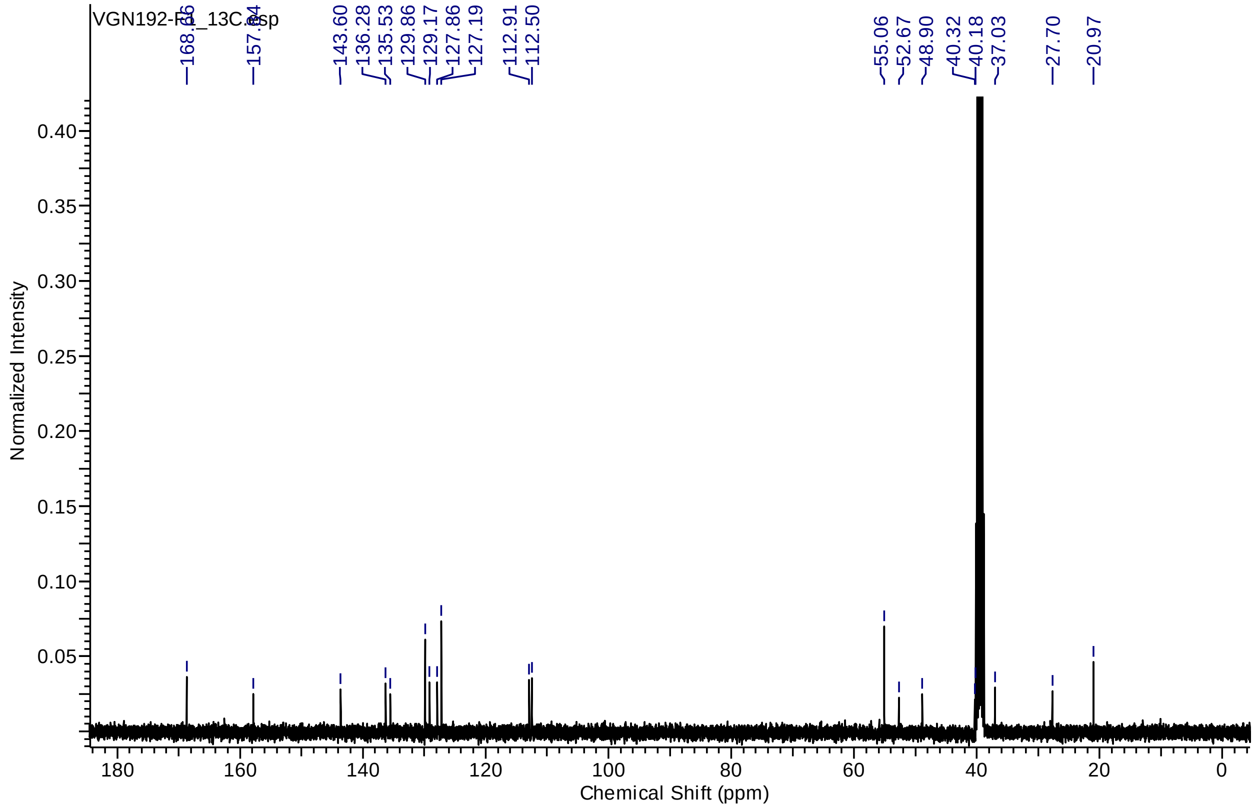

^{1}H and ^{13}C NMR spectra (d_6-DMSO) for compound 16{*1*}

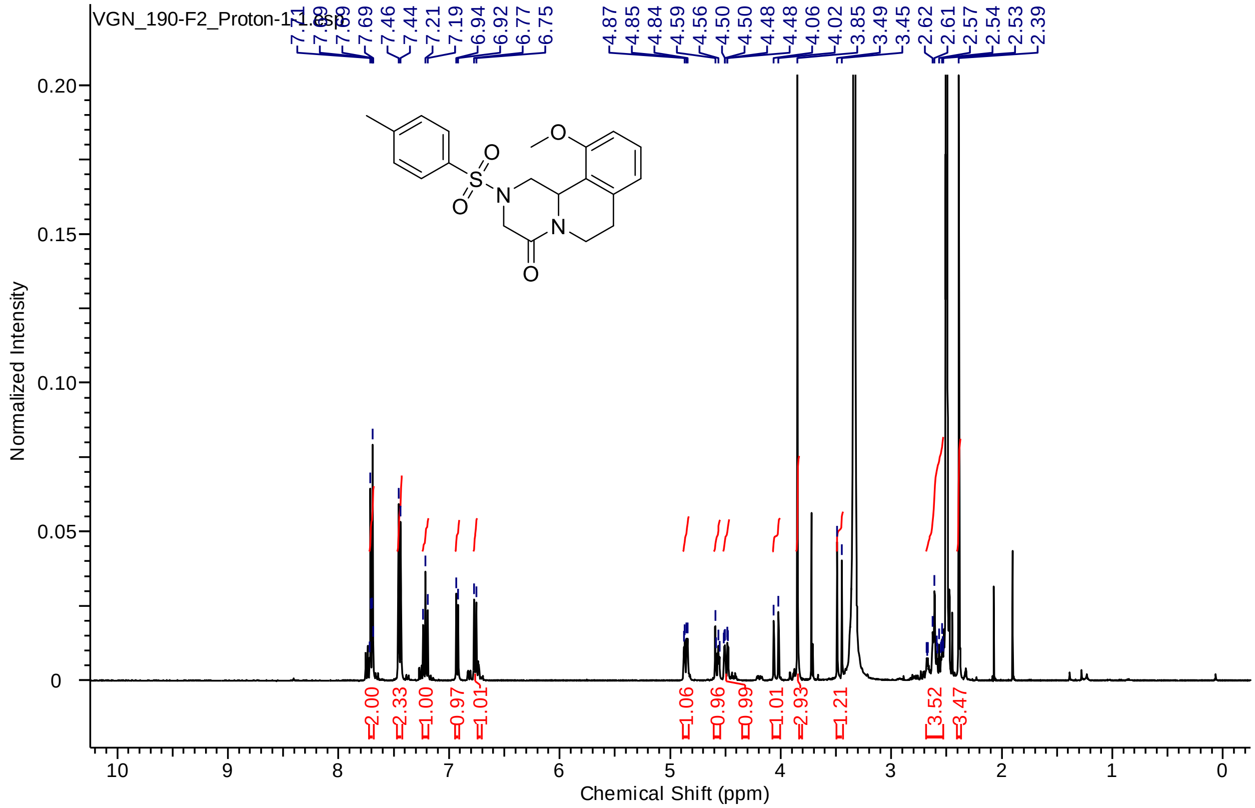

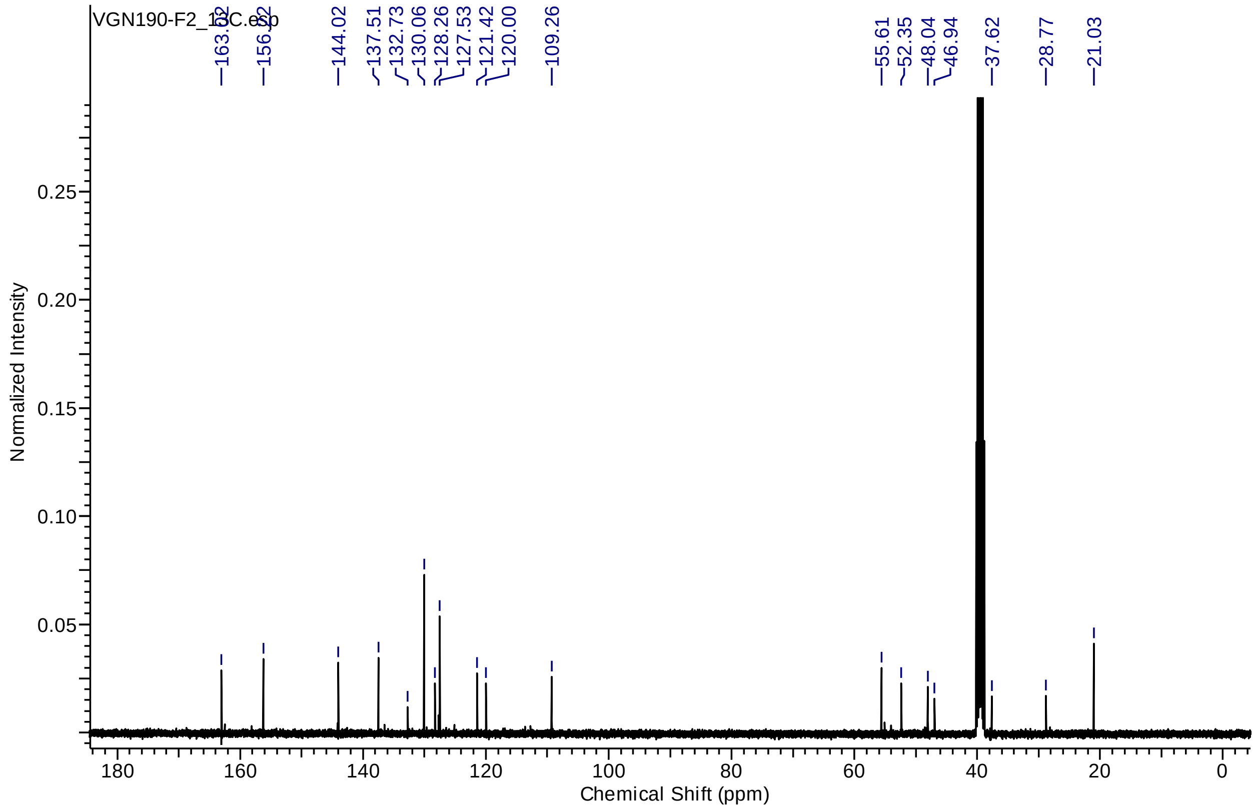

Supplement: Supplementary file 1 [file molecules-23-01090-s001.pdf]
